# Supplementary figures and images for: Phase transition of WTAP regulates m6A modification of interferon-stimulated genes (part 1 of 2)
Source: eLife. 2025 May 27;13:RP100601. doi: 10.7554/eLife.100601 (PMC12113268; doi:10.7554/eLife.100601)

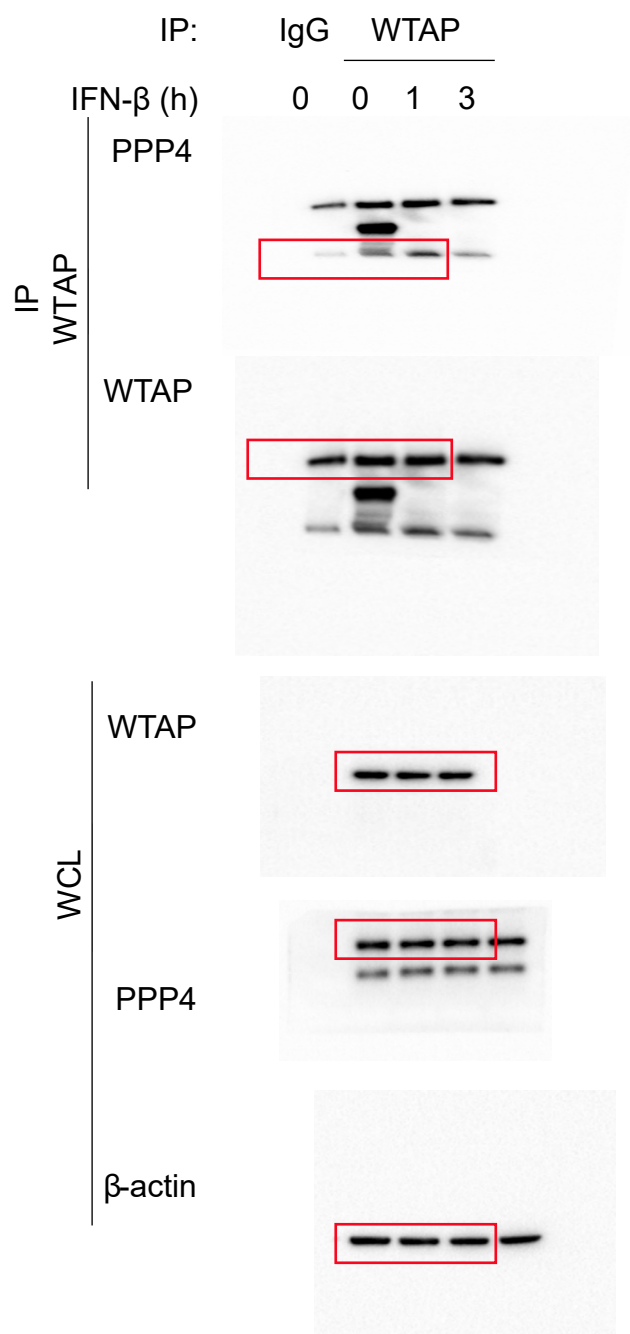

Figure 2B, source data 1: Original membranes corresponding to Figure 2B.

Supplement: Figure 2—source data 1. [file elife-100601-fig2-data1.zip › Figure 2-Source Data 1/Figure 2B.pdf]

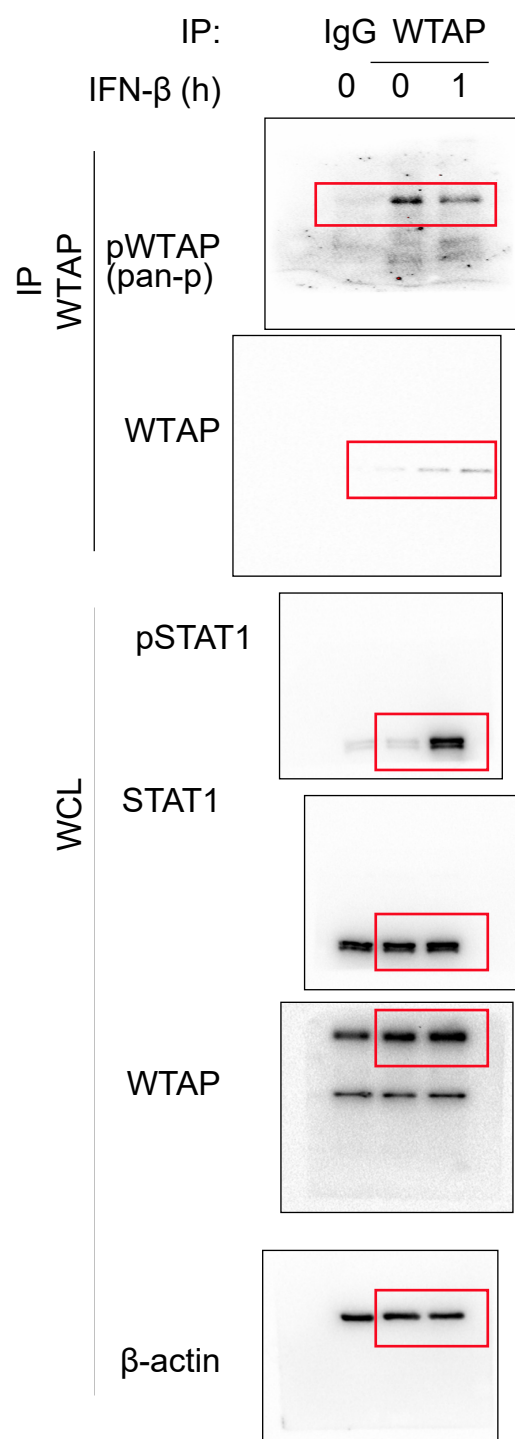

Figure 2A, source data 1: Original membranes corresponding to Figure 2A.

Supplement: Figure 2—source data 1. [file elife-100601-fig2-data1.zip › Figure 2-Source Data 1/Figure 2A.pdf]

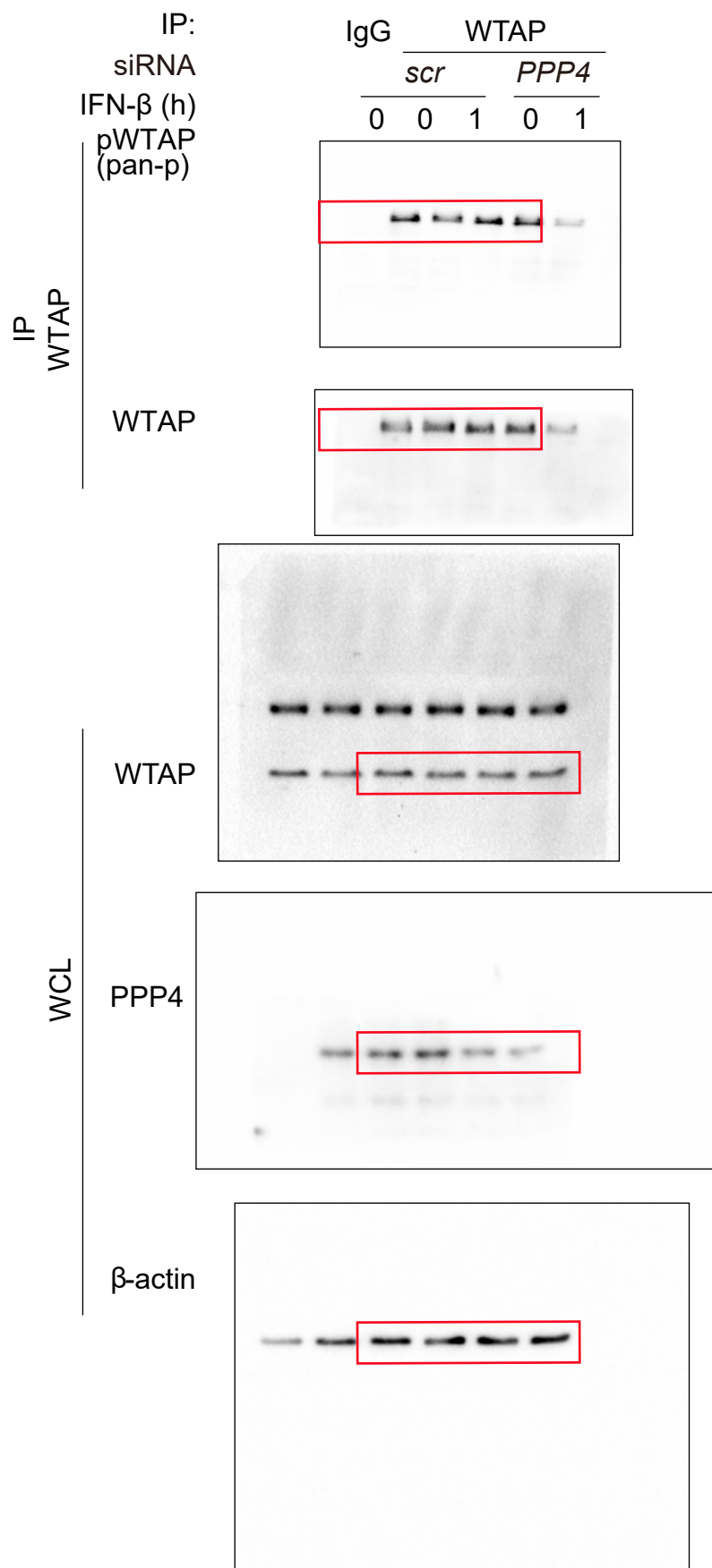

Figure 2C, source data 1: Original membranes corresponding to Figure 2C.

Supplement: Figure 2—source data 1. [file elife-100601-fig2-data1.zip › Figure 2-Source Data 1/Figure 2C.pdf]

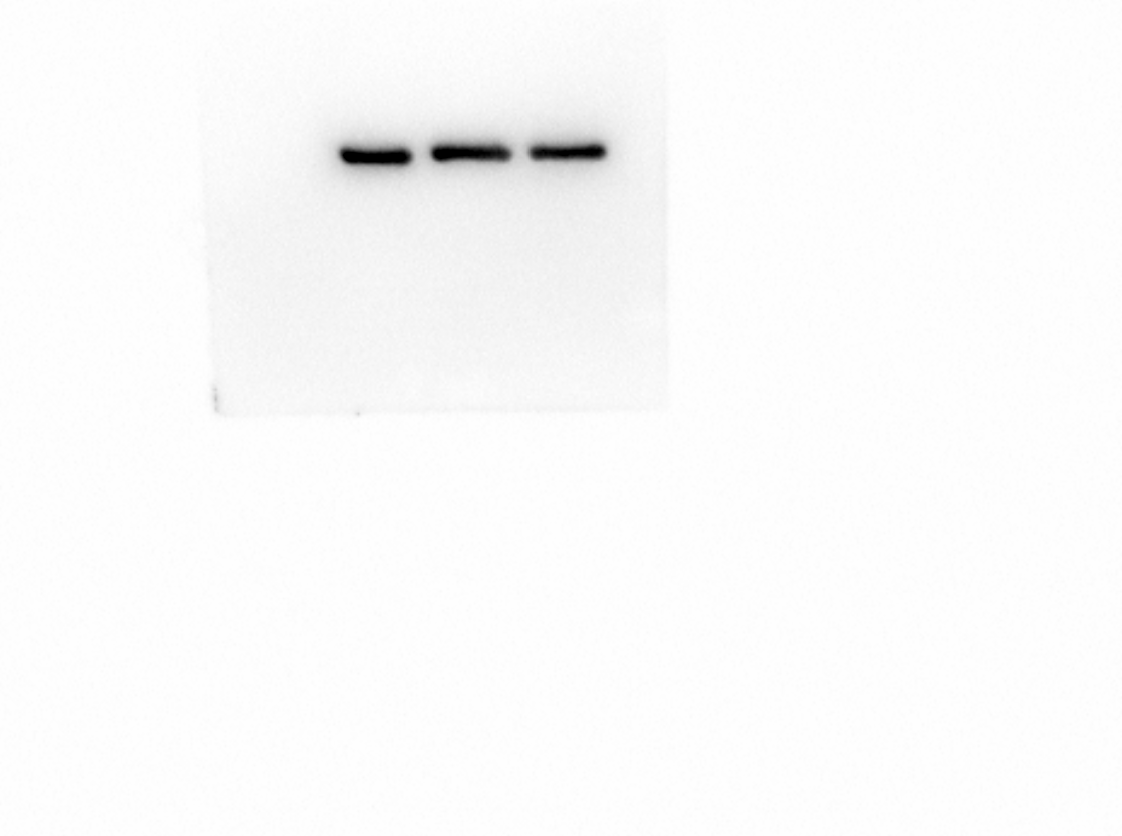

Supplement: Figure 2—source data 2. [file elife-100601-fig2-data2.zip › Figure 2-Source Data 2/Figure2a/actin.tif]

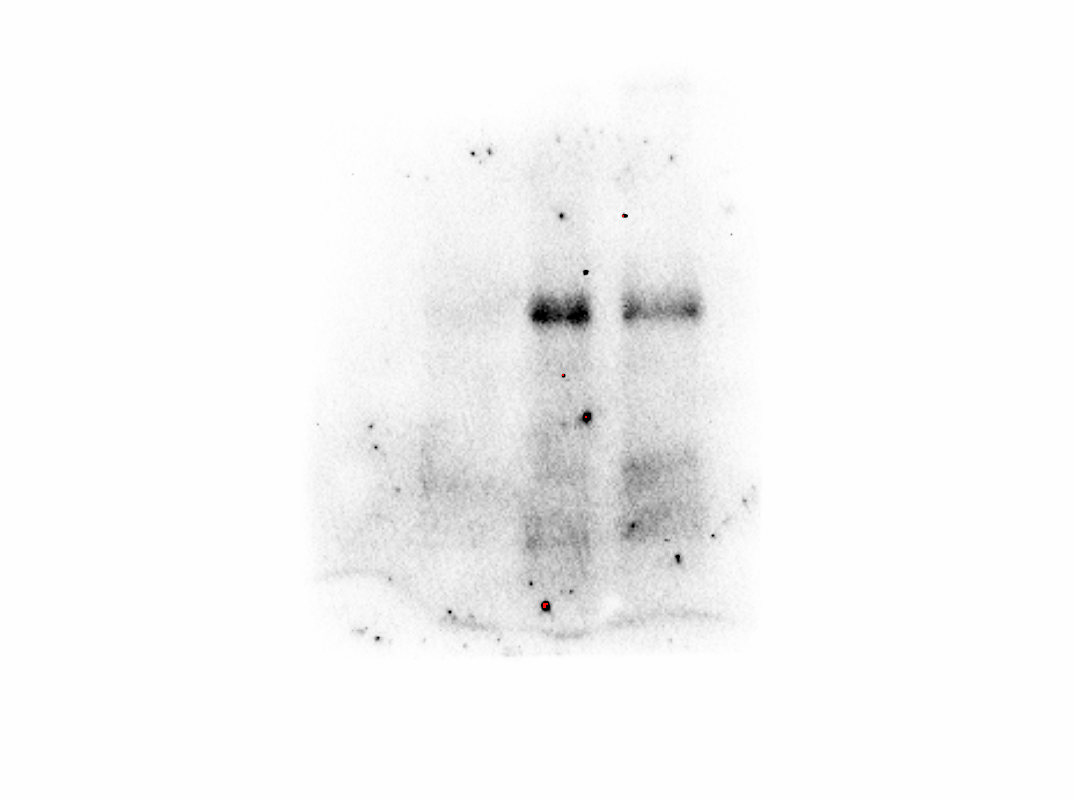

Supplement: Figure 2—source data 2. [file elife-100601-fig2-data2.zip › Figure 2-Source Data 2/Figure2a/IP-pan-p.tif]

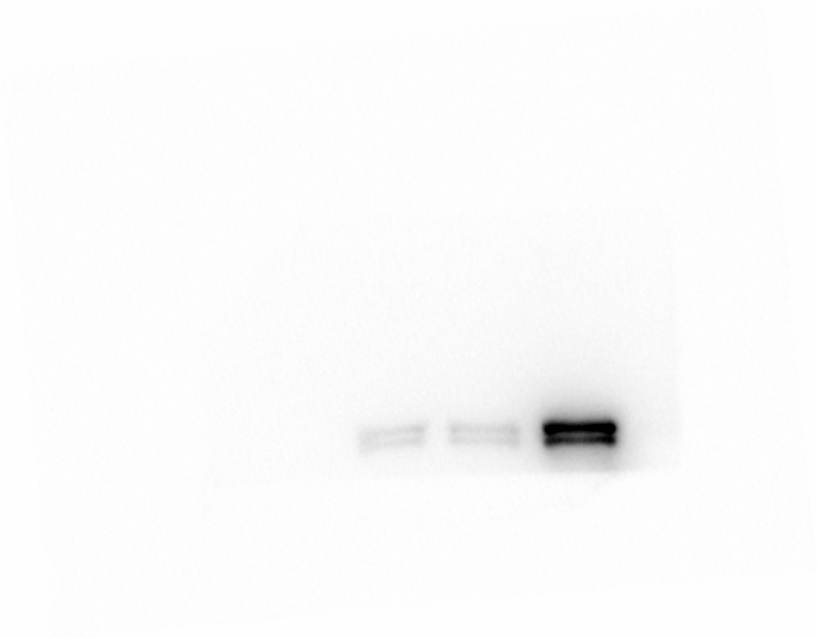

Supplement: Figure 2—source data 2. [file elife-100601-fig2-data2.zip › Figure 2-Source Data 2/Figure2a/pSTAT1.tif]

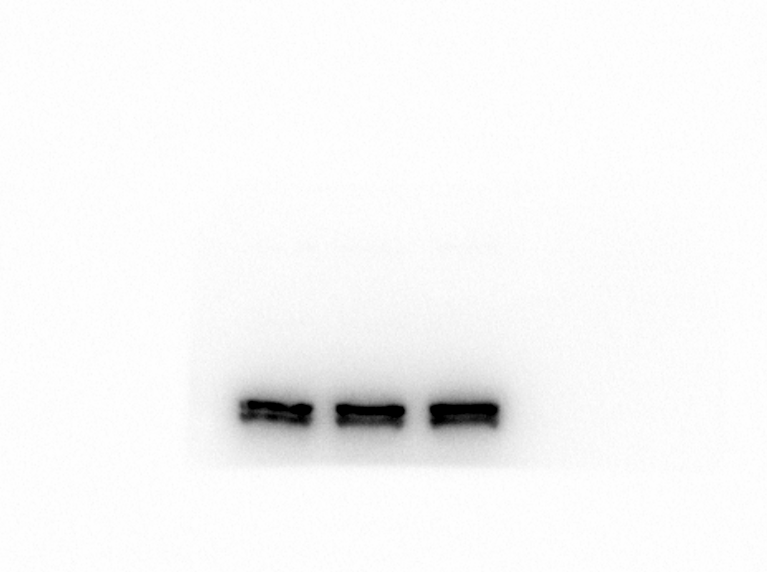

Supplement: Figure 2—source data 2. [file elife-100601-fig2-data2.zip › Figure 2-Source Data 2/Figure2a/STAT1.tif]

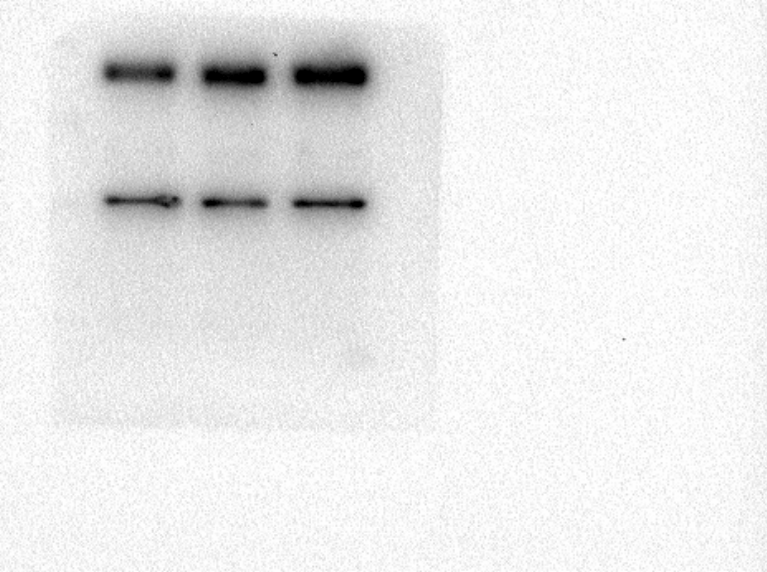

Supplement: Figure 2—source data 2. [file elife-100601-fig2-data2.zip › Figure 2-Source Data 2/Figure2a/WTAP.tif]

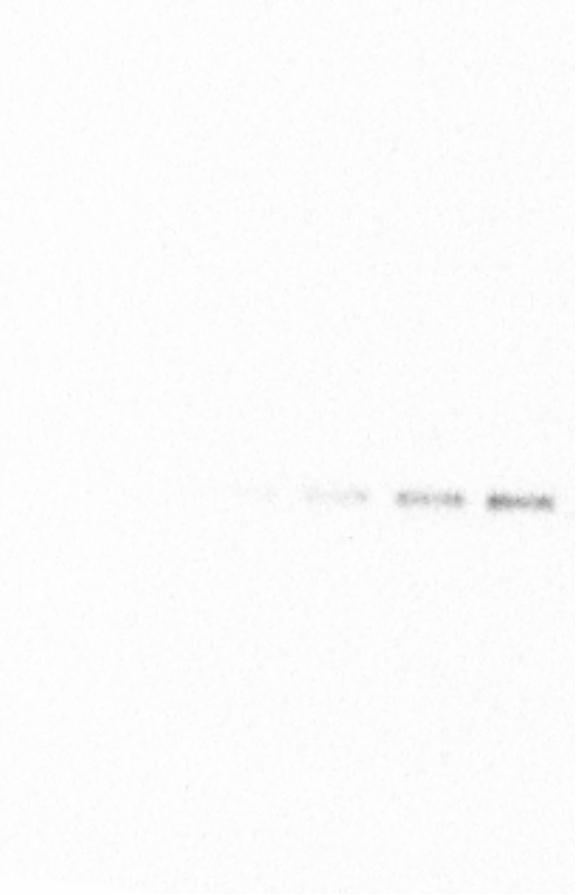

Supplement: Figure 2—source data 2. [file elife-100601-fig2-data2.zip › Figure 2-Source Data 2/Figure2a/IP-WTAP.tif]

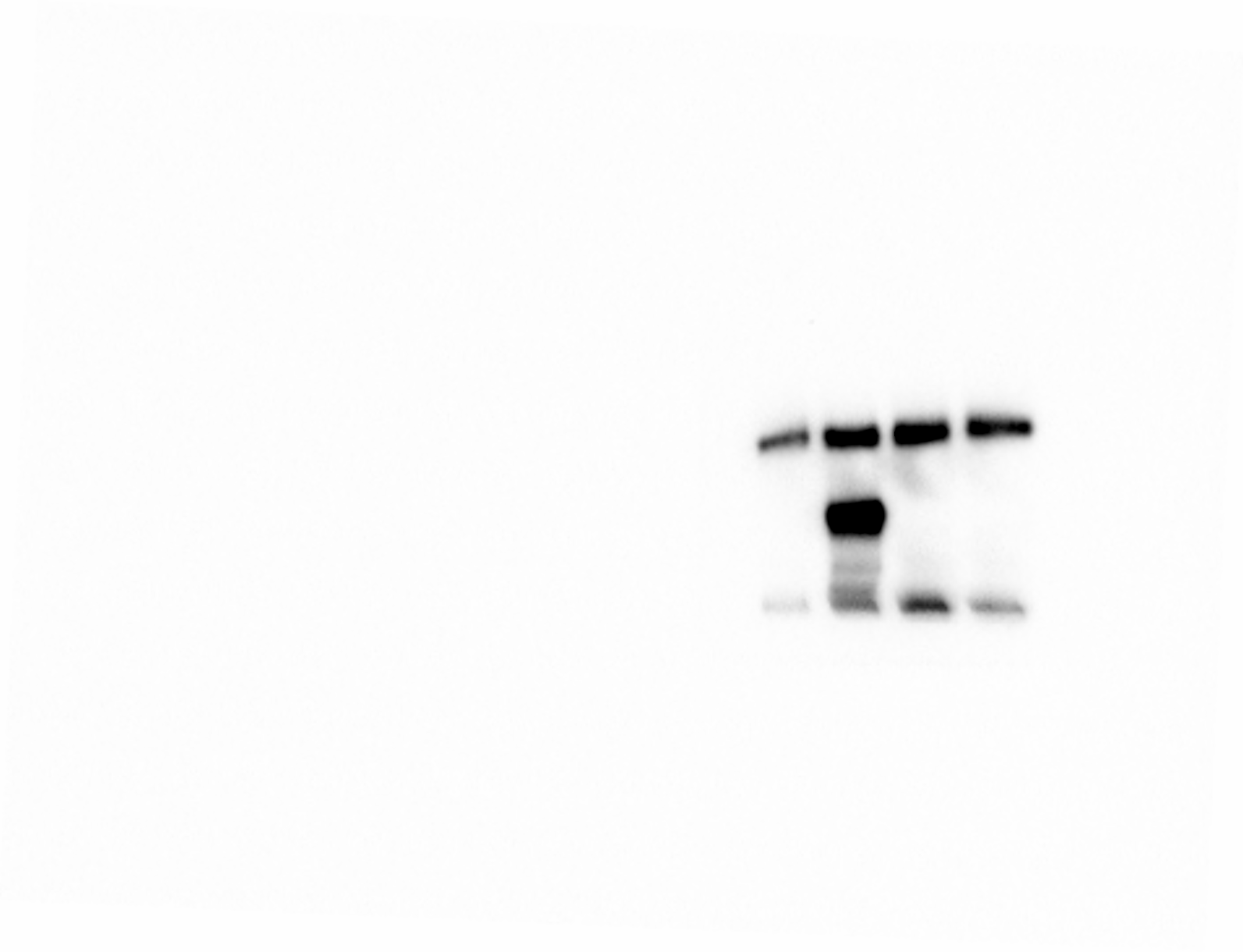

Supplement: Figure 2—source data 2. [file elife-100601-fig2-data2.zip › Figure 2-Source Data 2/Figure2b/IP-PPP4.tif]

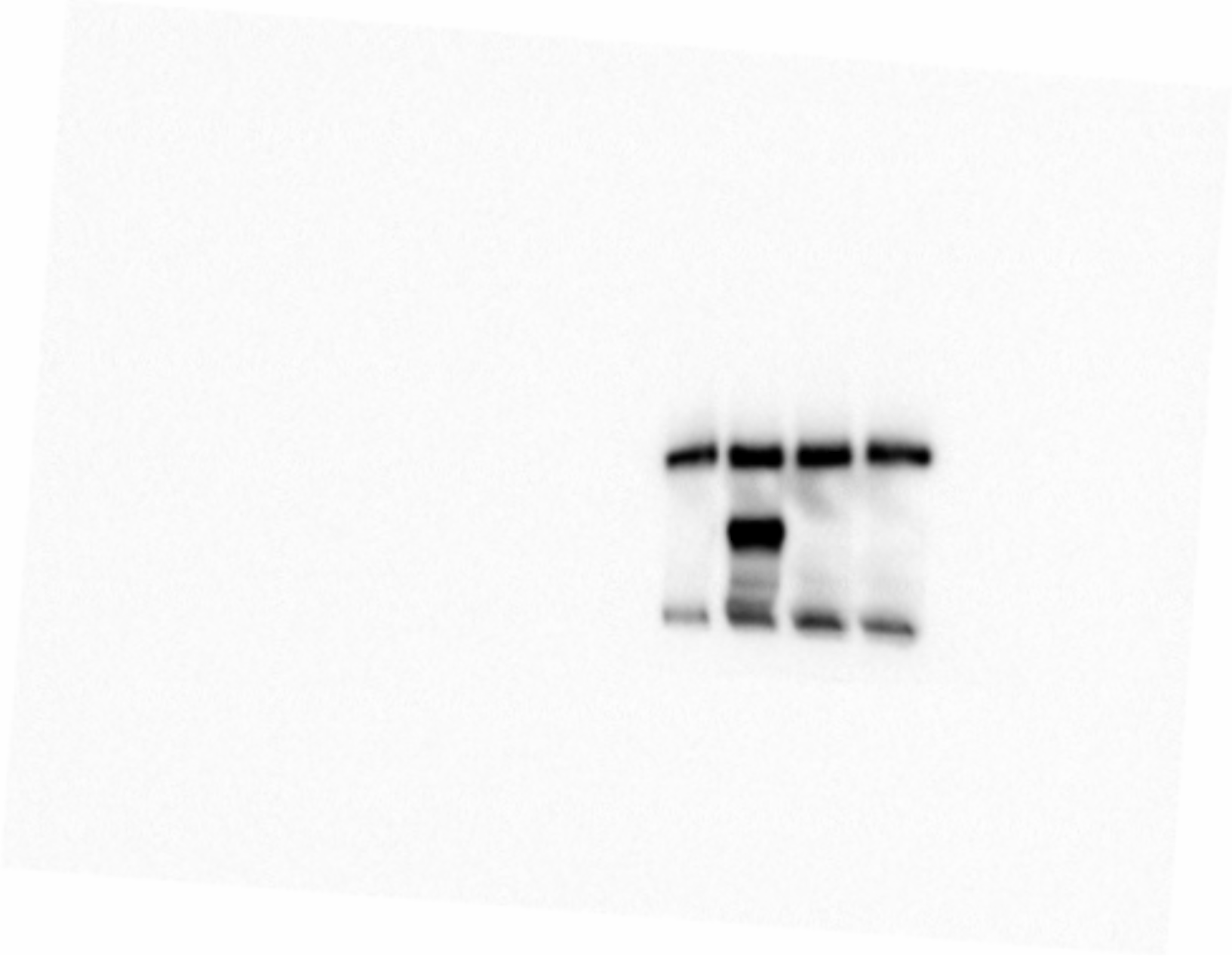

Supplement: Figure 2—source data 2. [file elife-100601-fig2-data2.zip › Figure 2-Source Data 2/Figure2b/IP-WTAP.tif]

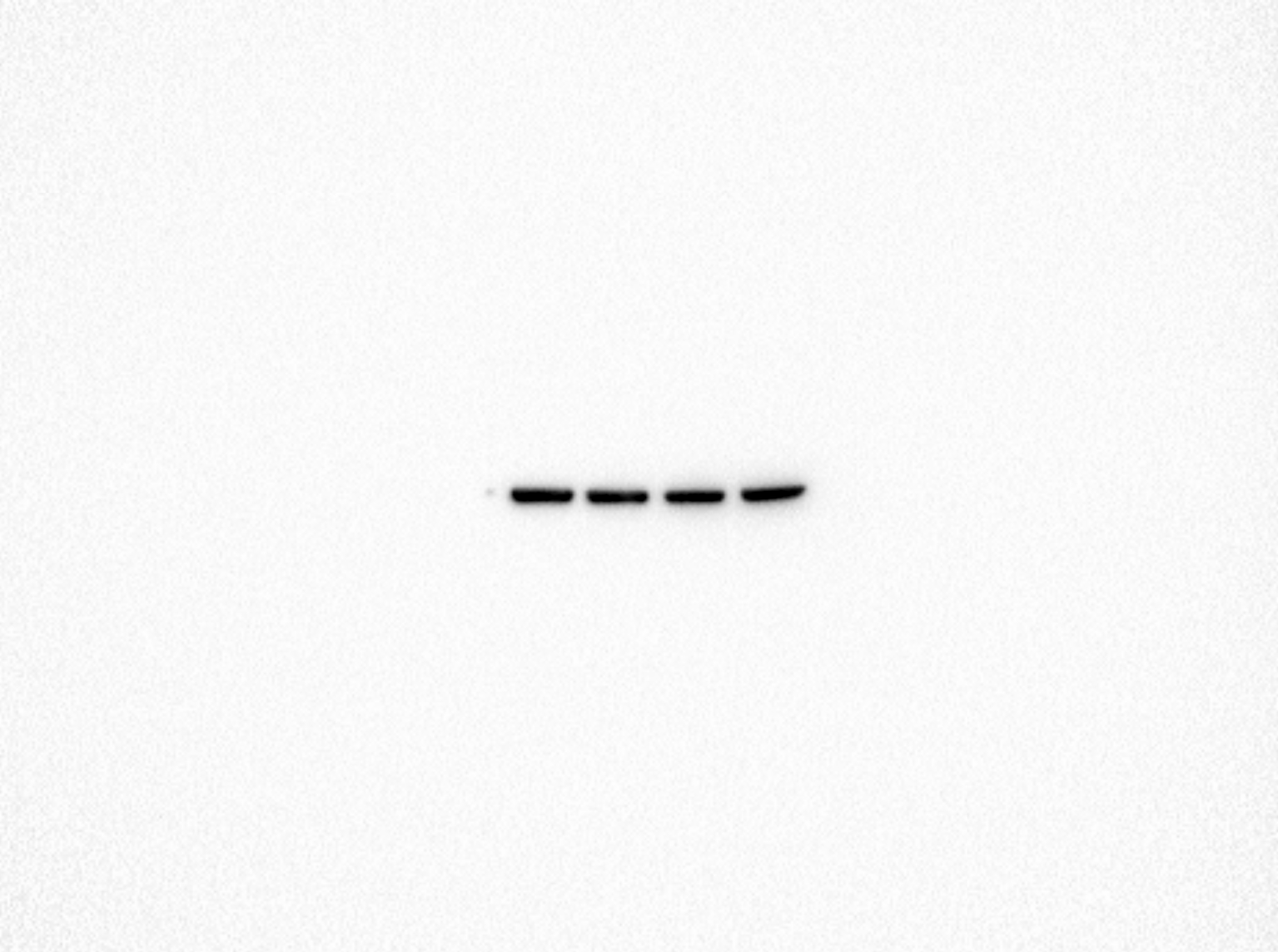

Supplement: Figure 2—source data 2. [file elife-100601-fig2-data2.zip › Figure 2-Source Data 2/Figure2b/actin.tif]

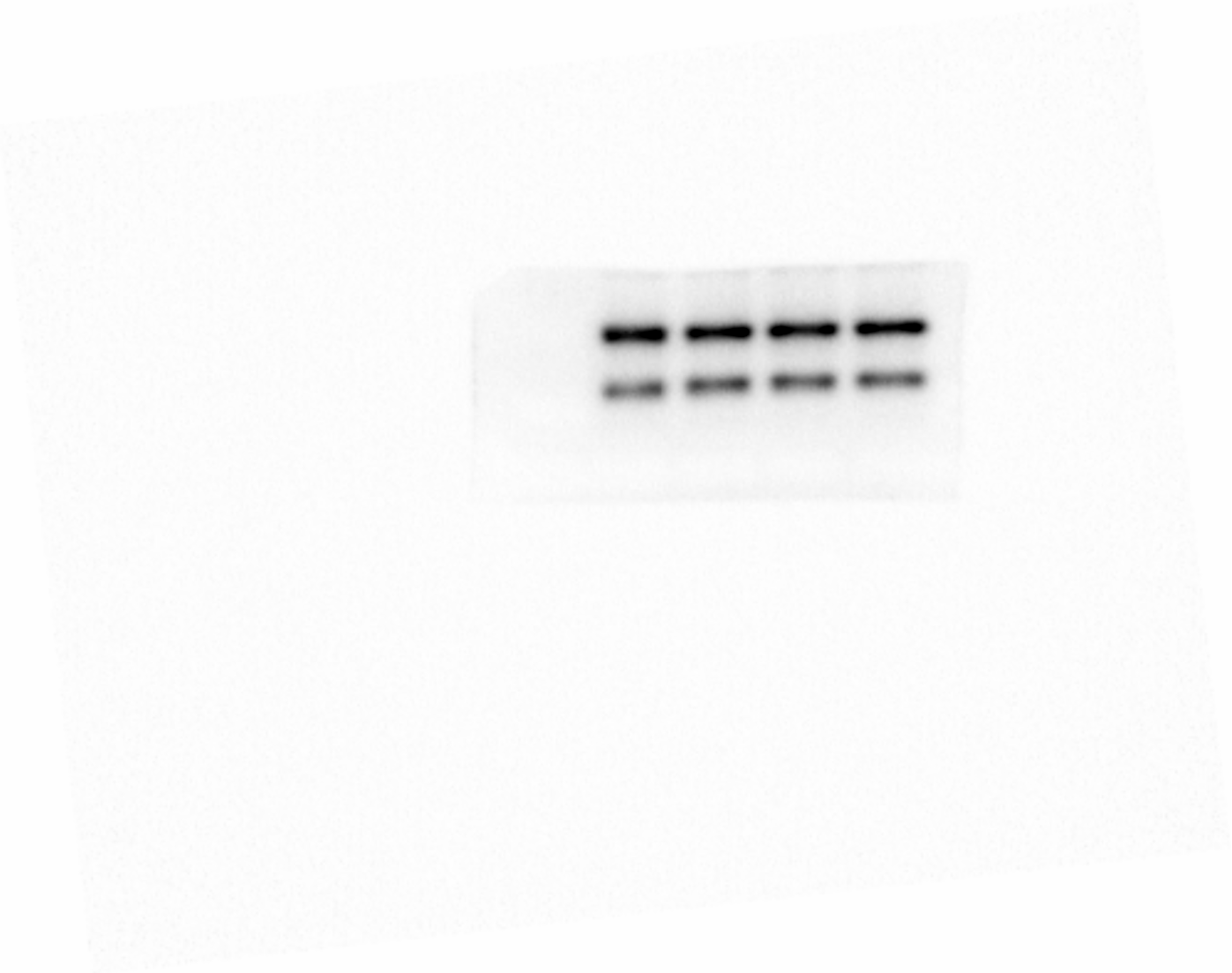

Supplement: Figure 2—source data 2. [file elife-100601-fig2-data2.zip › Figure 2-Source Data 2/Figure2b/PPP4.tif]

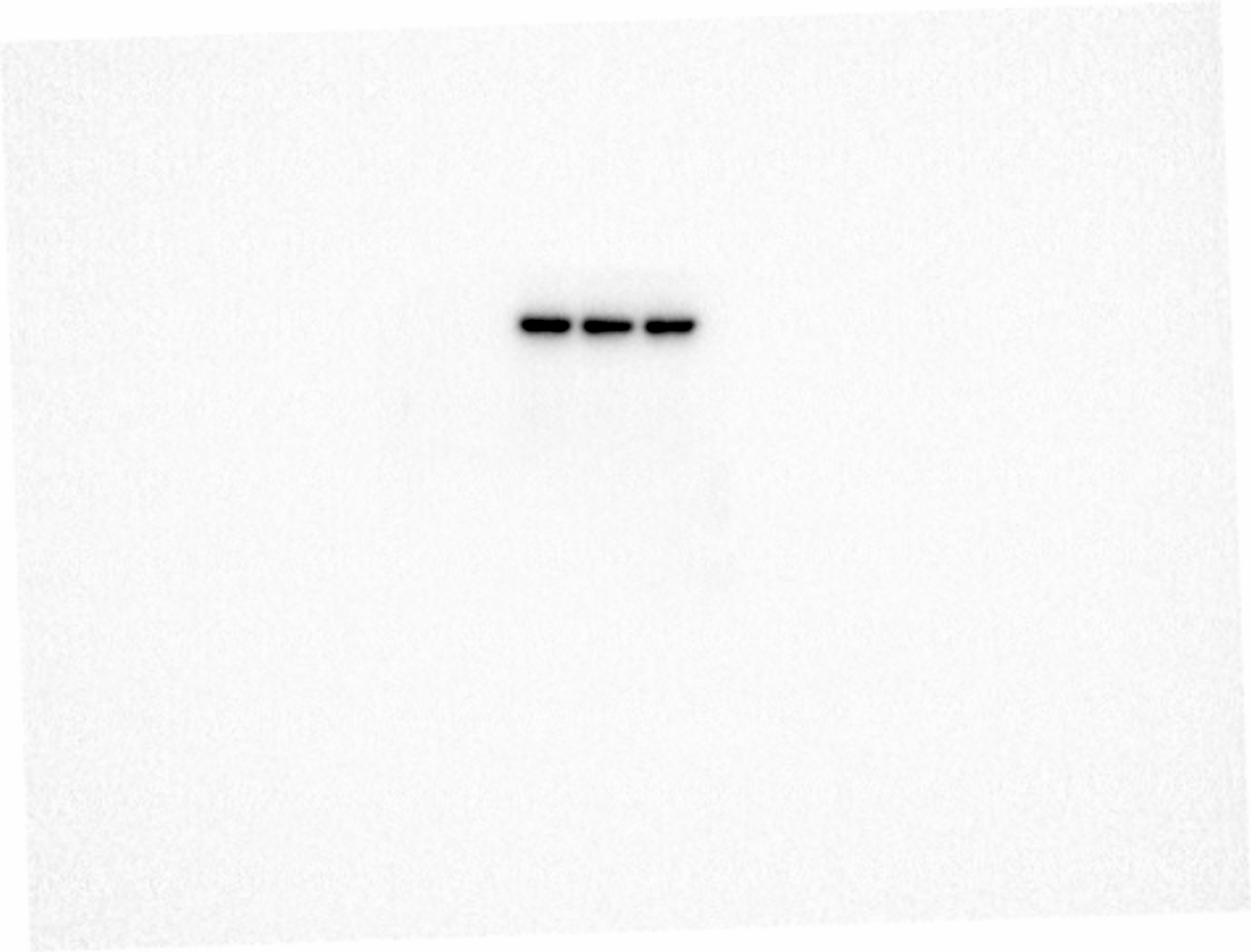

Supplement: Figure 2—source data 2. [file elife-100601-fig2-data2.zip › Figure 2-Source Data 2/Figure2b/WTAP.tif]

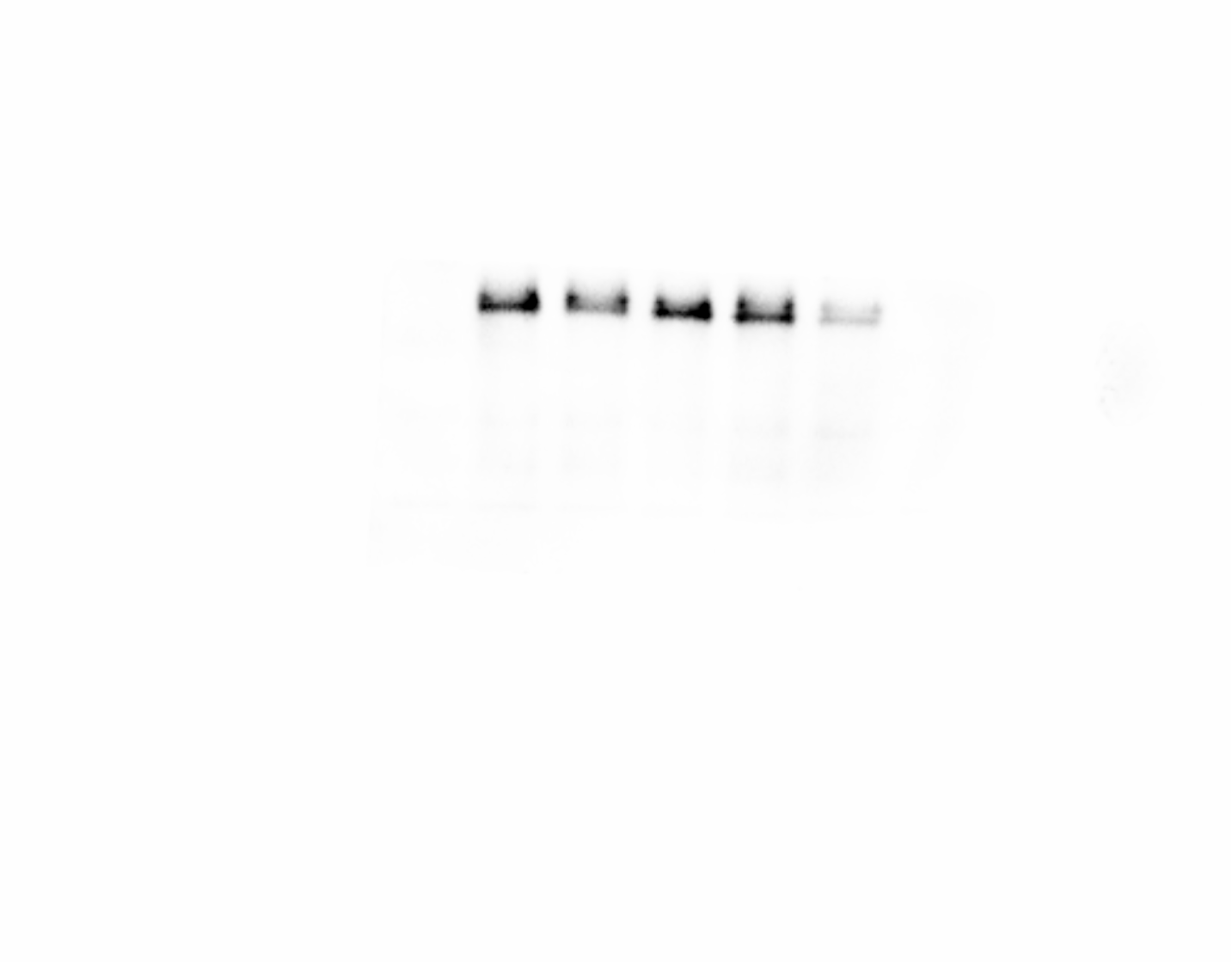

Supplement: Figure 2—source data 2. [file elife-100601-fig2-data2.zip › Figure 2-Source Data 2/Figure2c/IP-pan-p.tif]

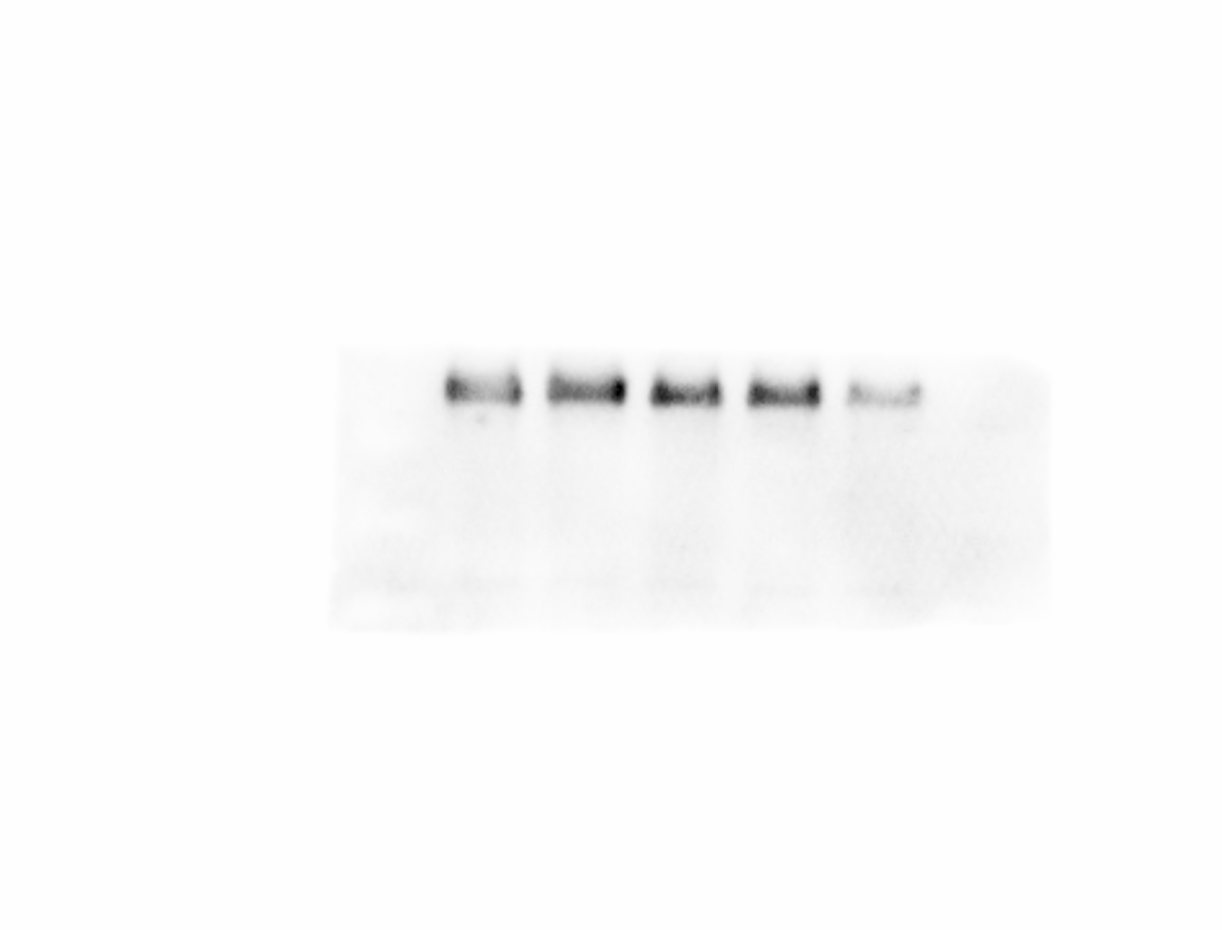

Supplement: Figure 2—source data 2. [file elife-100601-fig2-data2.zip › Figure 2-Source Data 2/Figure2c/IP-WTAP.tif]

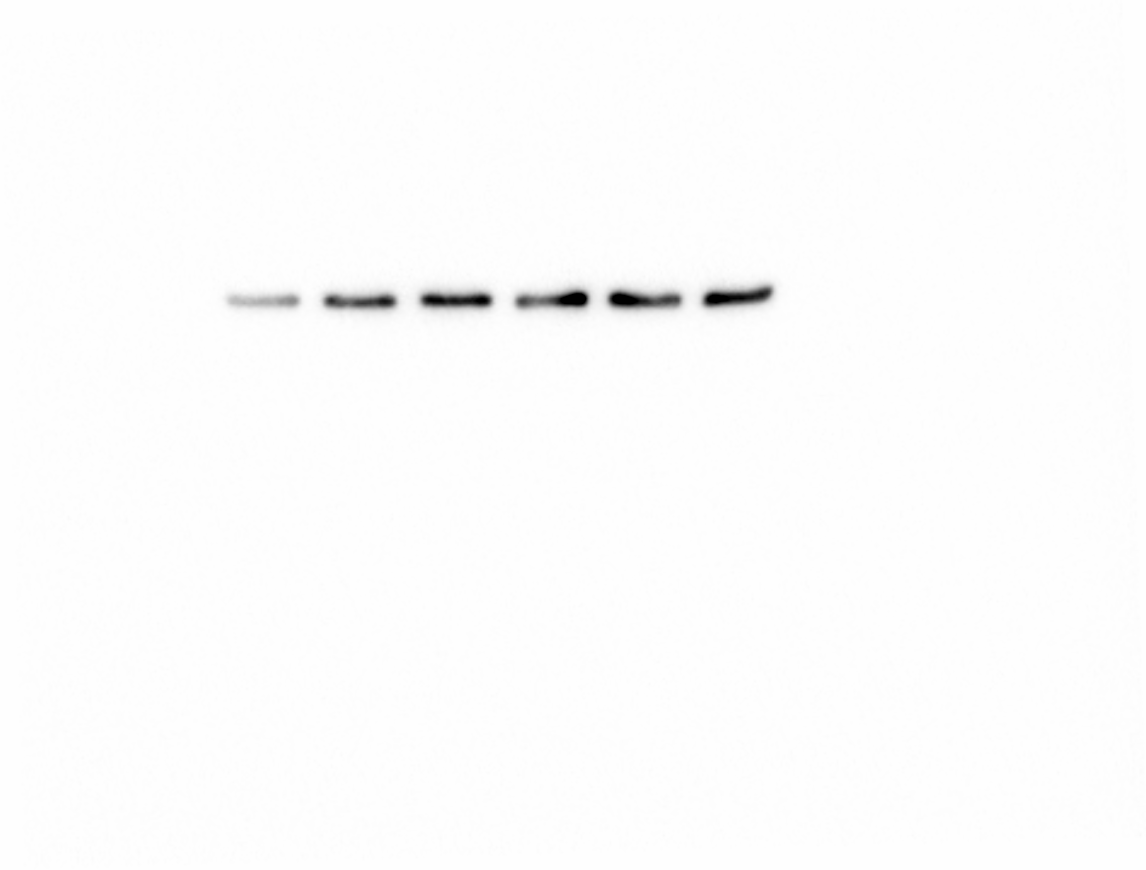

Supplement: Figure 2—source data 2. [file elife-100601-fig2-data2.zip › Figure 2-Source Data 2/Figure2c/actin.tif]

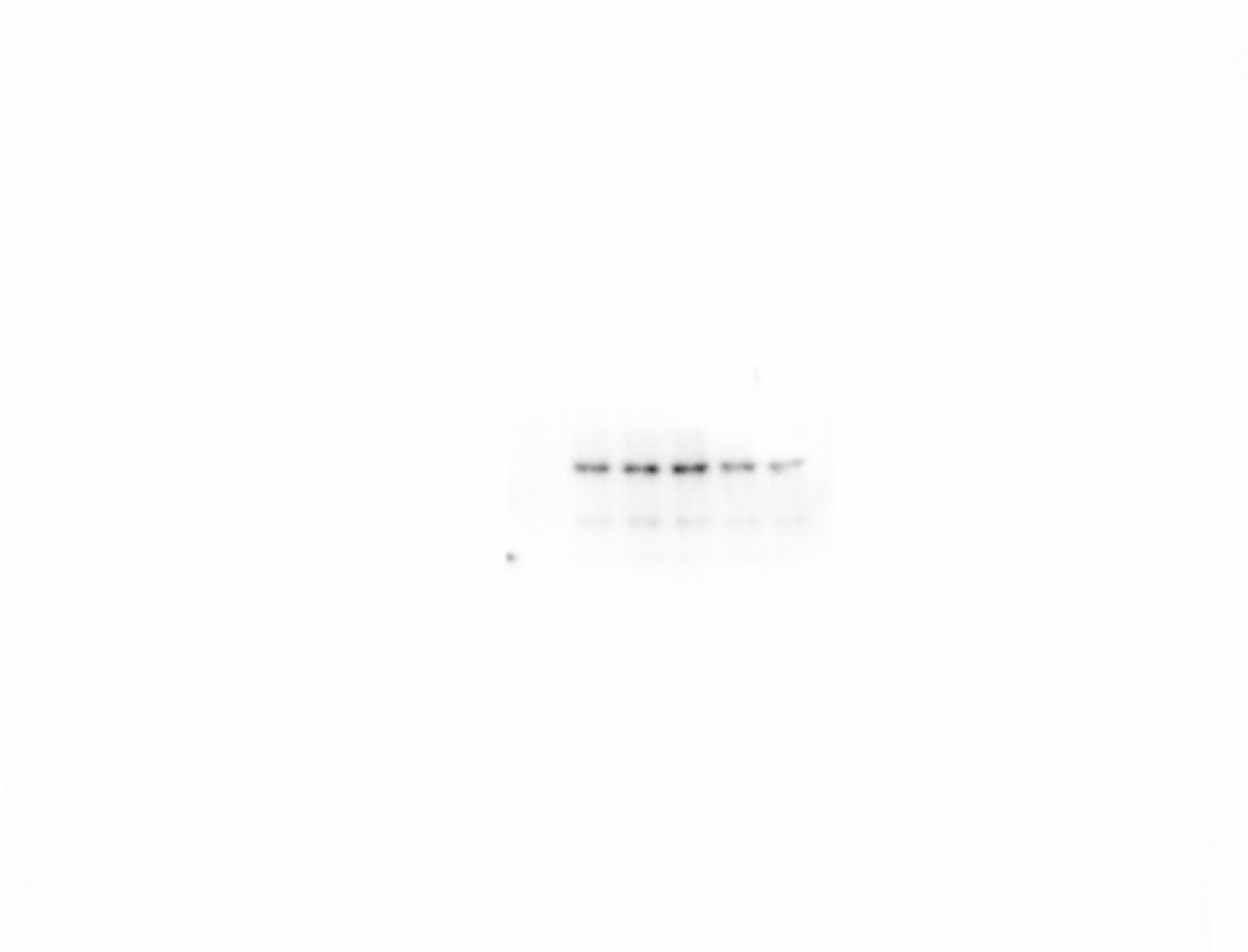

Supplement: Figure 2—source data 2. [file elife-100601-fig2-data2.zip › Figure 2-Source Data 2/Figure2c/PPP4.tif]

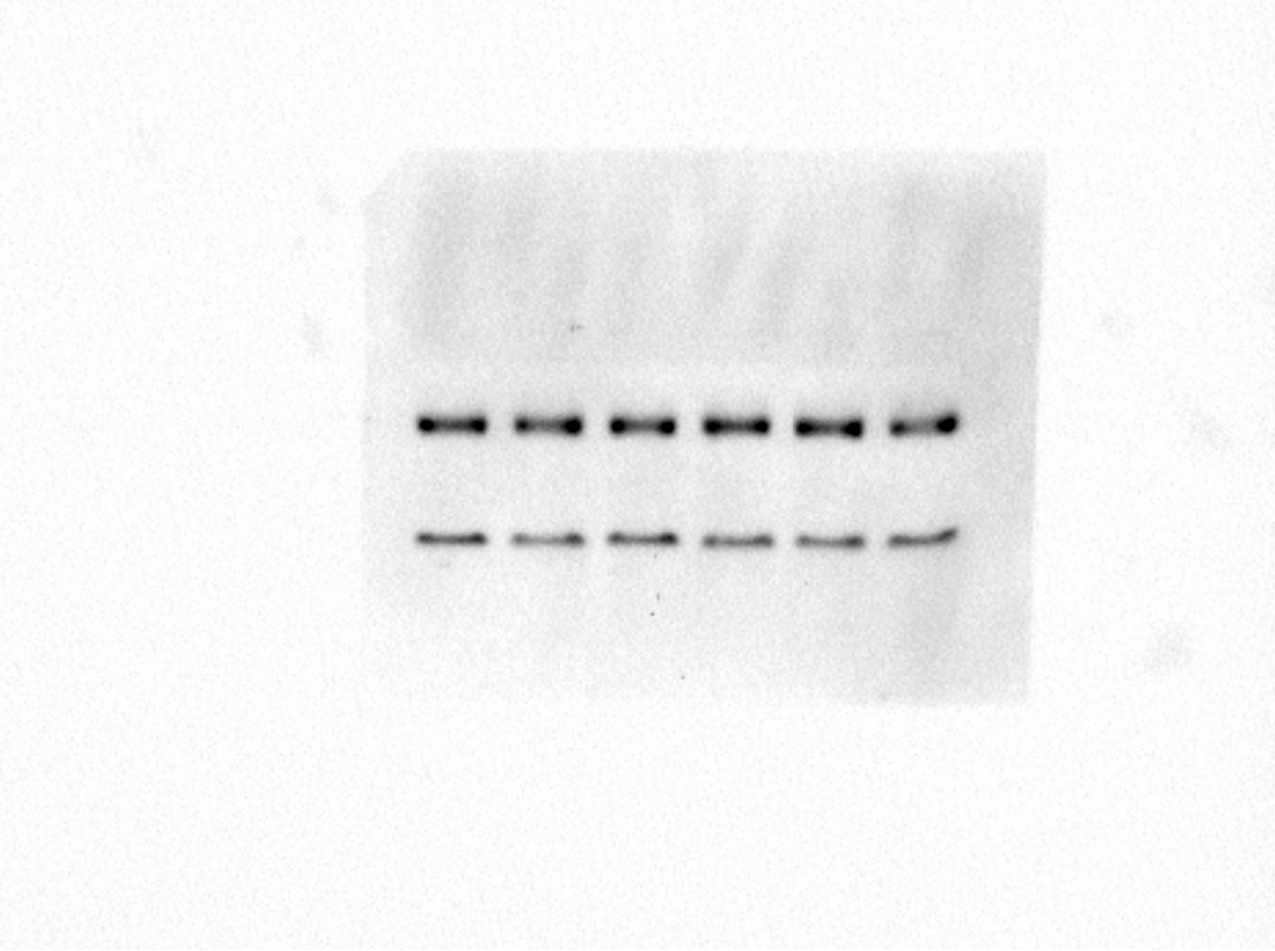

Supplement: Figure 2—source data 2. [file elife-100601-fig2-data2.zip › Figure 2-Source Data 2/Figure2c/WTAP.tif]

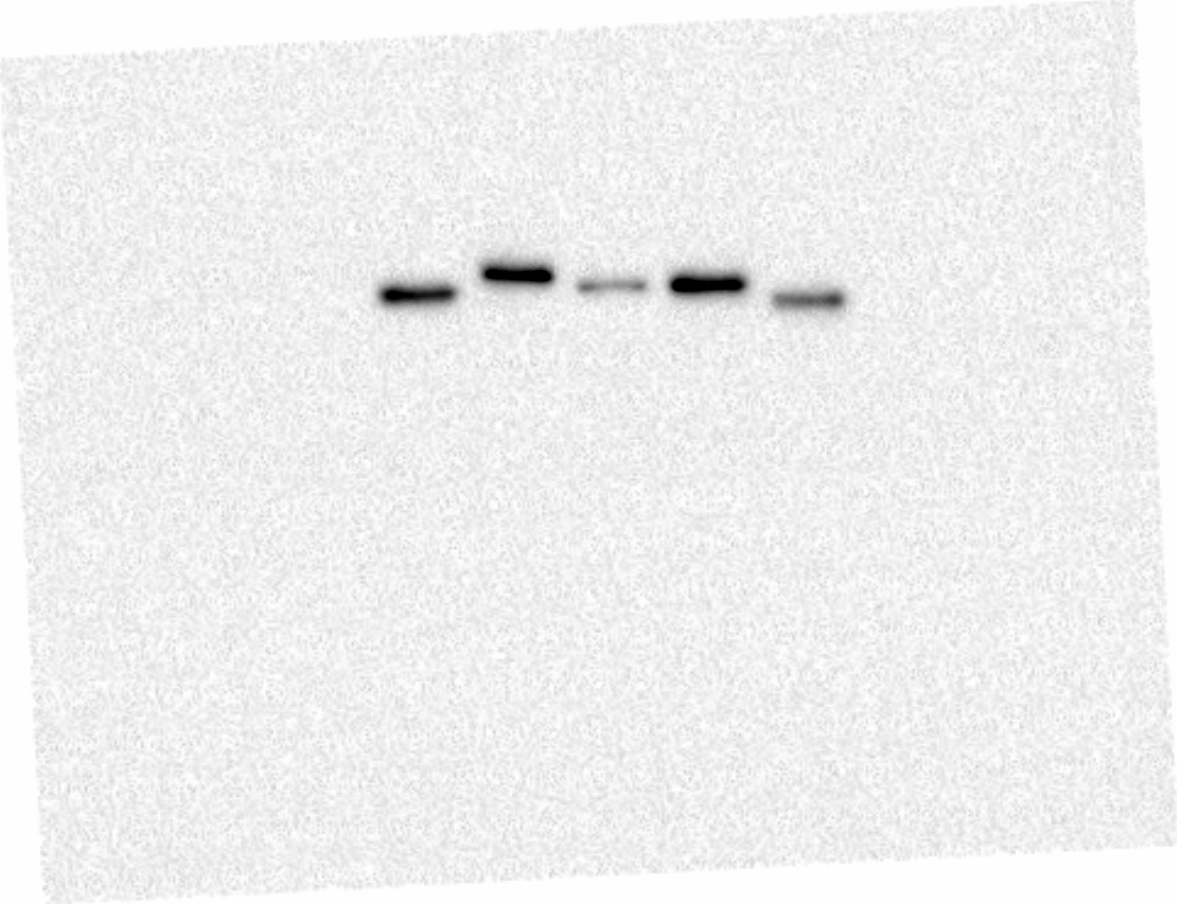

Supplement: Figure 2—figure supplement 1—source data 2. [file elife-100601-fig2-figsupp1-data2.zip › Figure 2-figure supplement1-Source Data 2/Figure2-figure supplement 1a/IP-Flag.tif]

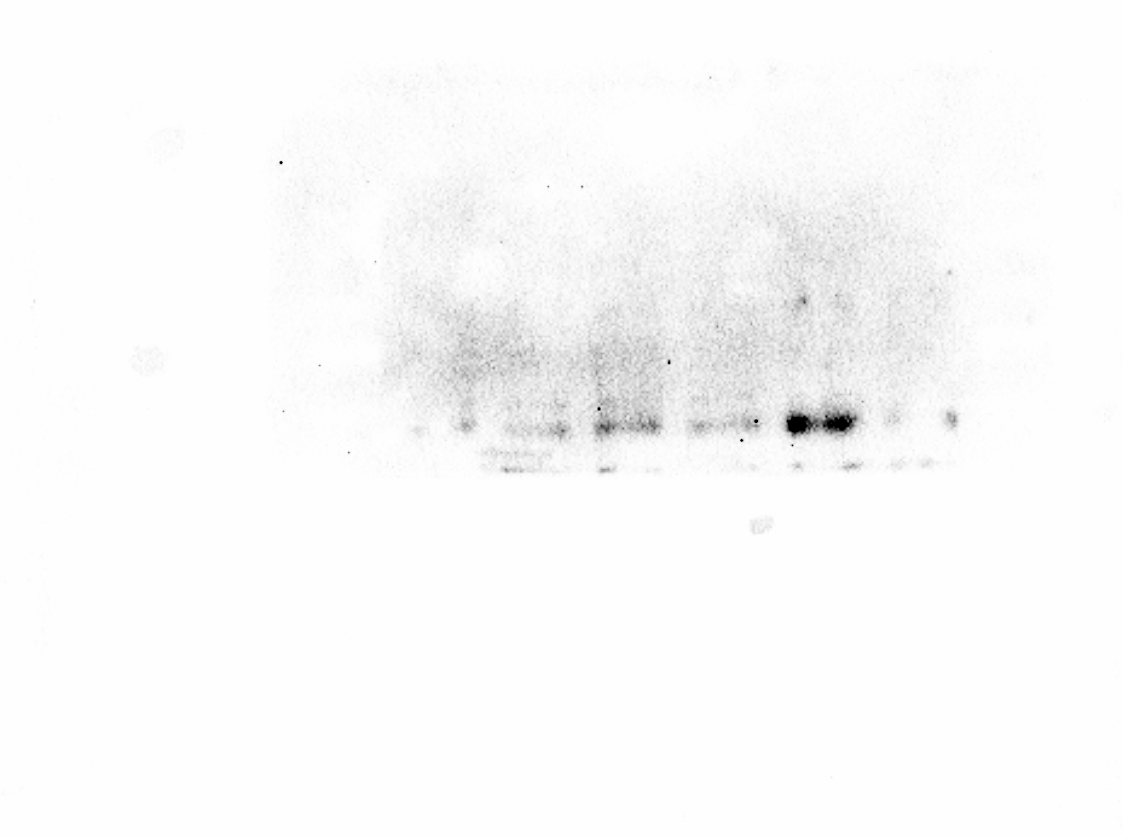

Supplement: Figure 2—figure supplement 1—source data 2. [file elife-100601-fig2-figsupp1-data2.zip › Figure 2-figure supplement1-Source Data 2/Figure2-figure supplement 1a/IP-WTAP.tif]

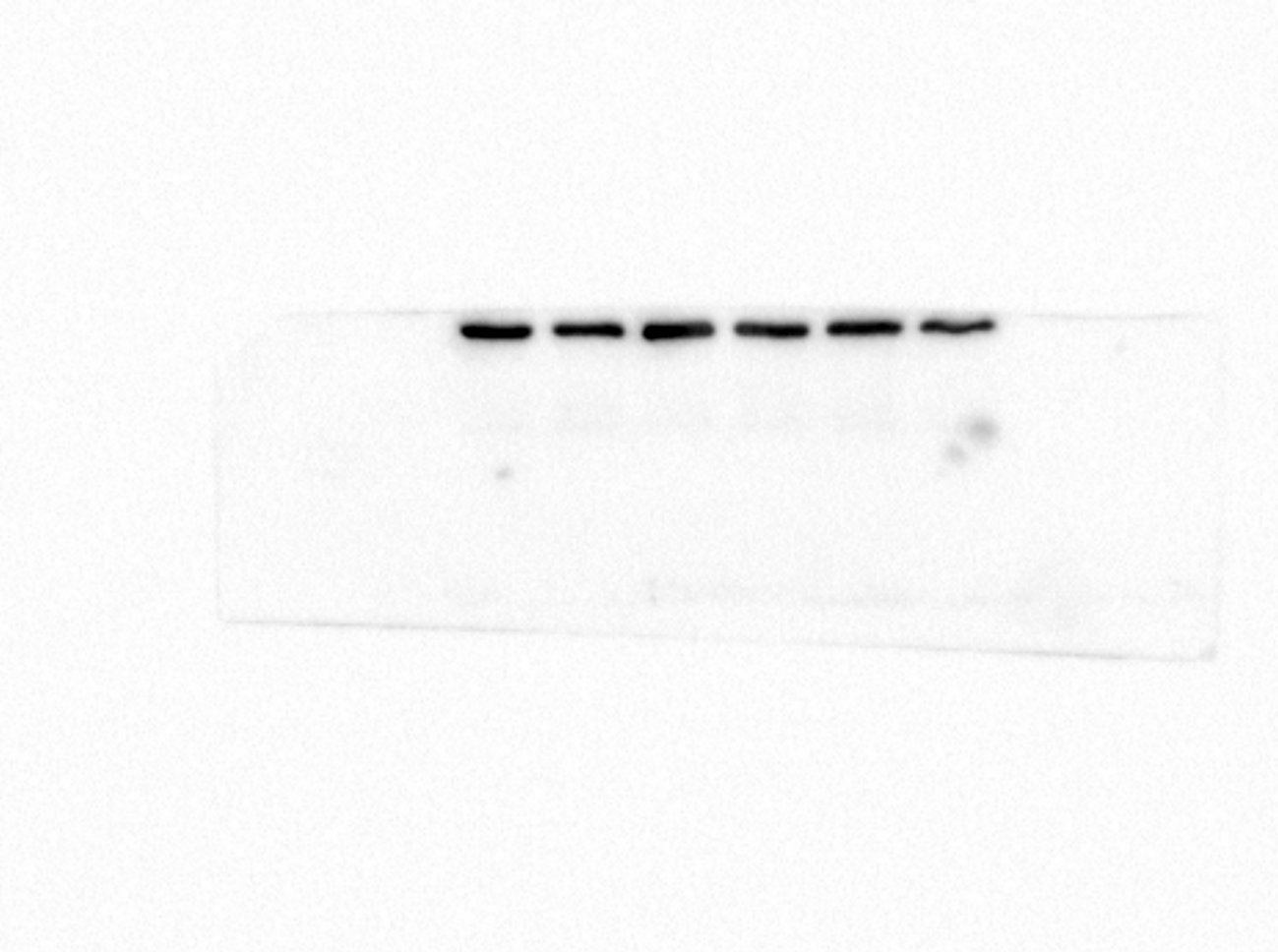

Supplement: Figure 2—figure supplement 1—source data 2. [file elife-100601-fig2-figsupp1-data2.zip › Figure 2-figure supplement1-Source Data 2/Figure2-figure supplement 1a/actin.tif]

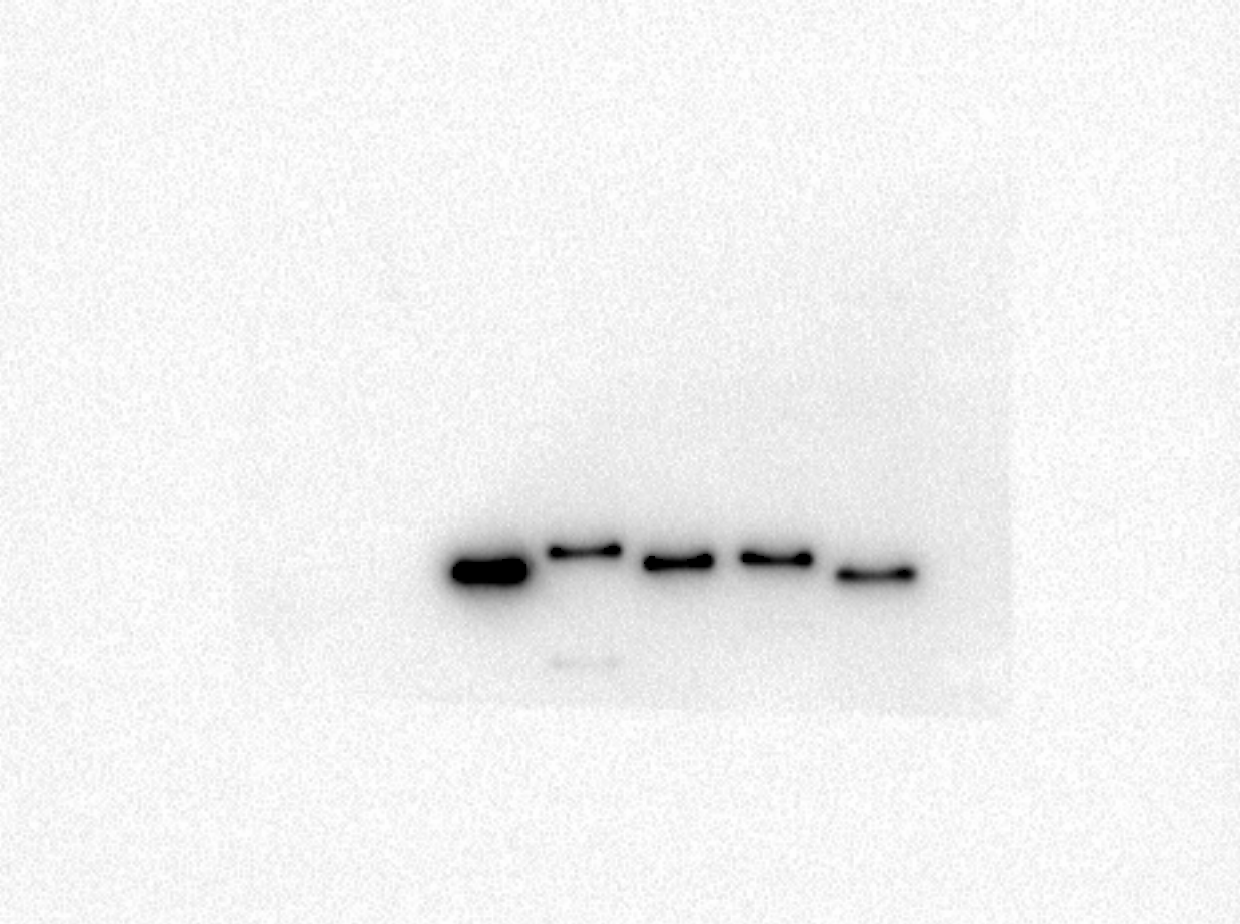

Supplement: Figure 2—figure supplement 1—source data 2. [file elife-100601-fig2-figsupp1-data2.zip › Figure 2-figure supplement1-Source Data 2/Figure2-figure supplement 1a/WCL-Flag.tif]

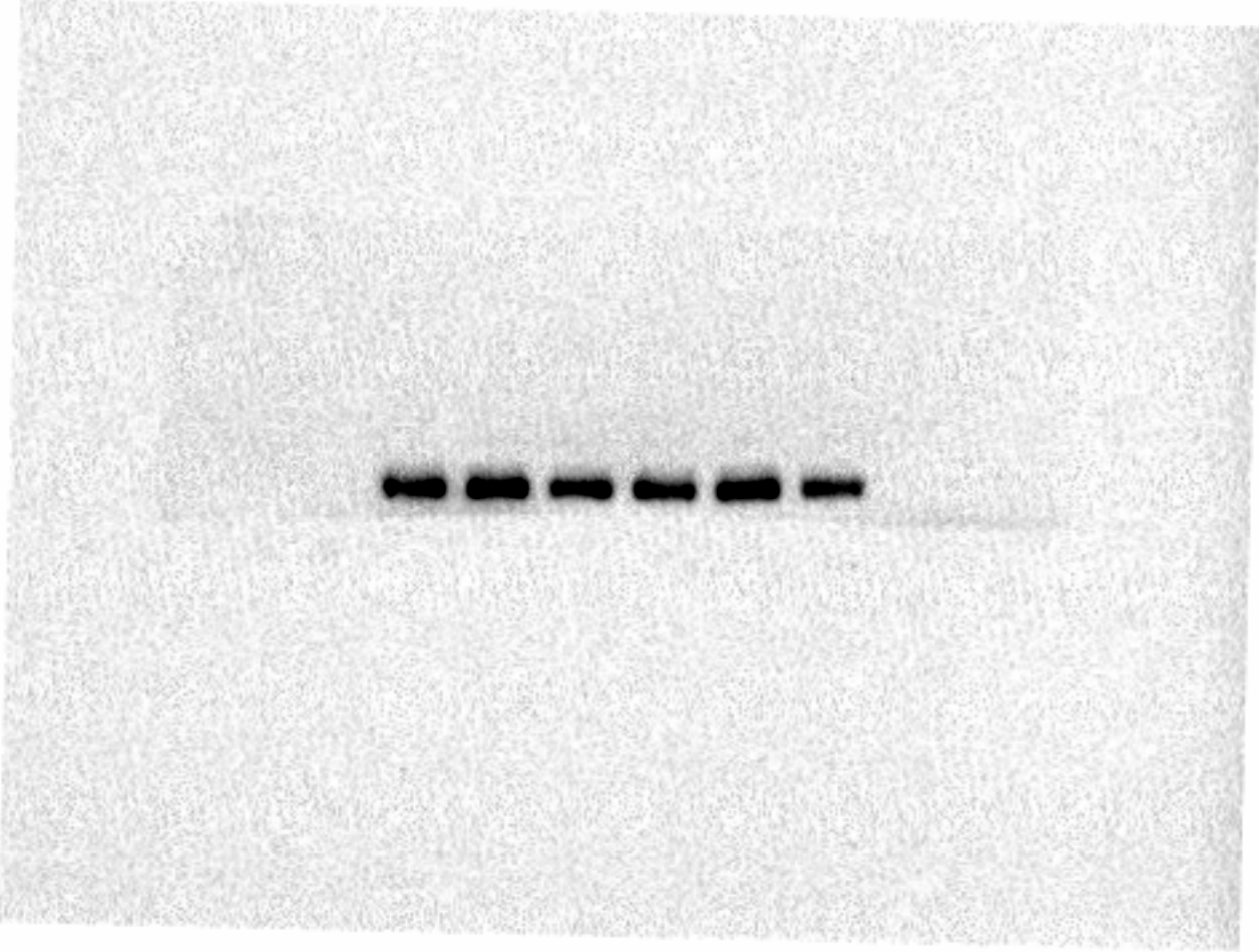

Supplement: Figure 2—figure supplement 1—source data 2. [file elife-100601-fig2-figsupp1-data2.zip › Figure 2-figure supplement1-Source Data 2/Figure2-figure supplement 1a/WCL WTAP.tif]

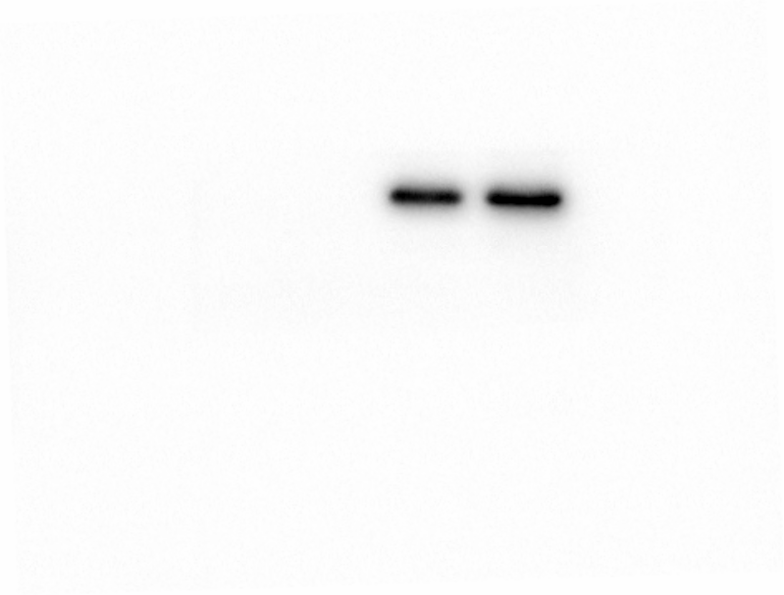

Supplement: Figure 2—figure supplement 1—source data 2. [file elife-100601-fig2-figsupp1-data2.zip › Figure 2-figure supplement1-Source Data 2/Figure2-figure supplement 1b/IP-Flag.tif]

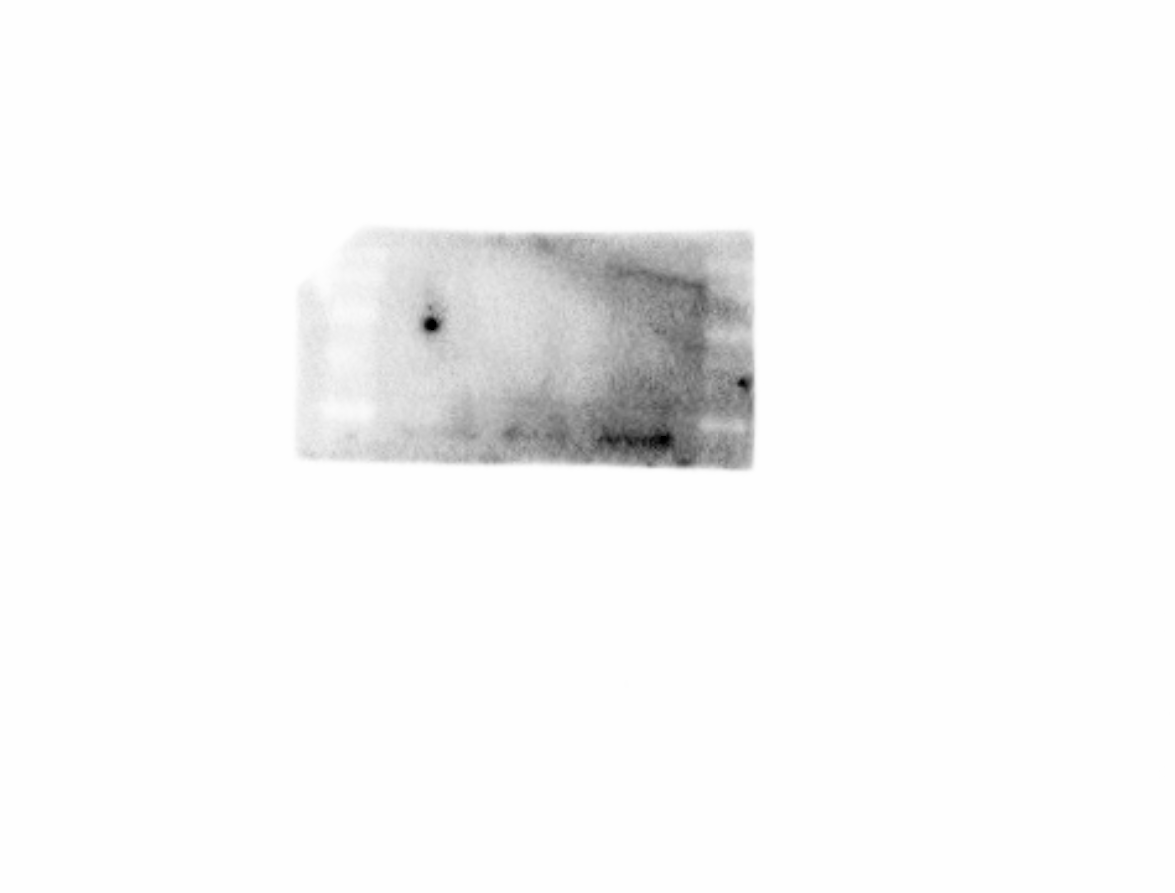

Supplement: Figure 2—figure supplement 1—source data 2. [file elife-100601-fig2-figsupp1-data2.zip › Figure 2-figure supplement1-Source Data 2/Figure2-figure supplement 1b/IP-WTAP.tif]

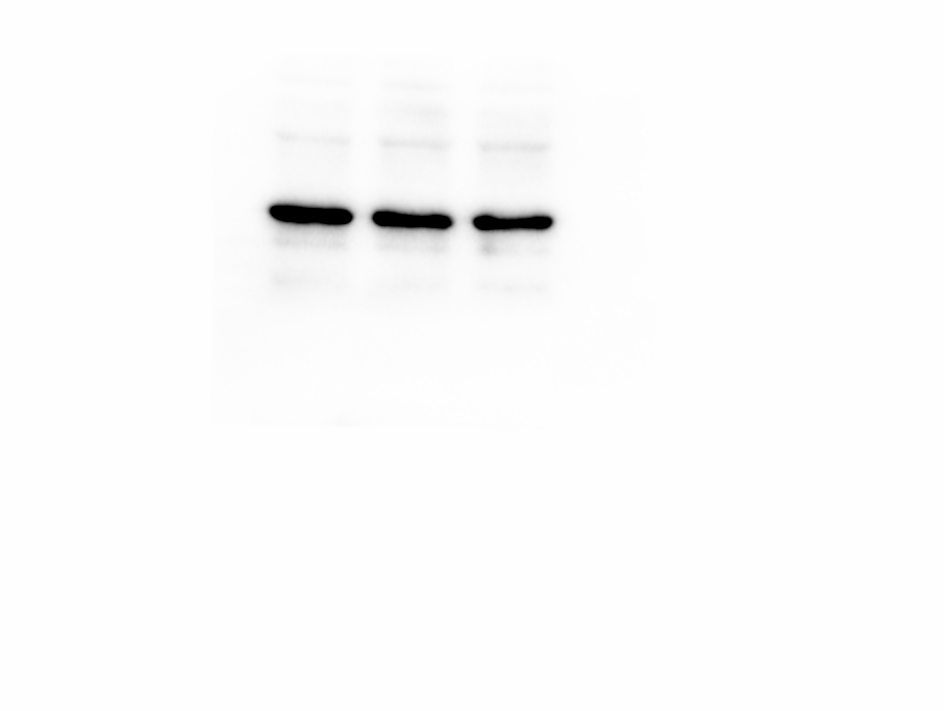

Supplement: Figure 2—figure supplement 1—source data 2. [file elife-100601-fig2-figsupp1-data2.zip › Figure 2-figure supplement1-Source Data 2/Figure2-figure supplement 1b/actin.tif]

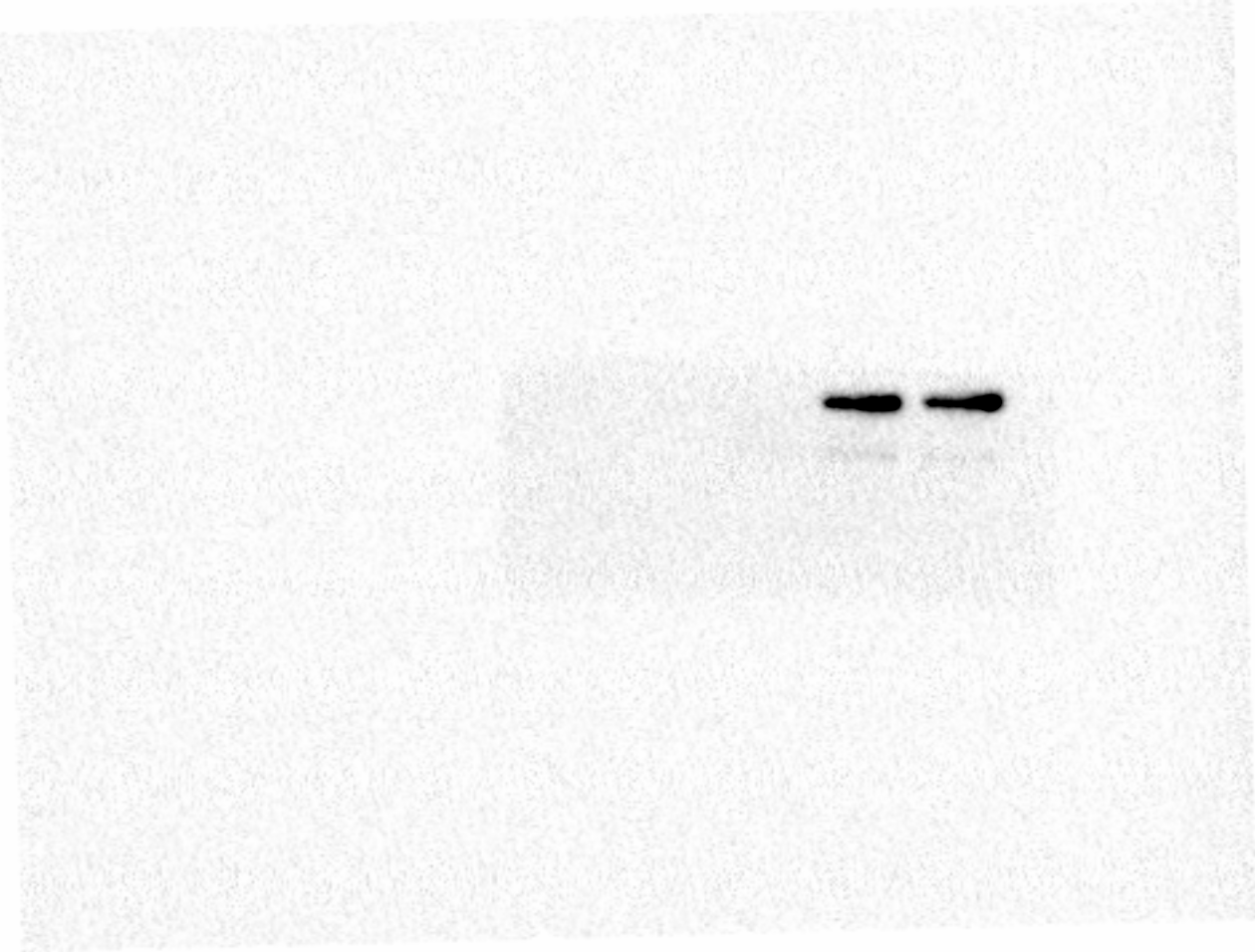

Supplement: Figure 2—figure supplement 1—source data 2. [file elife-100601-fig2-figsupp1-data2.zip › Figure 2-figure supplement1-Source Data 2/Figure2-figure supplement 1b/Flag.tif]

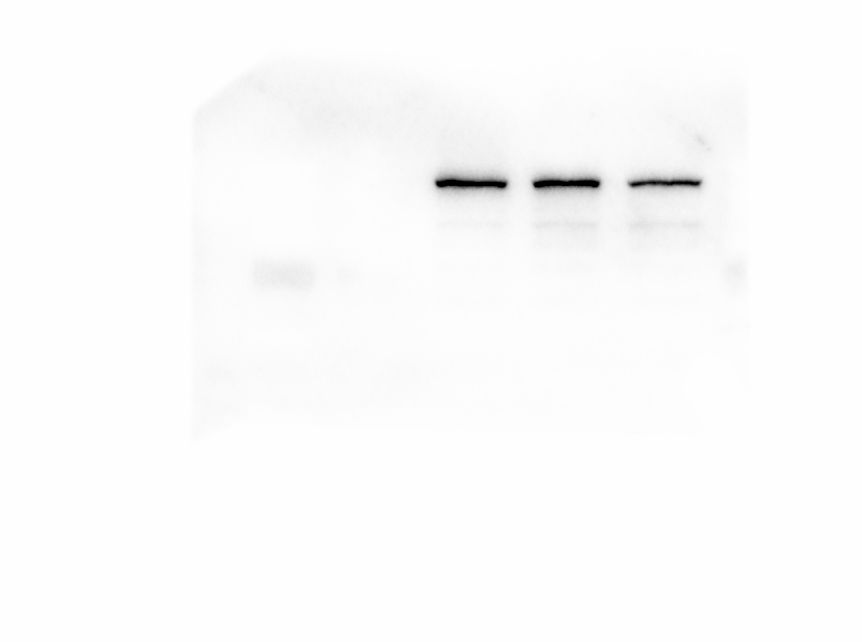

Supplement: Figure 2—figure supplement 1—source data 2. [file elife-100601-fig2-figsupp1-data2.zip › Figure 2-figure supplement1-Source Data 2/Figure2-figure supplement 1b/WTAP.tif]

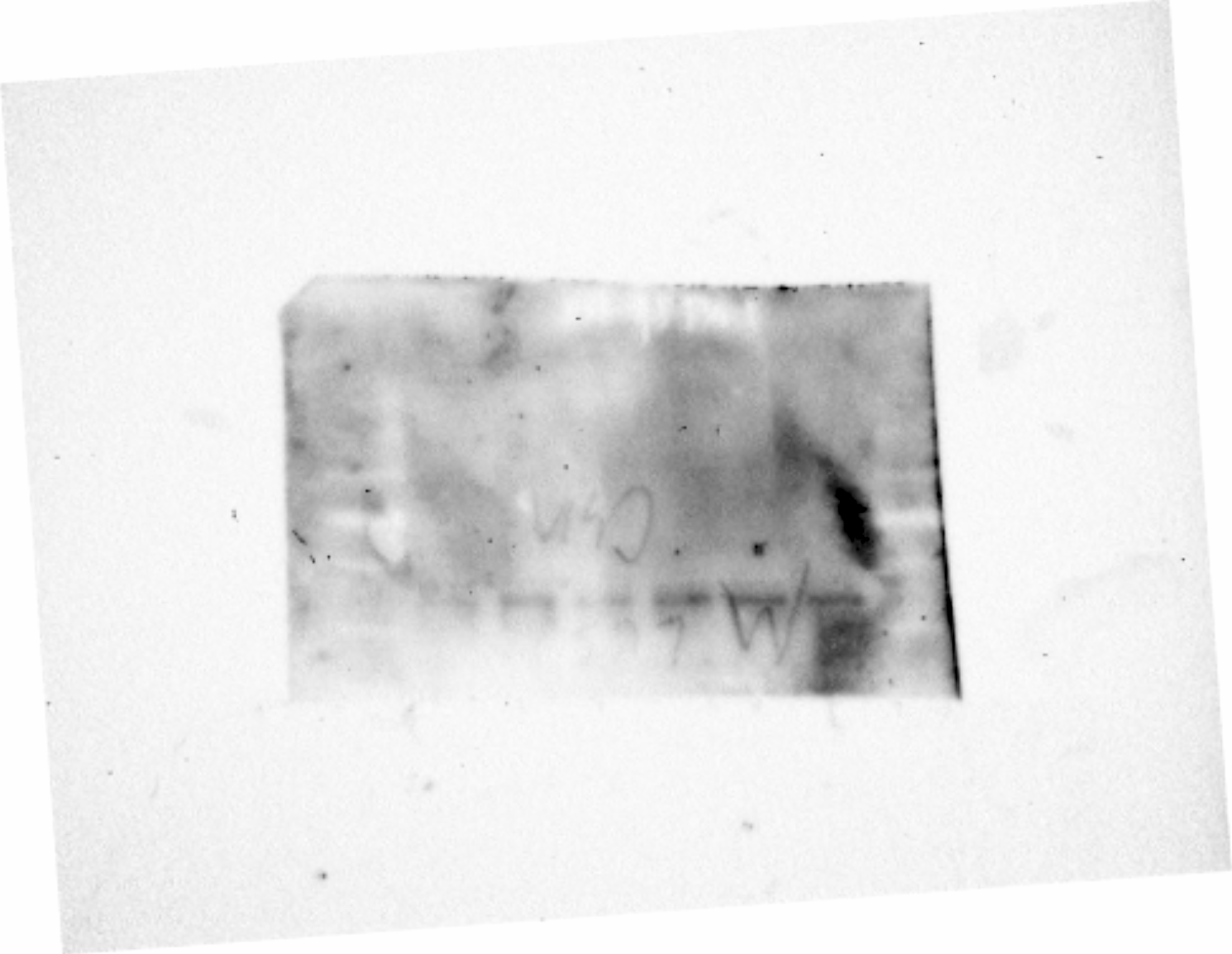

Supplement: Figure 2—figure supplement 1—source data 2. [file elife-100601-fig2-figsupp1-data2.zip › Figure 2-figure supplement1-Source Data 2/Figure2-figure supplement 1c/IP-pan-p.tif]

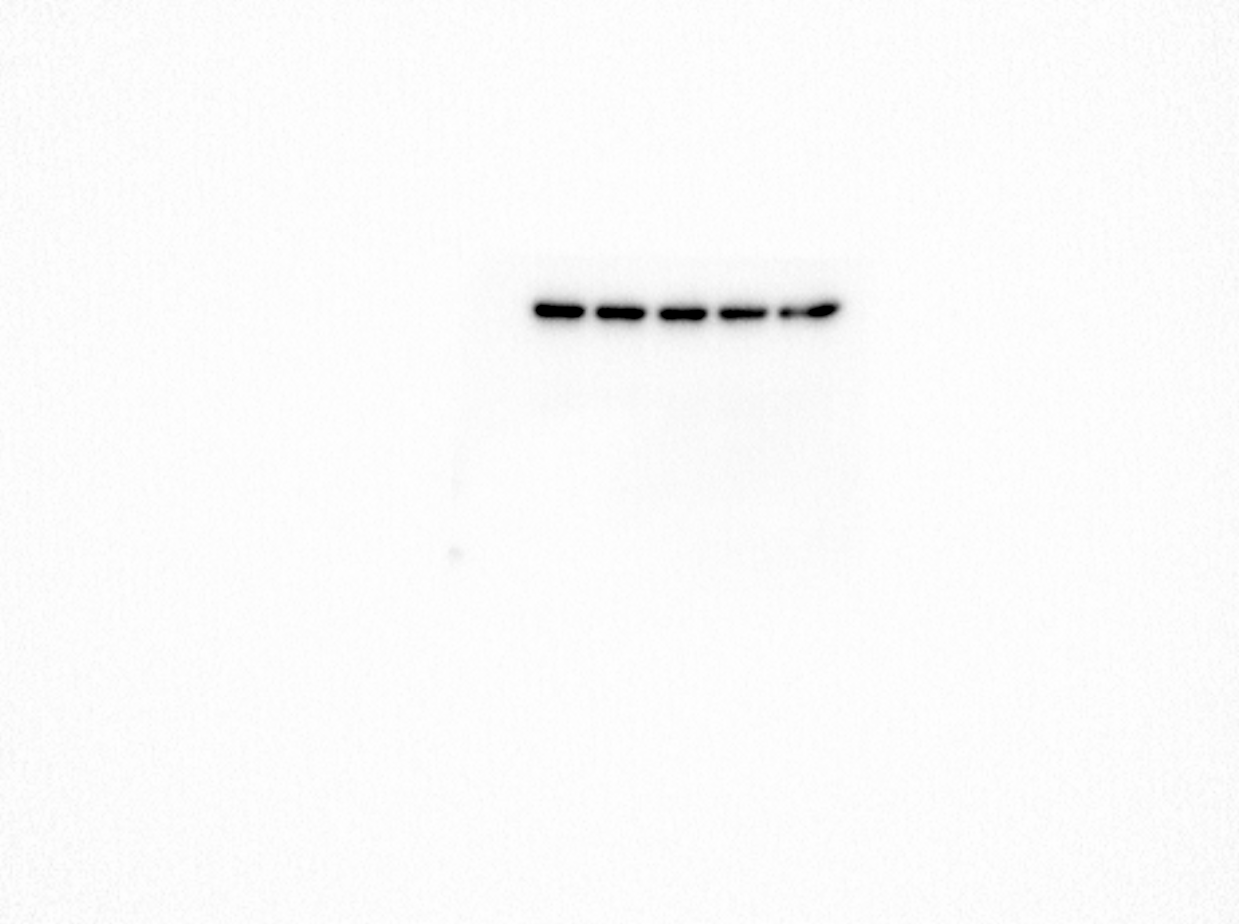

Supplement: Figure 2—figure supplement 1—source data 2. [file elife-100601-fig2-figsupp1-data2.zip › Figure 2-figure supplement1-Source Data 2/Figure2-figure supplement 1c/IP-WTAP.tif]

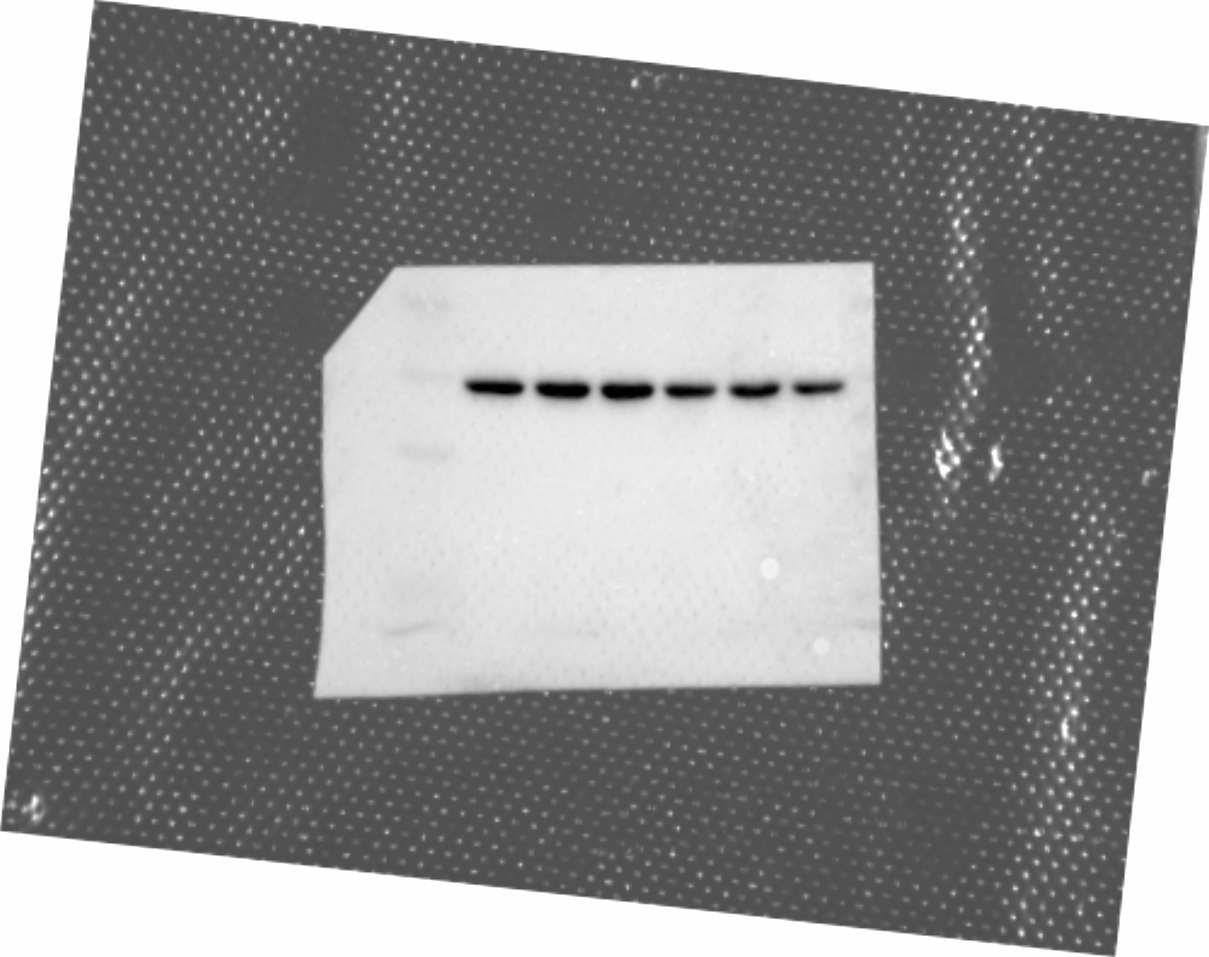

Supplement: Figure 2—figure supplement 1—source data 2. [file elife-100601-fig2-figsupp1-data2.zip › Figure 2-figure supplement1-Source Data 2/Figure2-figure supplement 1c/actin.tif]

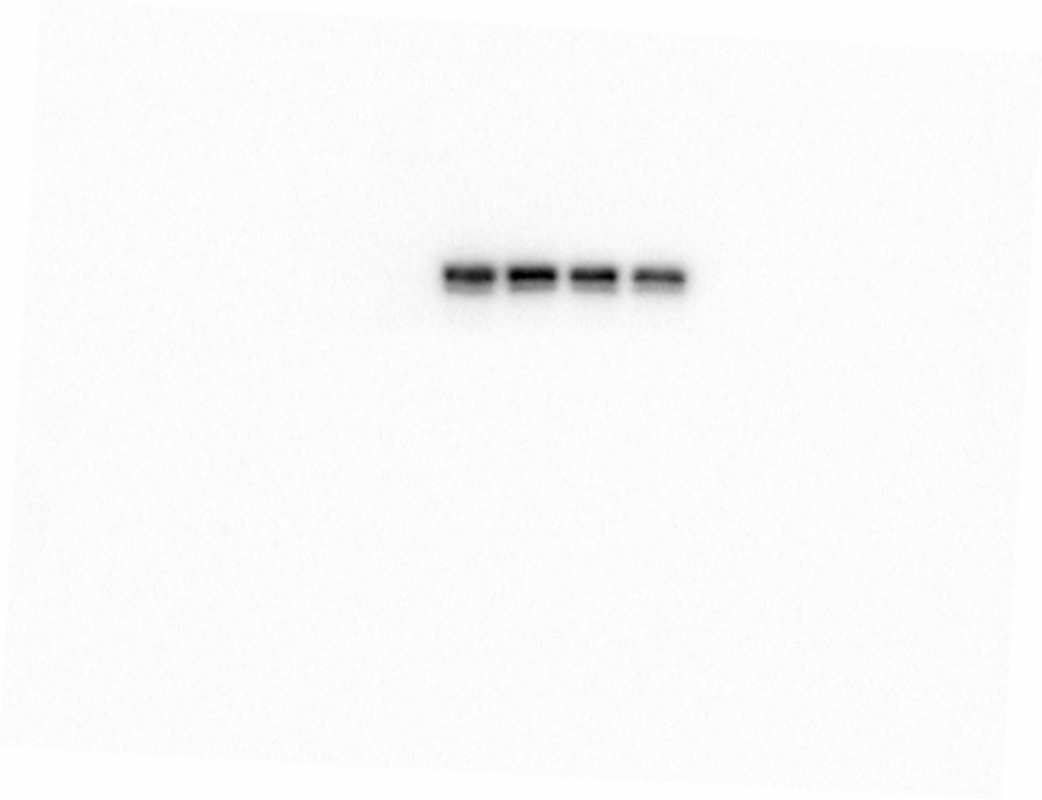

Supplement: Figure 2—figure supplement 1—source data 2. [file elife-100601-fig2-figsupp1-data2.zip › Figure 2-figure supplement1-Source Data 2/Figure2-figure supplement 1c/pSTAT1.tif]

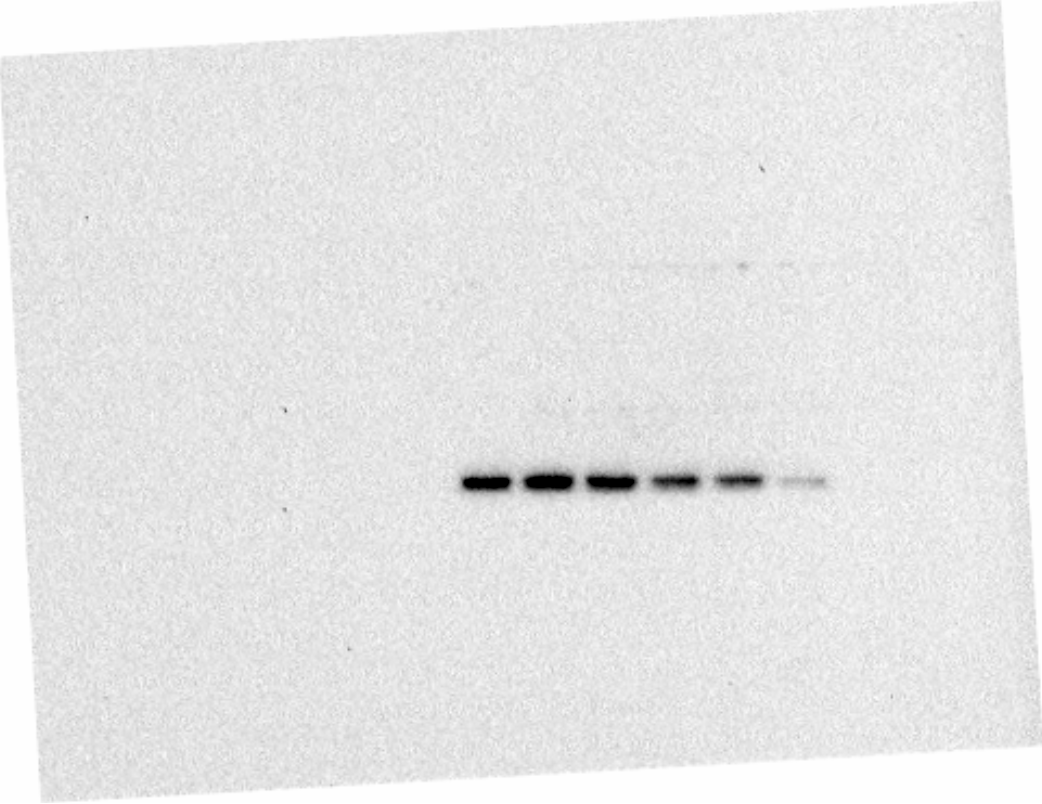

Supplement: Figure 2—figure supplement 1—source data 2. [file elife-100601-fig2-figsupp1-data2.zip › Figure 2-figure supplement1-Source Data 2/Figure2-figure supplement 1c/STAT1.tif]

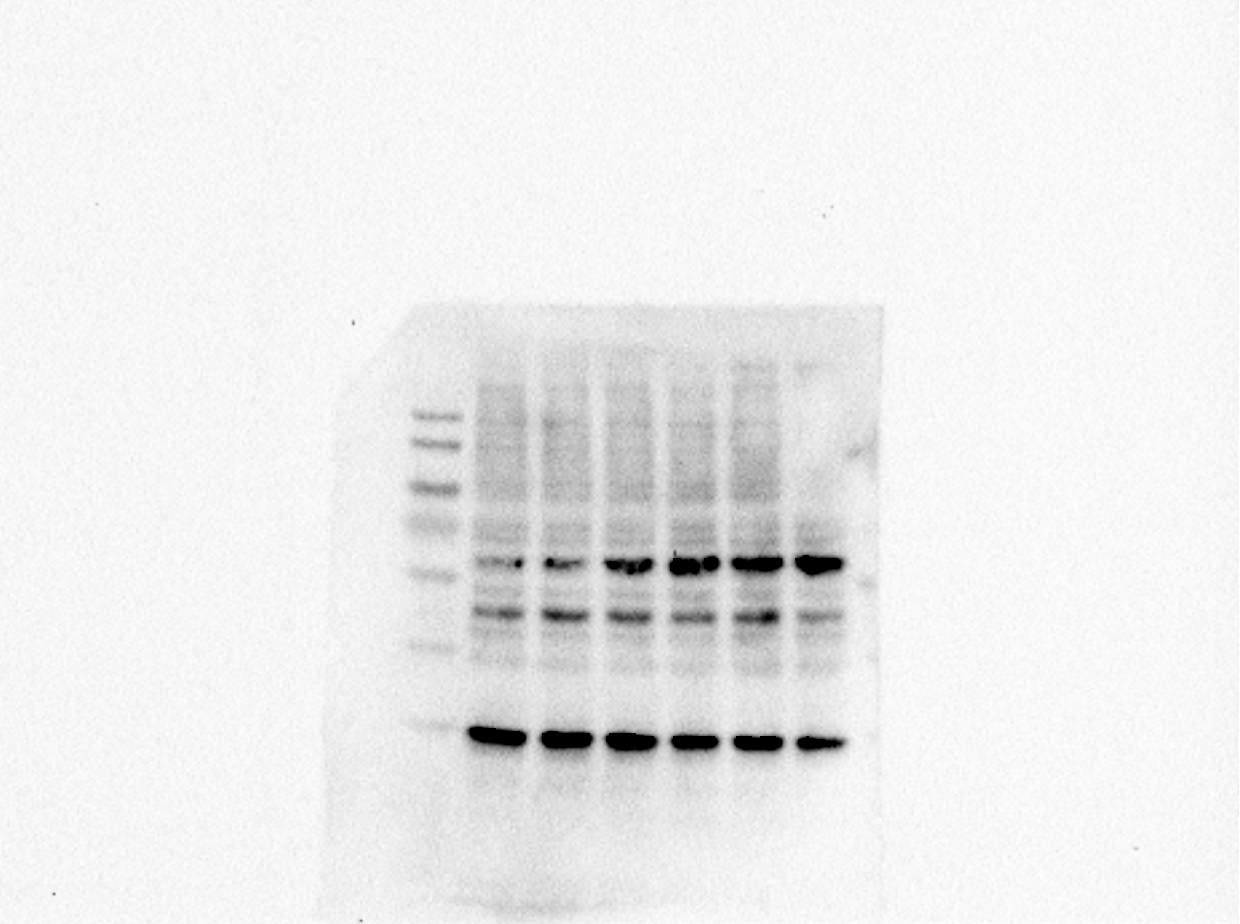

Supplement: Figure 2—figure supplement 1—source data 2. [file elife-100601-fig2-figsupp1-data2.zip › Figure 2-figure supplement1-Source Data 2/Figure2-figure supplement 1c/WTAP.tif]

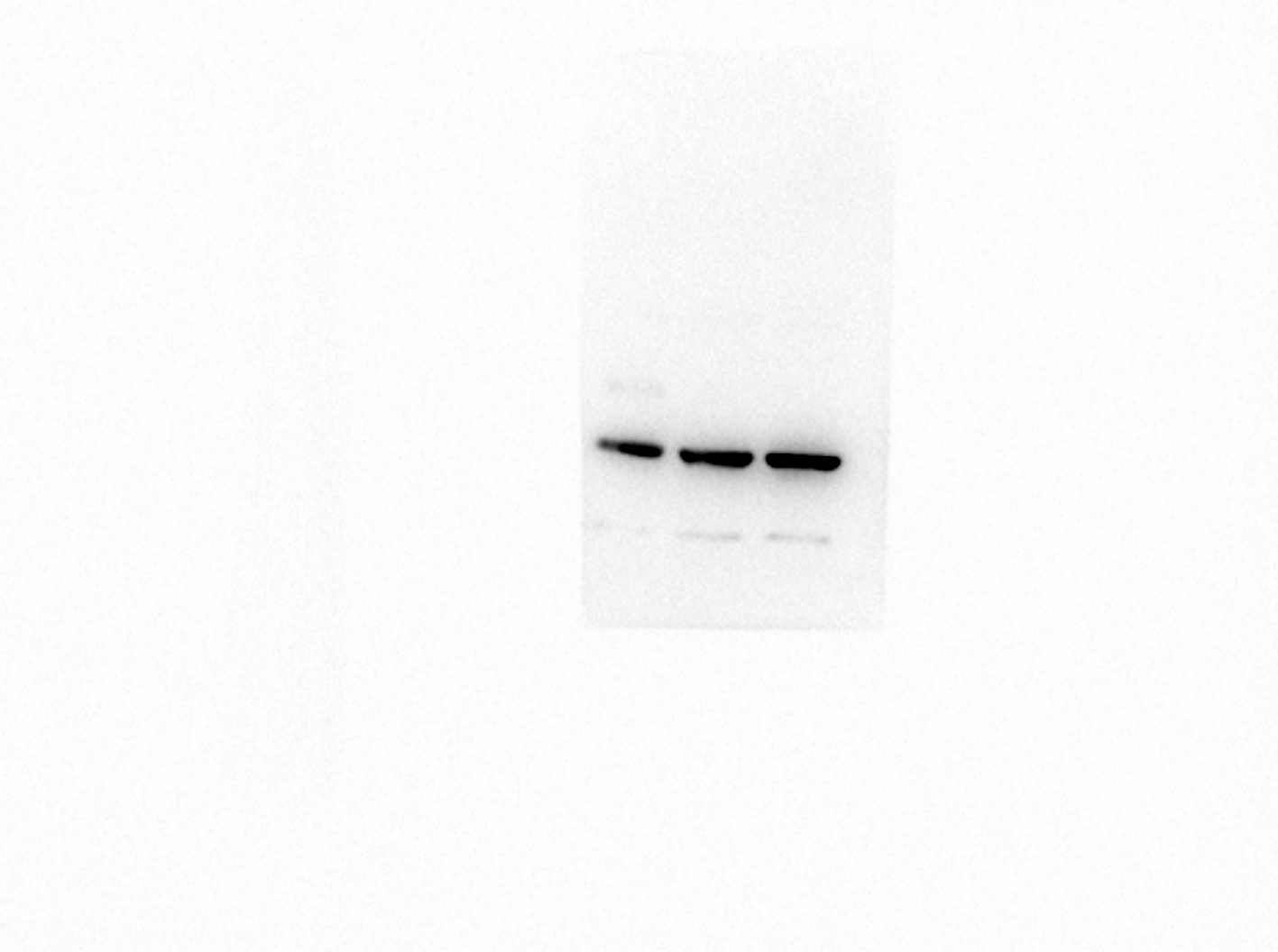

Supplement: Figure 3—figure supplement 1—source data 2. [file elife-100601-fig3-figsupp1-data2.zip › Figure 3-figure supplement 1-Source Data 2/Figure3-figure supplement 1a/actin.tif]

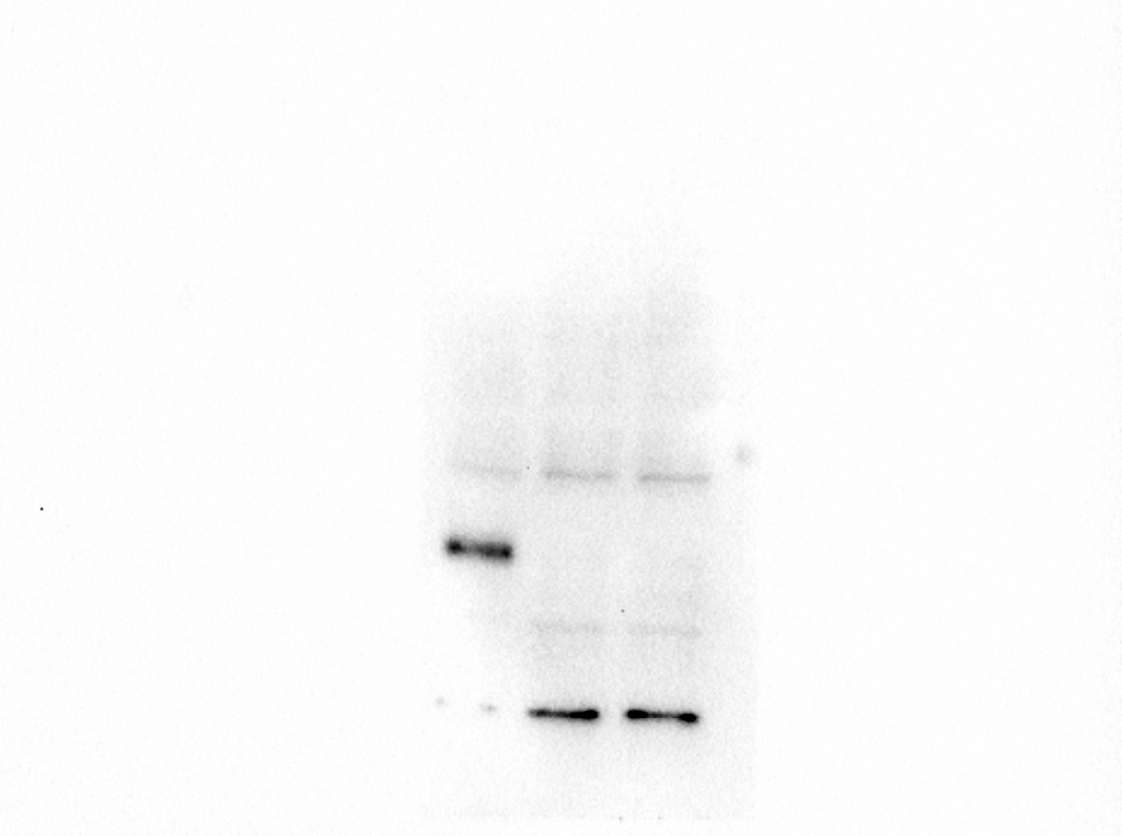

Supplement: Figure 3—figure supplement 1—source data 2. [file elife-100601-fig3-figsupp1-data2.zip › Figure 3-figure supplement 1-Source Data 2/Figure3-figure supplement 1a/WTAP.tif]

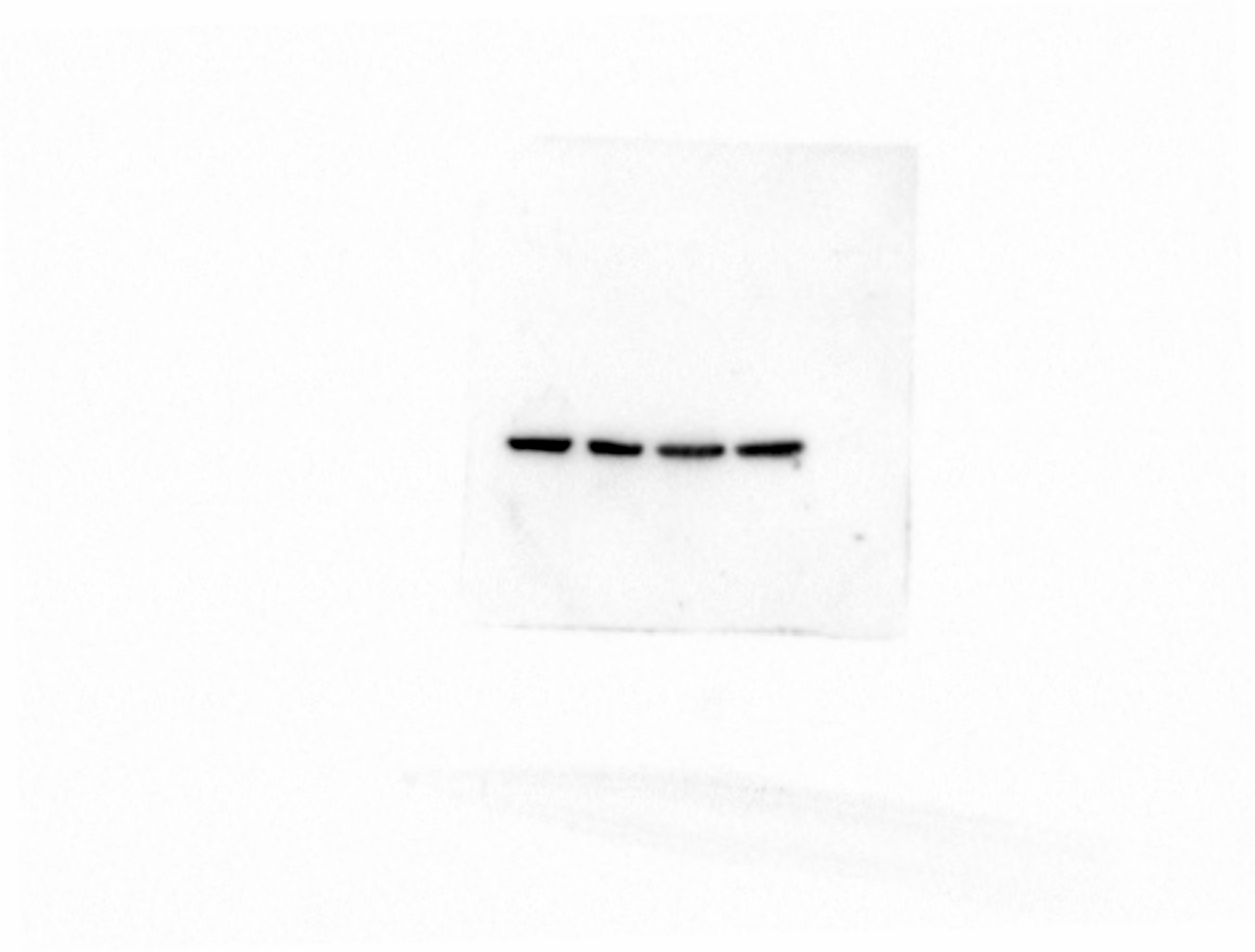

Supplement: Figure 3—figure supplement 1—source data 2. [file elife-100601-fig3-figsupp1-data2.zip › Figure 3-figure supplement 1-Source Data 2/Figure3-figure supplement 1c/actin.tif]

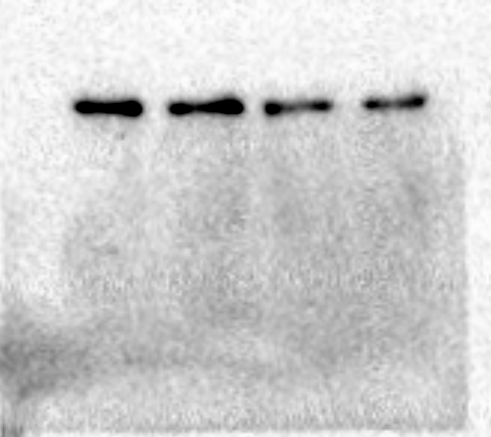

Supplement: Figure 3—figure supplement 1—source data 2. [file elife-100601-fig3-figsupp1-data2.zip › Figure 3-figure supplement 1-Source Data 2/Figure3-figure supplement 1c/IRF9.tif]

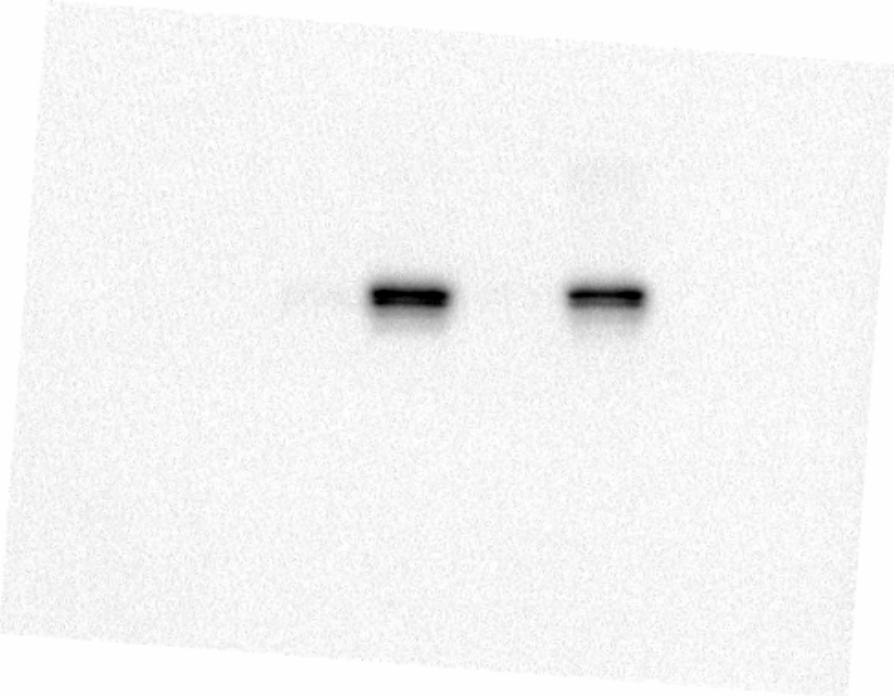

Supplement: Figure 3—figure supplement 1—source data 2. [file elife-100601-fig3-figsupp1-data2.zip › Figure 3-figure supplement 1-Source Data 2/Figure3-figure supplement 1c/pSTAT1.tif]

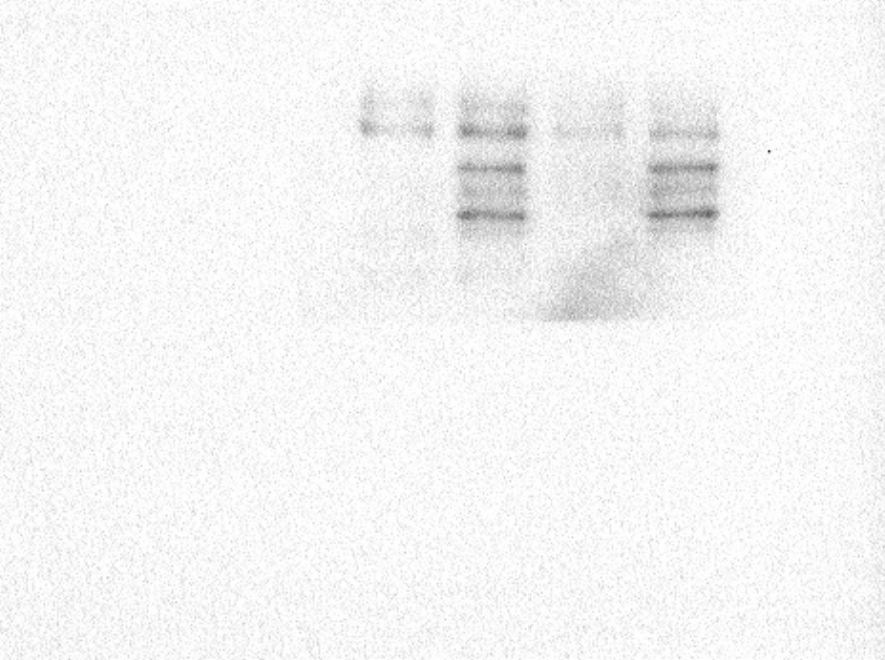

Supplement: Figure 3—figure supplement 1—source data 2. [file elife-100601-fig3-figsupp1-data2.zip › Figure 3-figure supplement 1-Source Data 2/Figure3-figure supplement 1c/pSTAT2.tif]

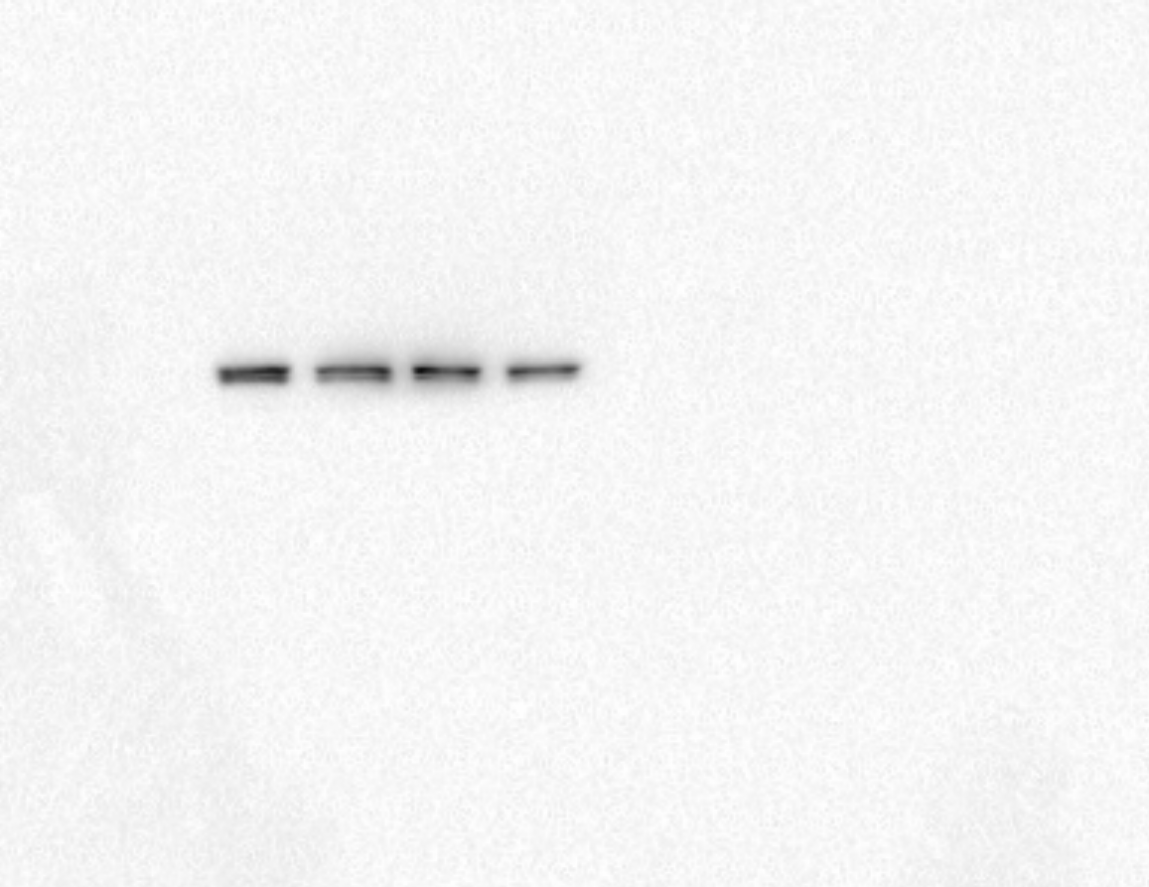

Supplement: Figure 3—figure supplement 1—source data 2. [file elife-100601-fig3-figsupp1-data2.zip › Figure 3-figure supplement 1-Source Data 2/Figure3-figure supplement 1c/STAT1.tif]

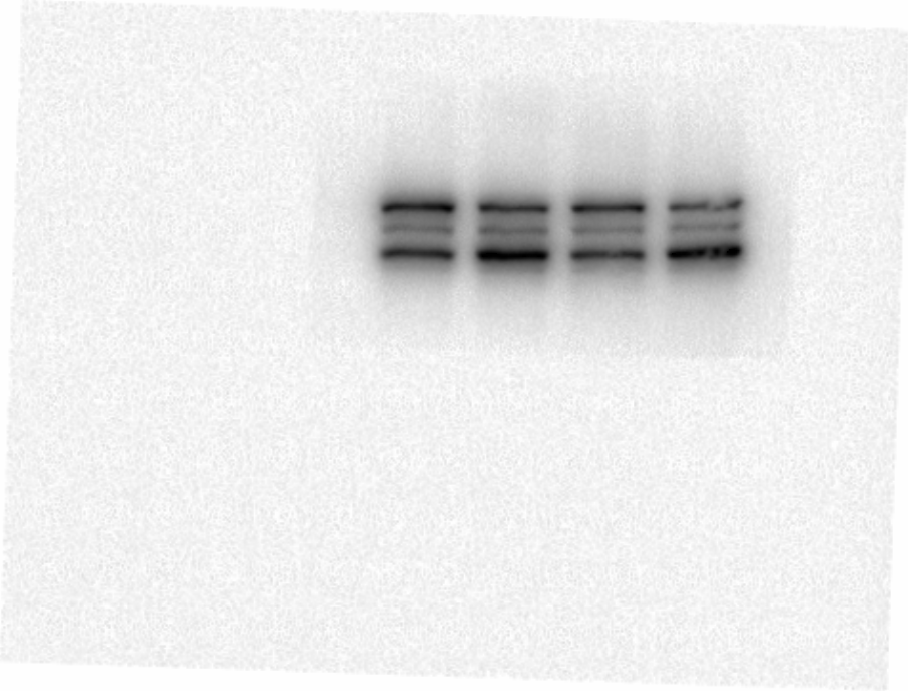

Supplement: Figure 3—figure supplement 1—source data 2. [file elife-100601-fig3-figsupp1-data2.zip › Figure 3-figure supplement 1-Source Data 2/Figure3-figure supplement 1c/STAT2.tif]

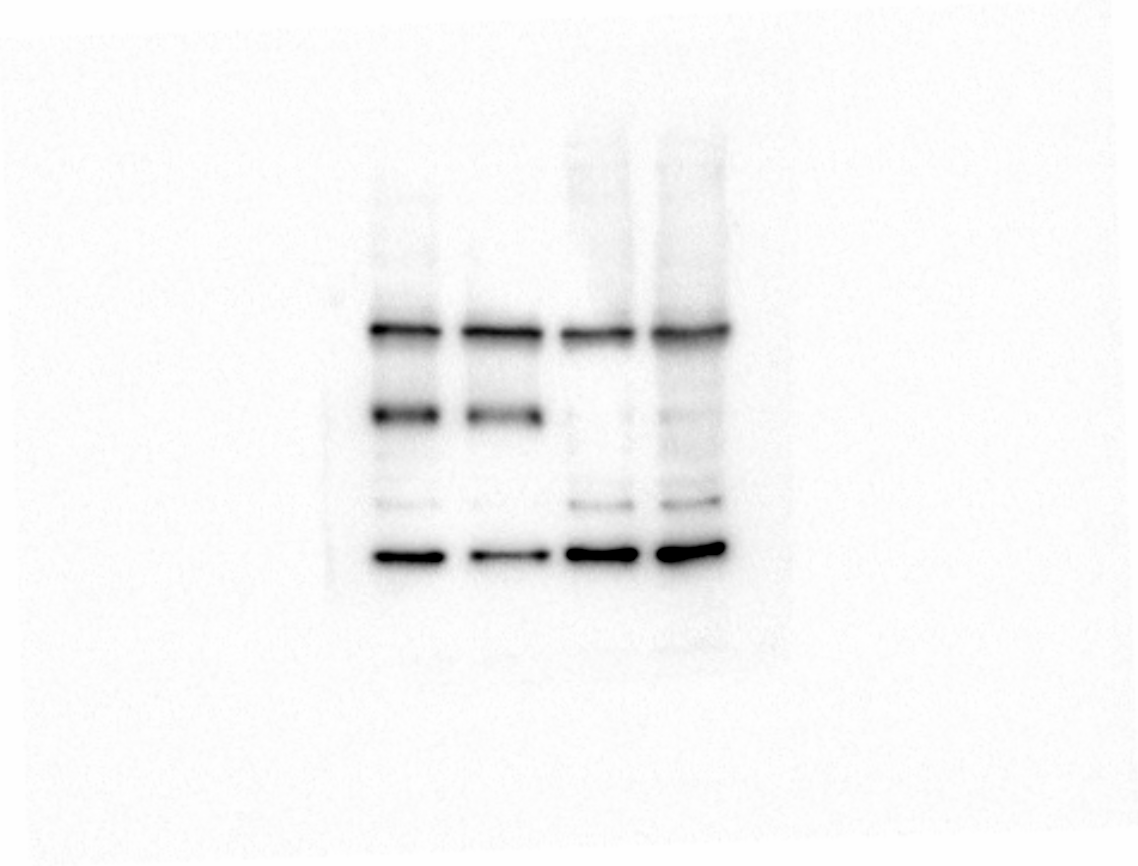

Supplement: Figure 3—figure supplement 1—source data 2. [file elife-100601-fig3-figsupp1-data2.zip › Figure 3-figure supplement 1-Source Data 2/Figure3-figure supplement 1c/WTAP.tif]

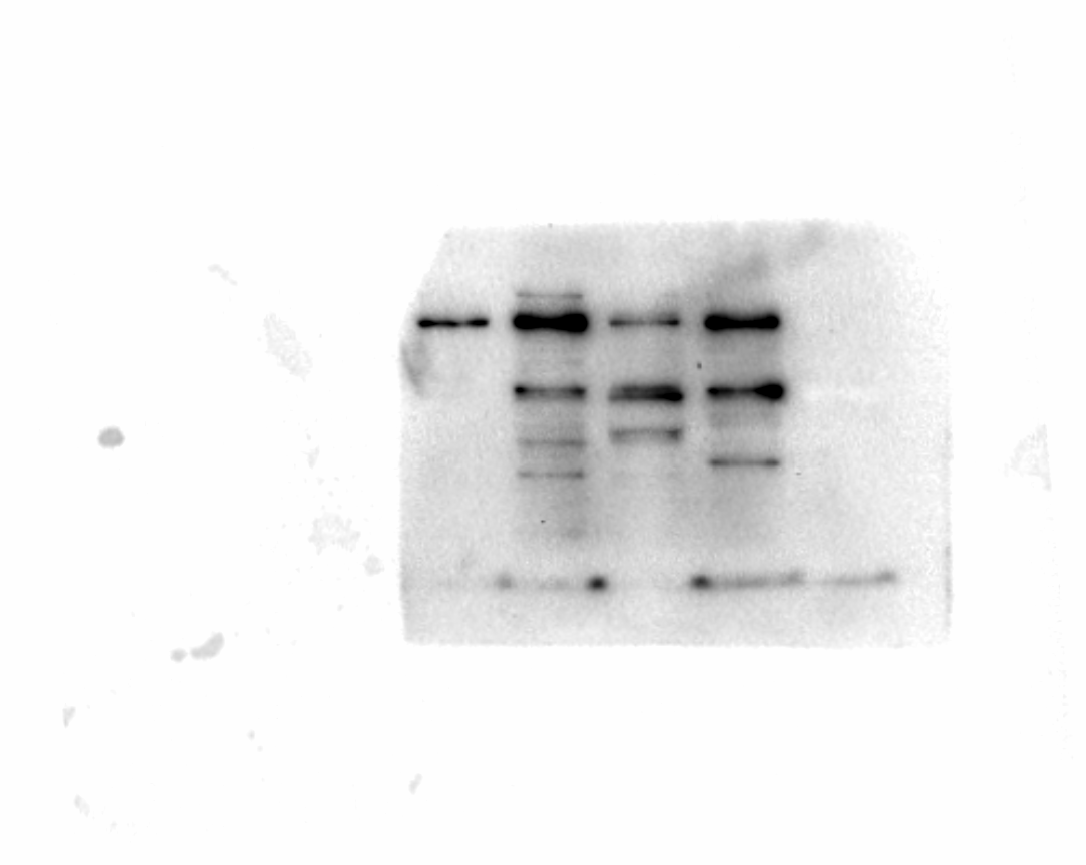

Supplement: Figure 4—figure supplement 1—source data 2. [file elife-100601-fig4-figsupp1-data2.zip › Figure 4-figure supplement 1-Source Data 2/Figure 4-figure supplement 1C/Flag.tif]

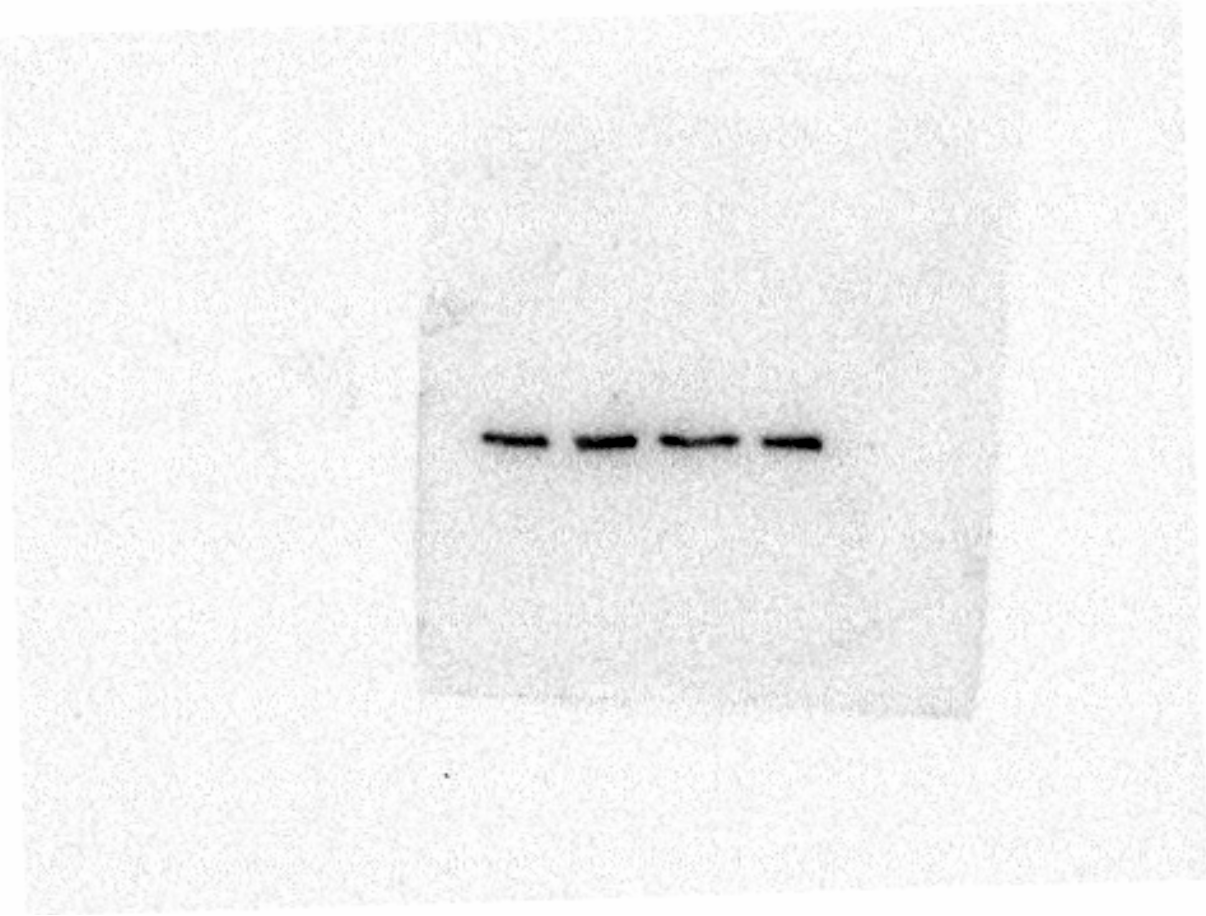

Supplement: Figure 4—figure supplement 1—source data 2. [file elife-100601-fig4-figsupp1-data2.zip › Figure 4-figure supplement 1-Source Data 2/Figure 4-figure supplement 1C/actin.tif]

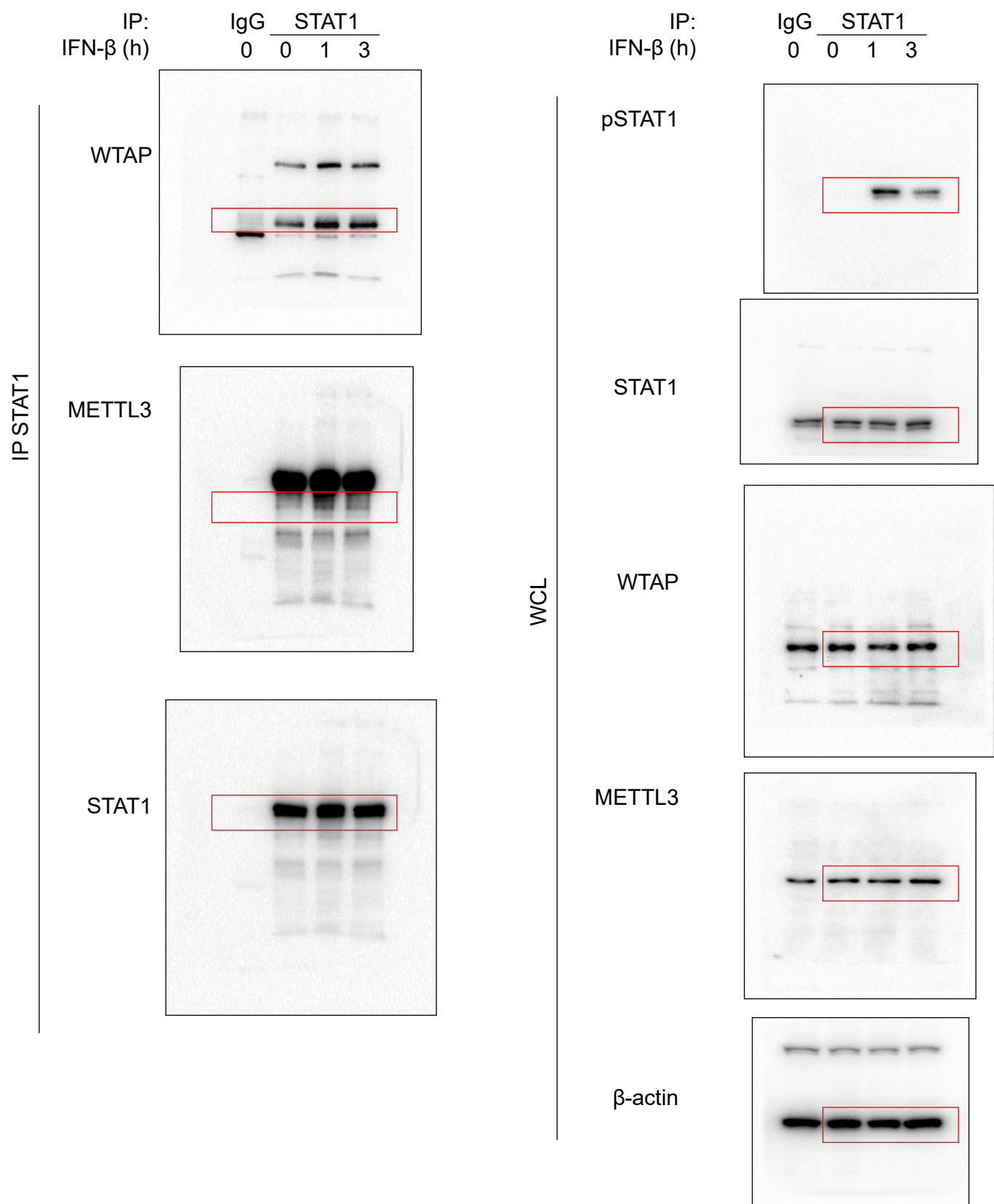

Figure 5B, source data 1: Original membranes corresponding to Figure 5B.

Supplement: Figure 5—source data 1. [file elife-100601-fig5-data1.zip › Figure 5-Source Data 1/Figure 5B.pdf]

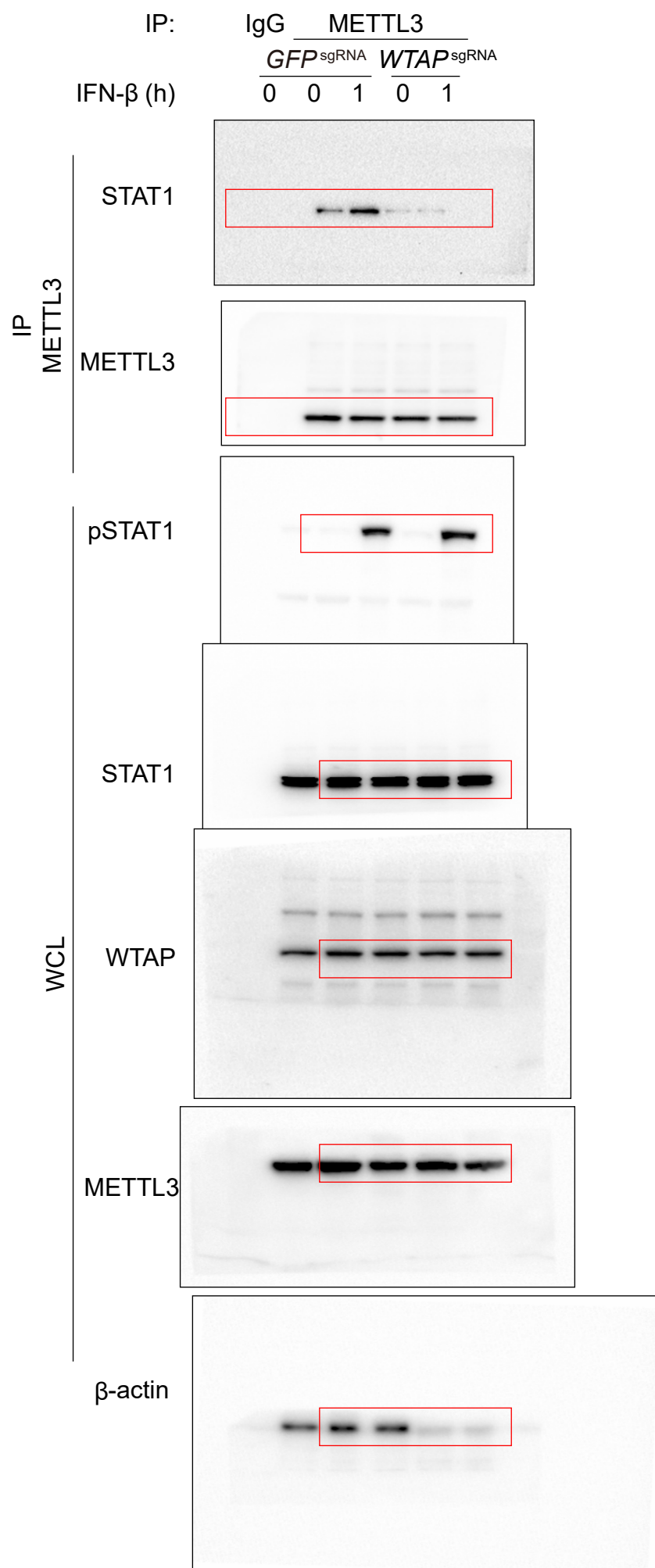

Figure 5C, source data 1: Original membranes corresponding to Figure 5C.

Supplement: Figure 5—source data 1. [file elife-100601-fig5-data1.zip › Figure 5-Source Data 1/Figure 5C.pdf]

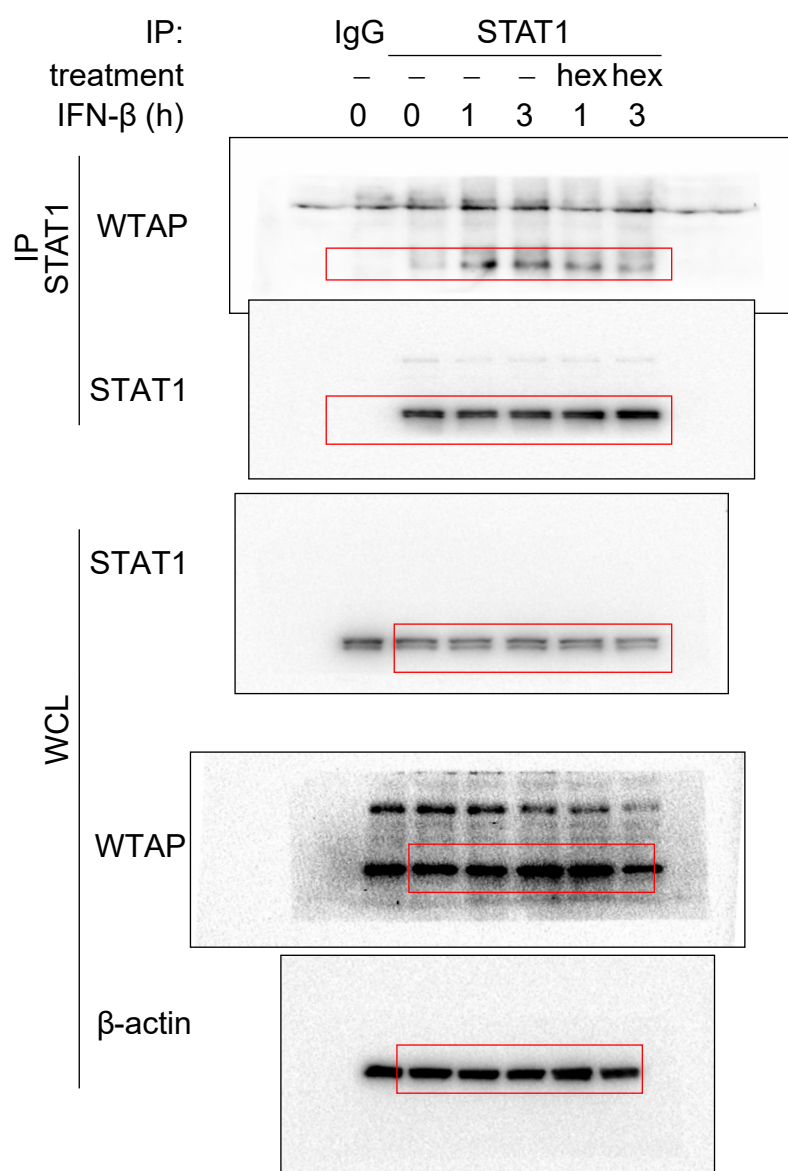

Figure 5E, source data 1: Original membranes corresponding to Figure 5E.

Supplement: Figure 5—source data 1. [file elife-100601-fig5-data1.zip › Figure 5-Source Data 1/Figure 5E.pdf]

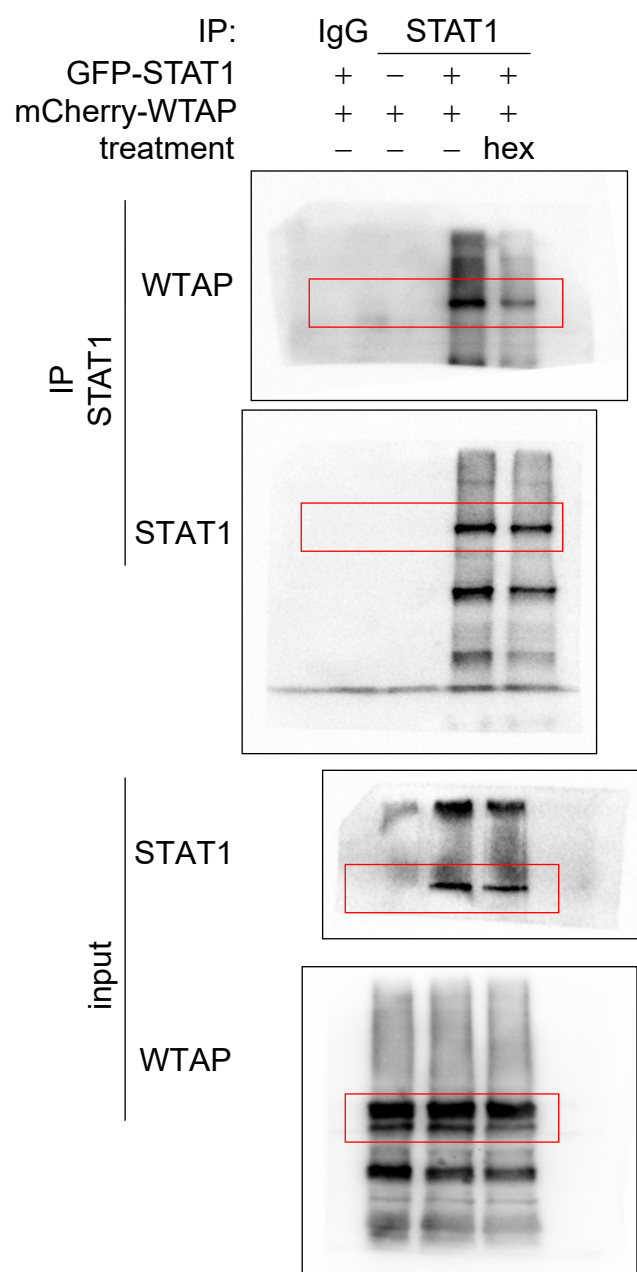

Figure 5F, source data 1: Original membranes corresponding to Figure 5F.

Supplement: Figure 5—source data 1. [file elife-100601-fig5-data1.zip › Figure 5-Source Data 1/Figure 5F.pdf]

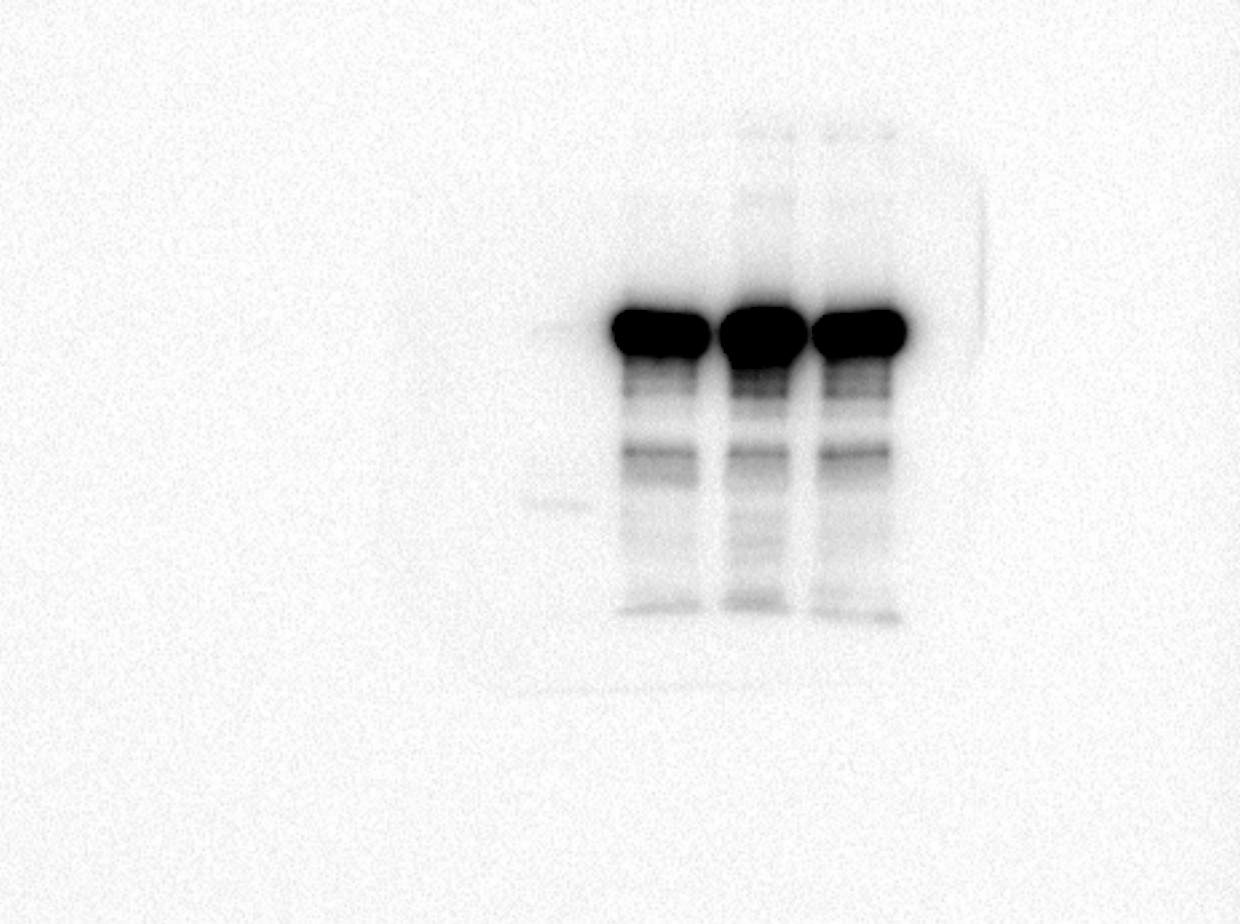

Supplement: Figure 5—source data 2. [file elife-100601-fig5-data2.zip › Figure 5-Source Data 2/Figure5b/IP-METTL3.tif]

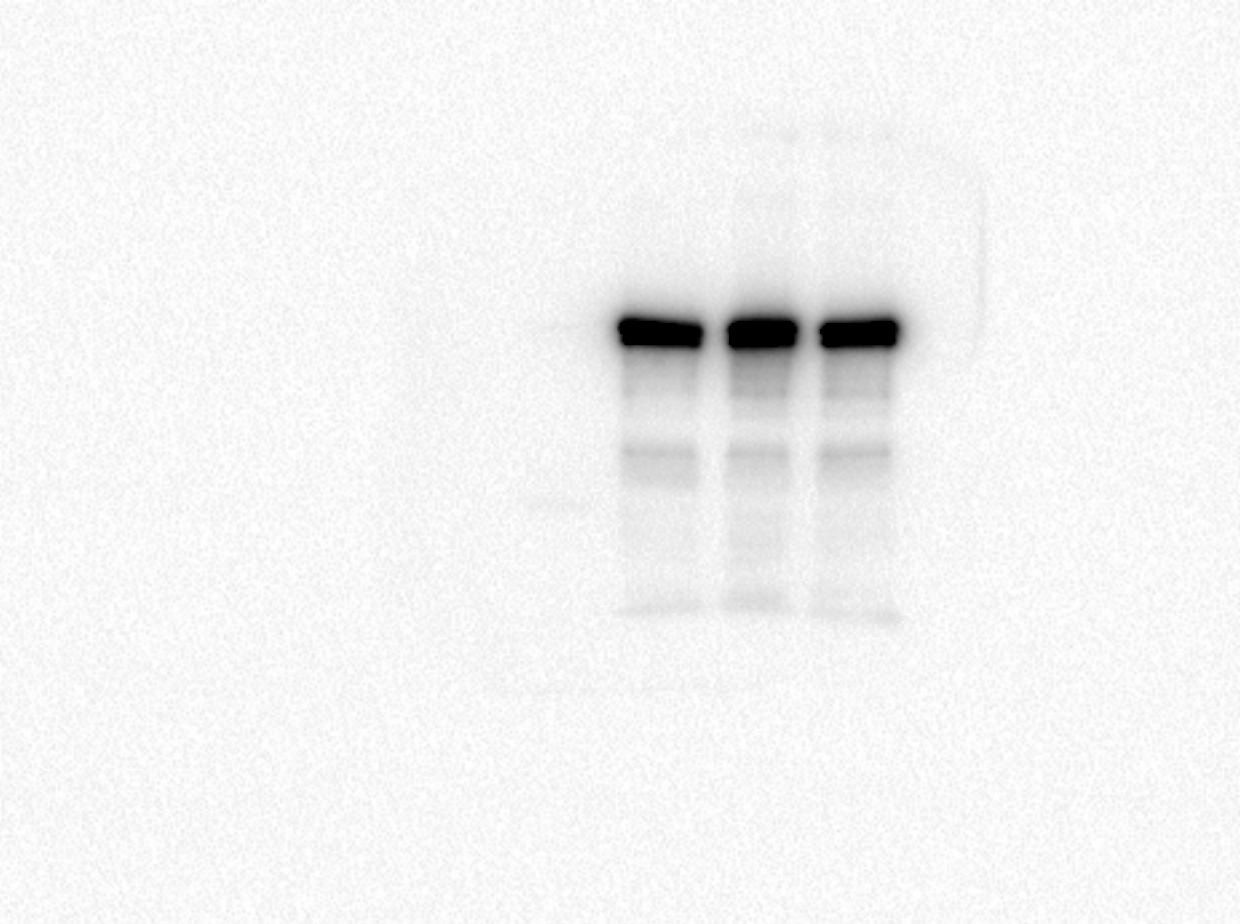

Supplement: Figure 5—source data 2. [file elife-100601-fig5-data2.zip › Figure 5-Source Data 2/Figure5b/IP-STAT1.tif]

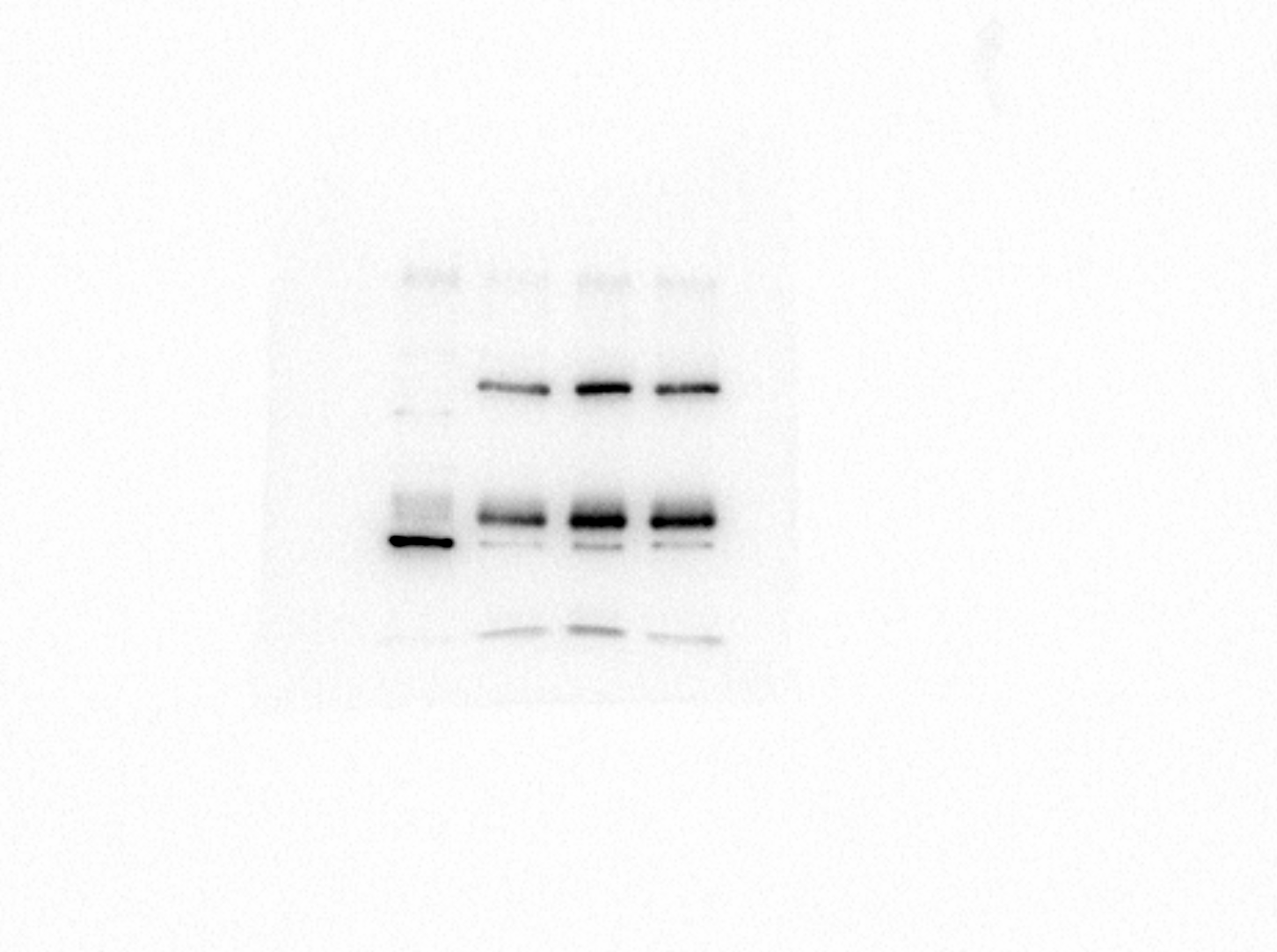

Supplement: Figure 5—source data 2. [file elife-100601-fig5-data2.zip › Figure 5-Source Data 2/Figure5b/IP-WTAP.tif]

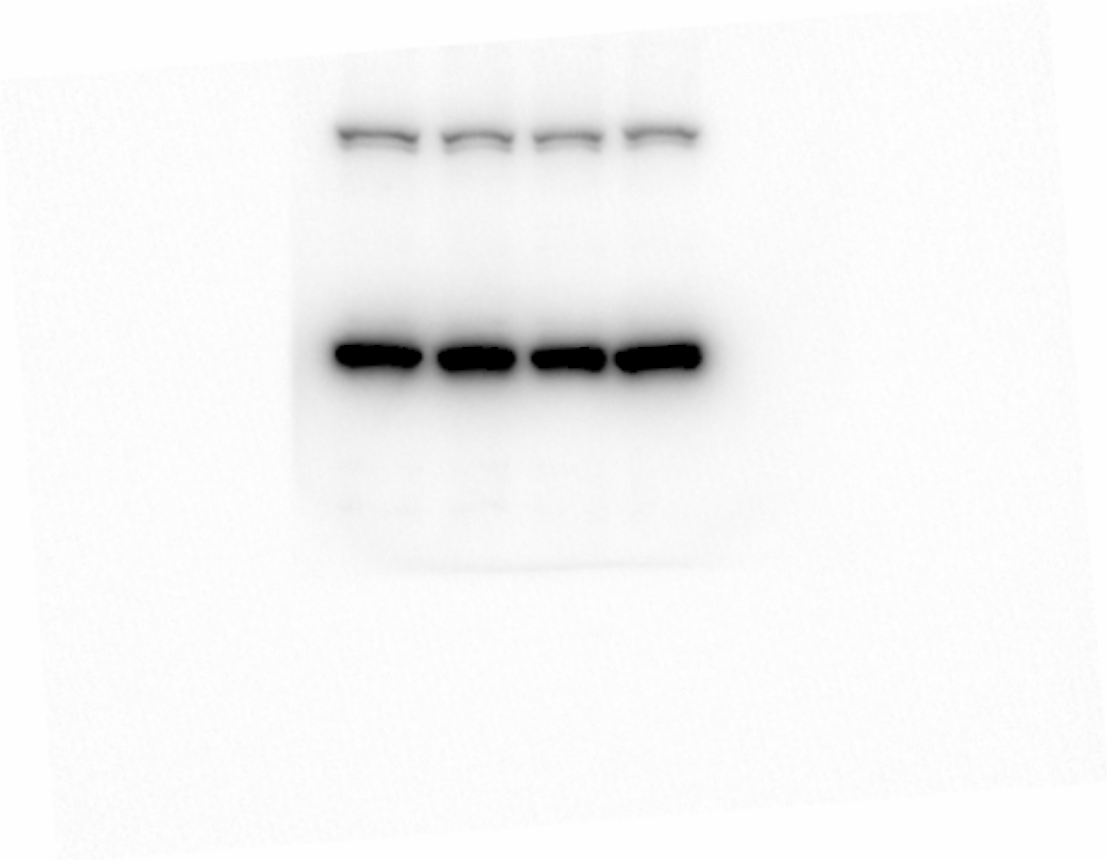

Supplement: Figure 5—source data 2. [file elife-100601-fig5-data2.zip › Figure 5-Source Data 2/Figure5b/actin.tif]

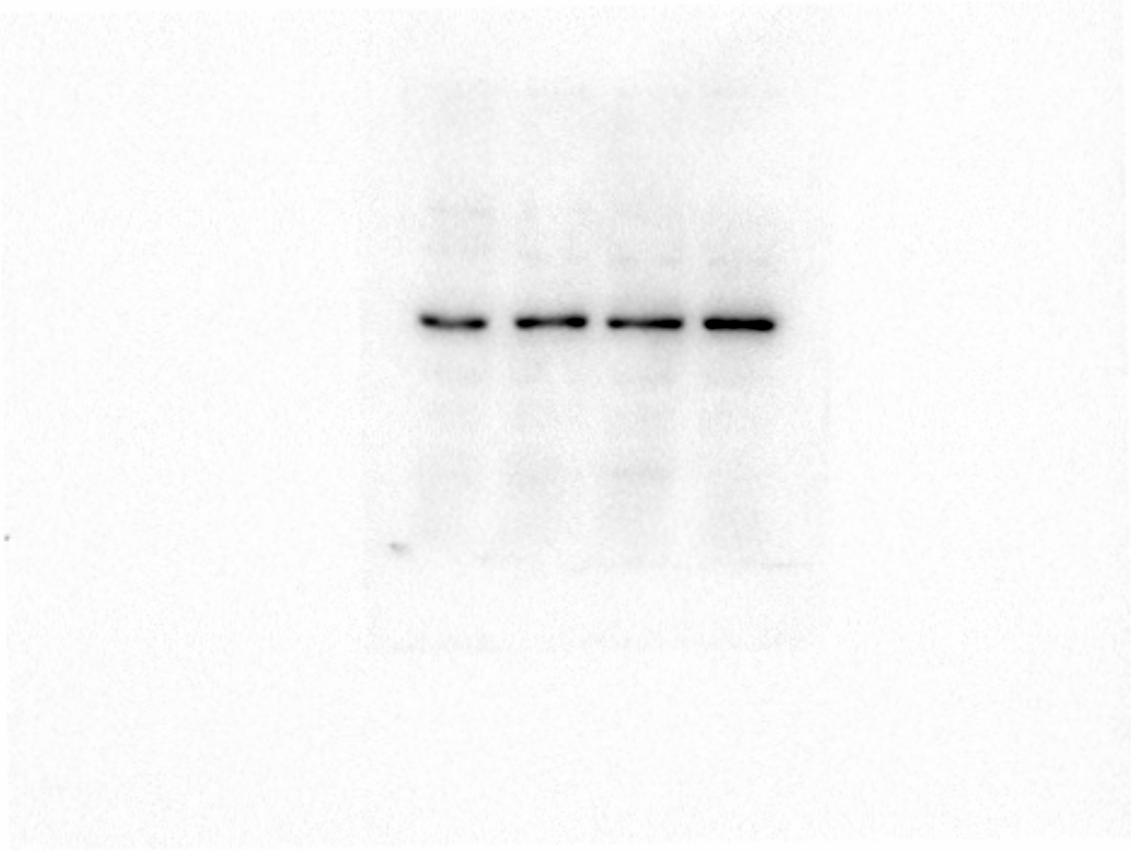

Supplement: Figure 5—source data 2. [file elife-100601-fig5-data2.zip › Figure 5-Source Data 2/Figure5b/METTL3.tif]

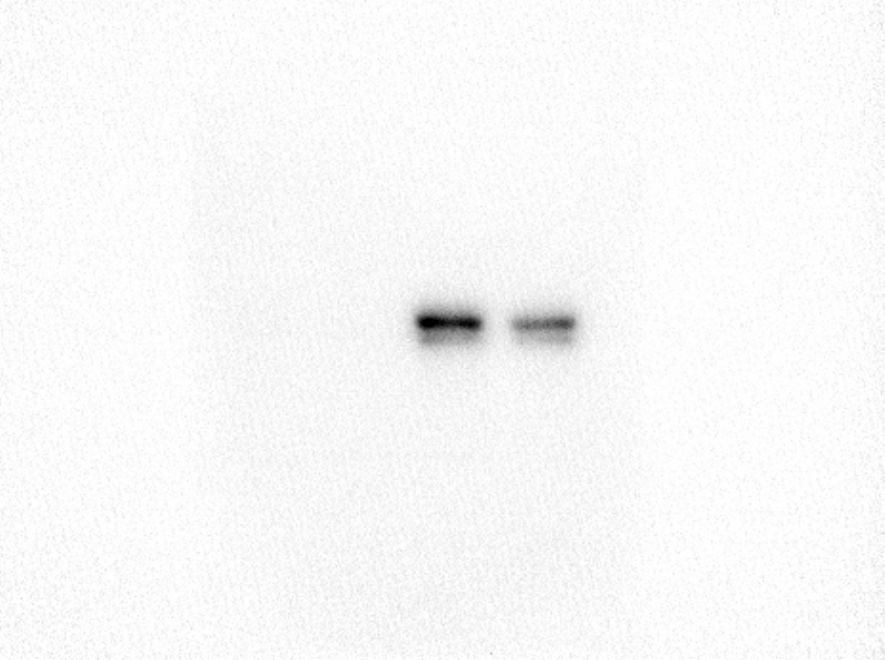

Supplement: Figure 5—source data 2. [file elife-100601-fig5-data2.zip › Figure 5-Source Data 2/Figure5b/pSTAT1.tif]

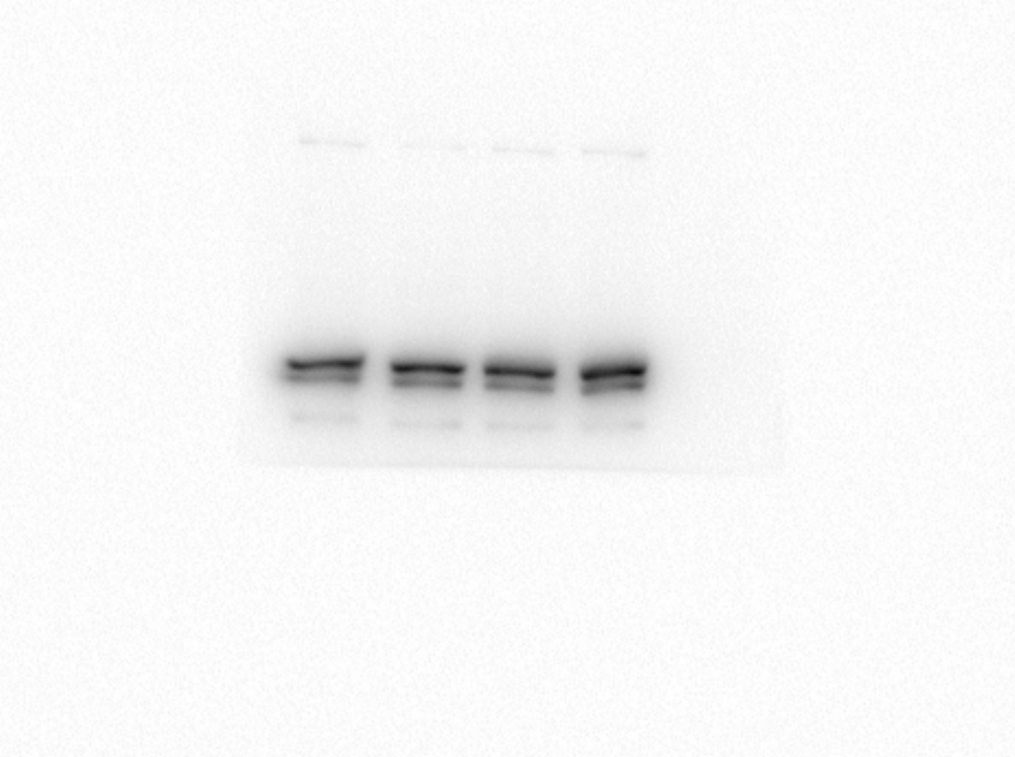

Supplement: Figure 5—source data 2. [file elife-100601-fig5-data2.zip › Figure 5-Source Data 2/Figure5b/STAT1.tif]

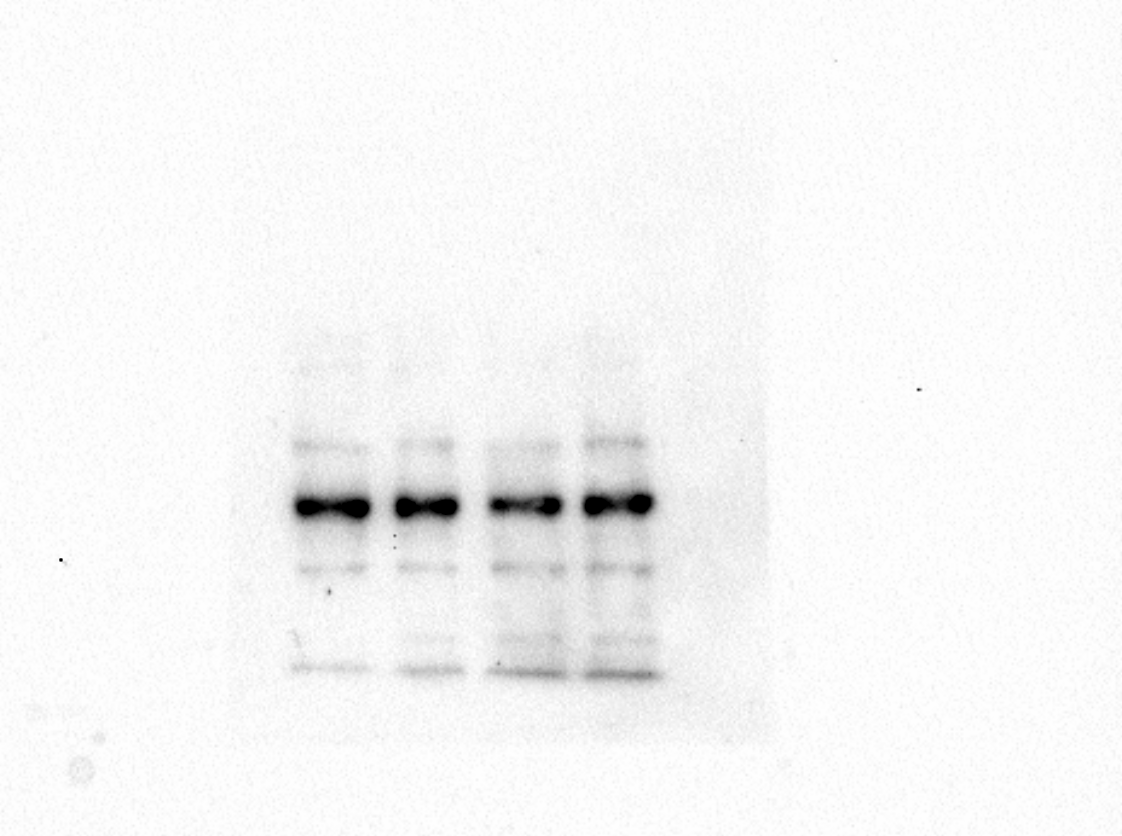

Supplement: Figure 5—source data 2. [file elife-100601-fig5-data2.zip › Figure 5-Source Data 2/Figure5b/WTAP.tif]

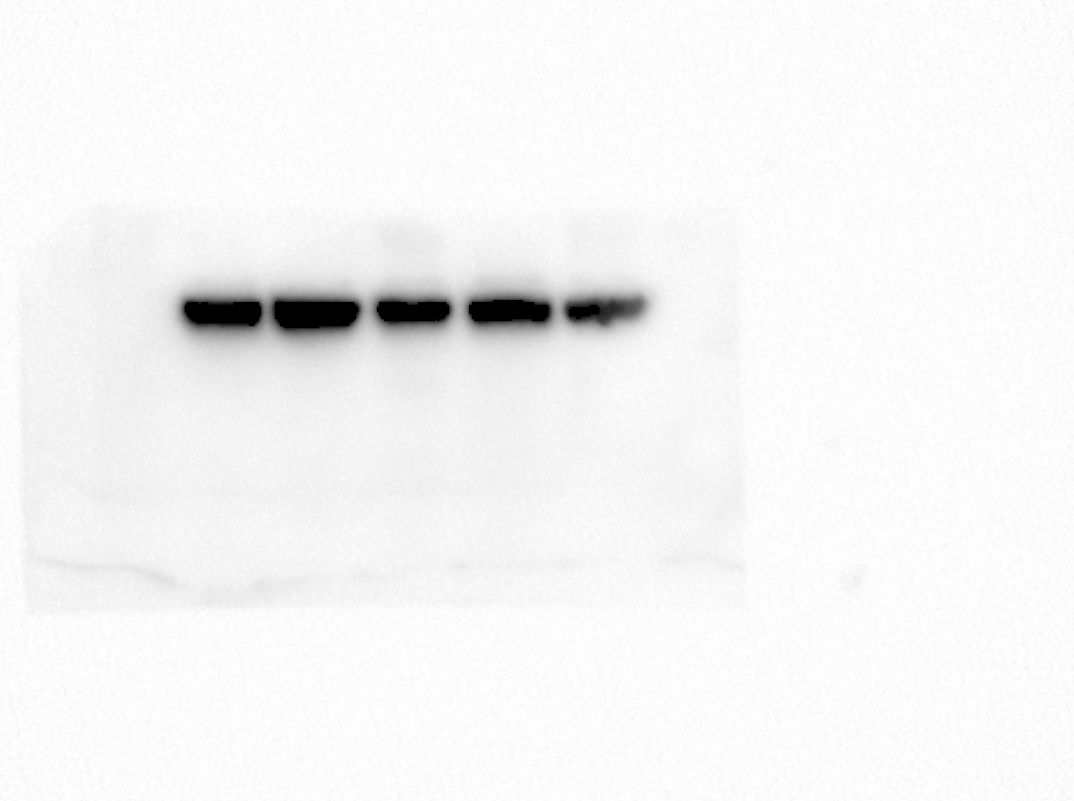

Supplement: Figure 5—source data 2. [file elife-100601-fig5-data2.zip › Figure 5-Source Data 2/Figure5c/actin.tif]

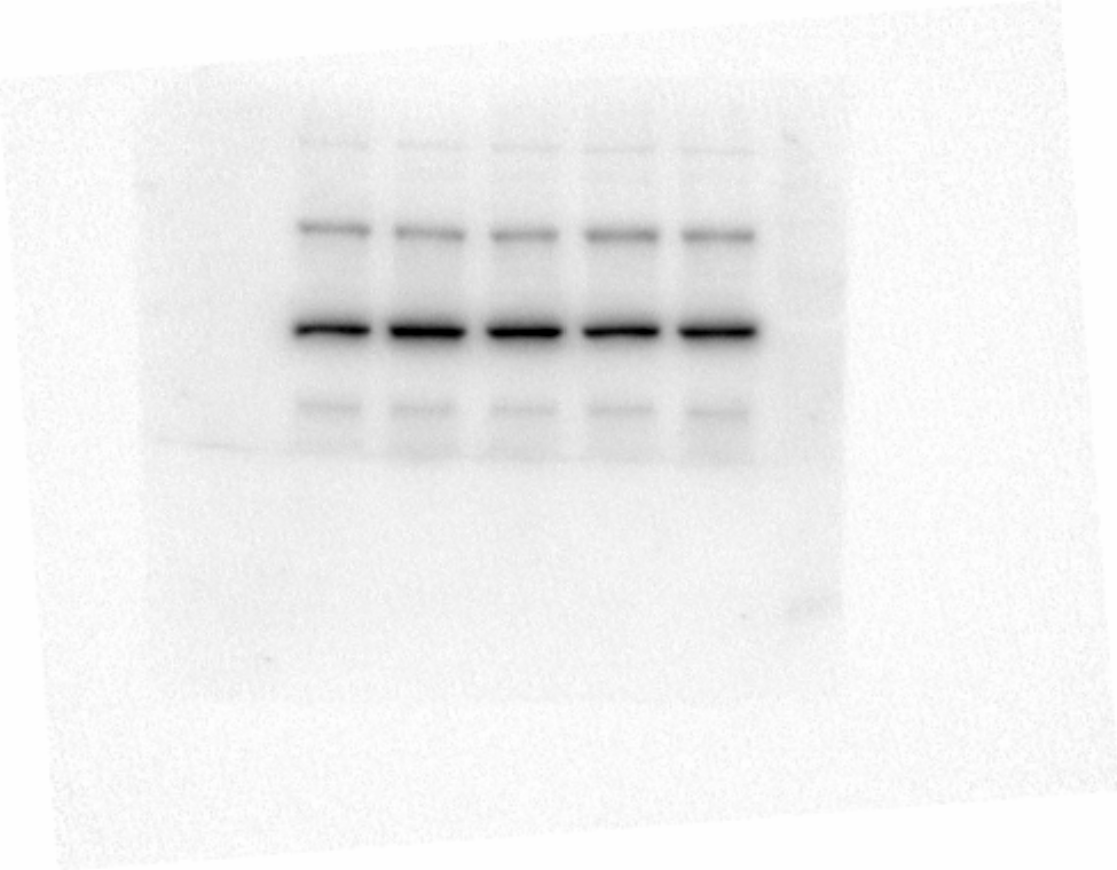

Supplement: Figure 5—source data 2. [file elife-100601-fig5-data2.zip › Figure 5-Source Data 2/Figure5c/METTL3.tif]

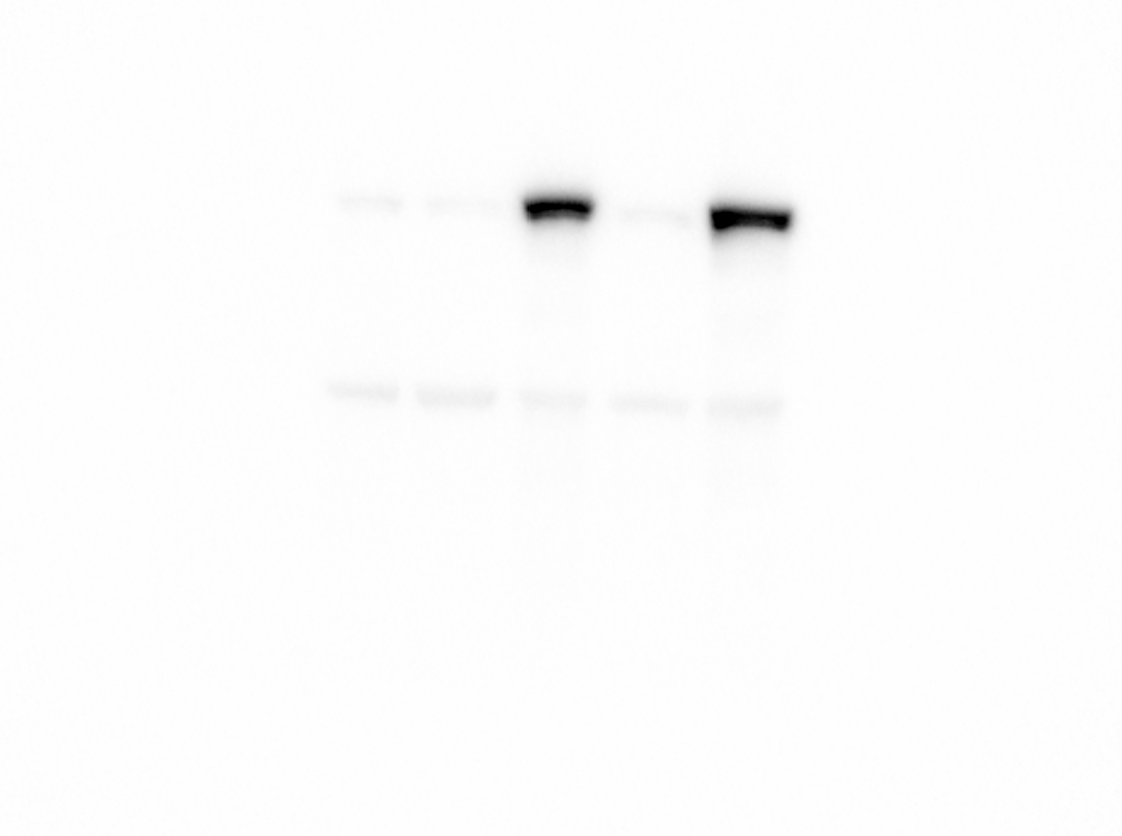

Supplement: Figure 5—source data 2. [file elife-100601-fig5-data2.zip › Figure 5-Source Data 2/Figure5c/pSTAT1.tif]

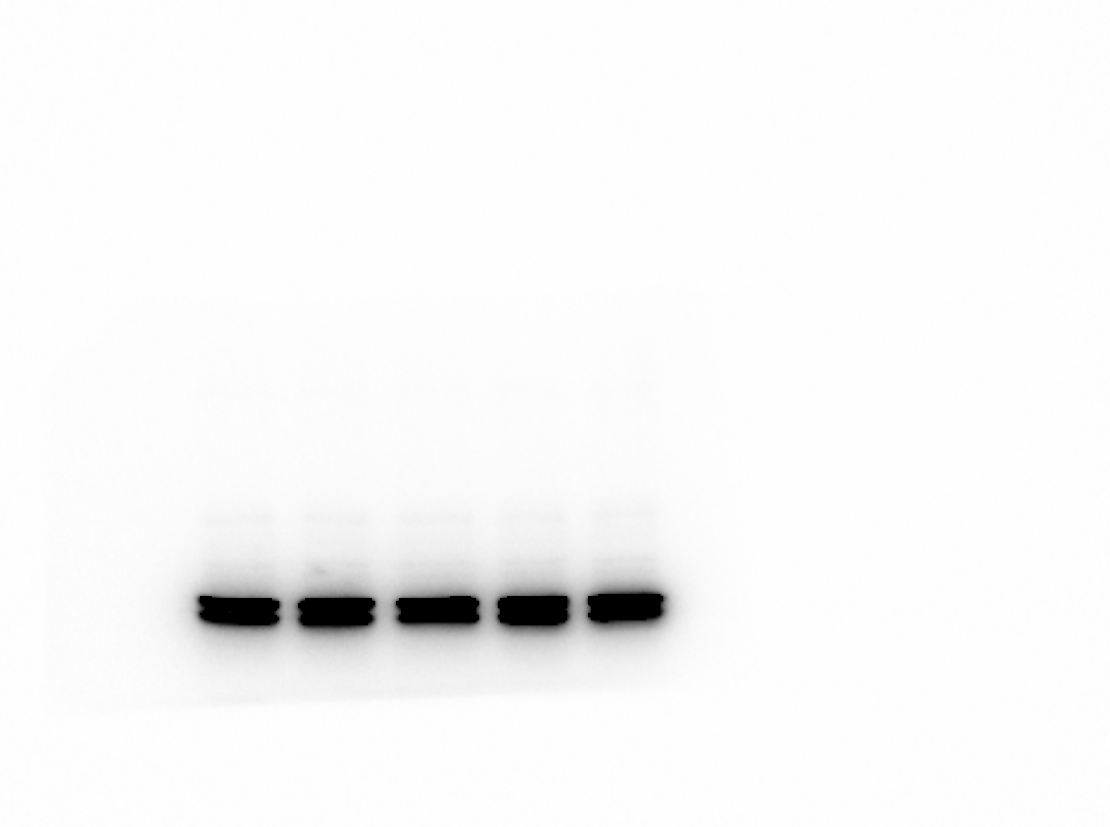

Supplement: Figure 5—source data 2. [file elife-100601-fig5-data2.zip › Figure 5-Source Data 2/Figure5c/STAT1.tif]

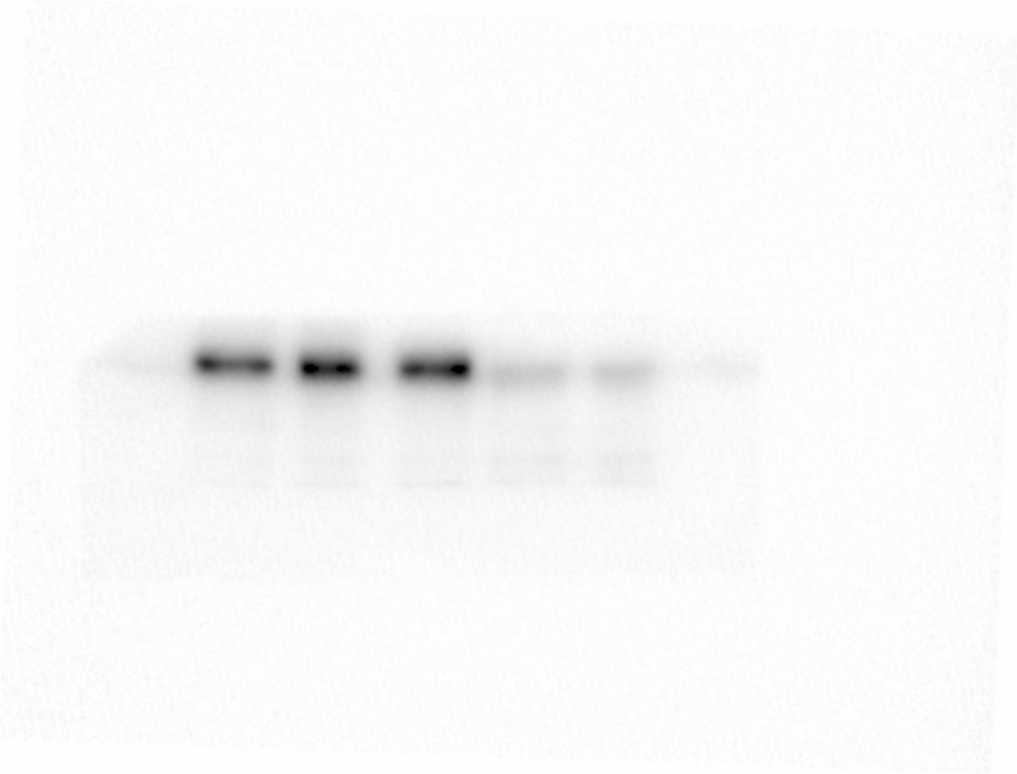

Supplement: Figure 5—source data 2. [file elife-100601-fig5-data2.zip › Figure 5-Source Data 2/Figure5c/WTAP.tif]

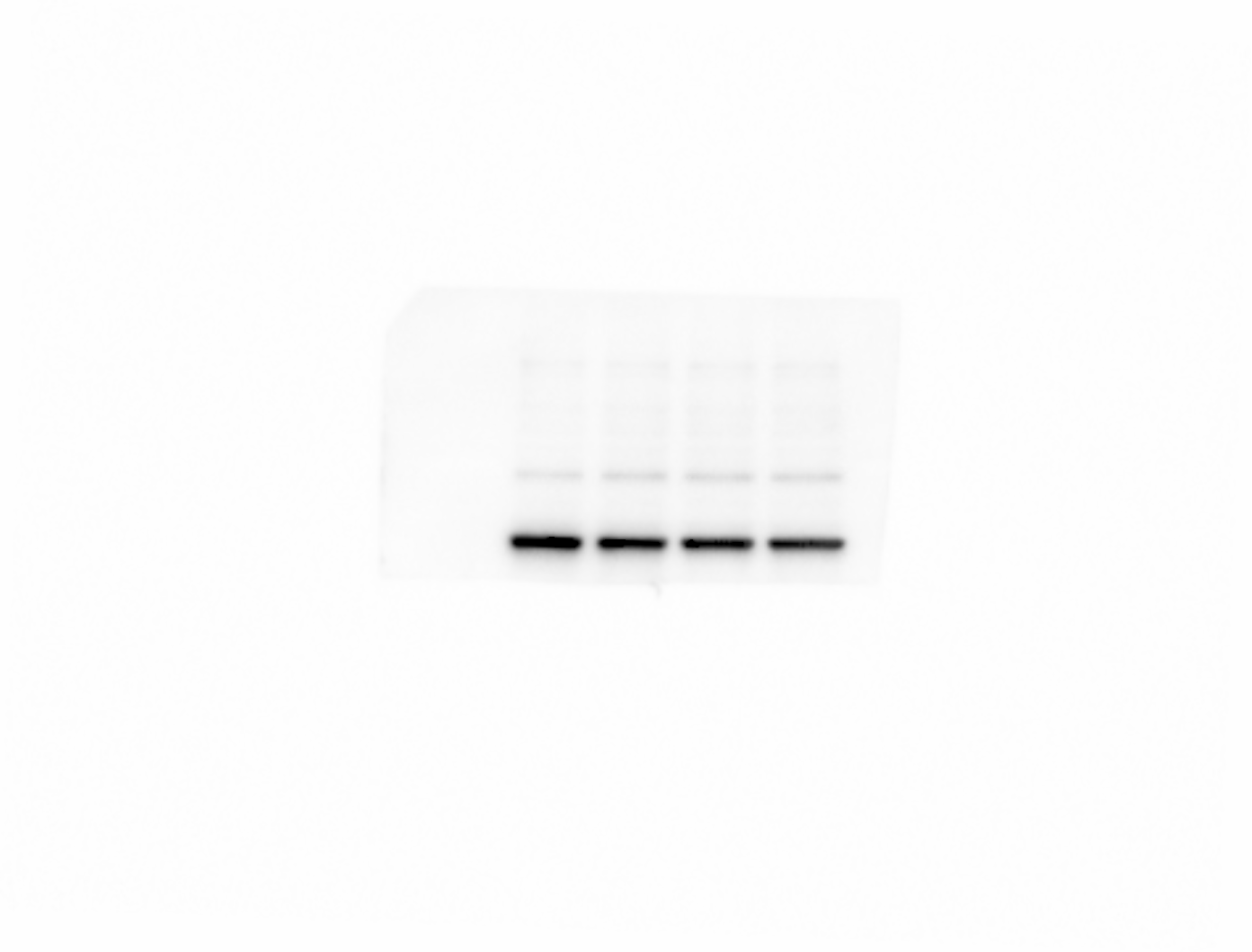

Supplement: Figure 5—source data 2. [file elife-100601-fig5-data2.zip › Figure 5-Source Data 2/Figure5c/IP-METTL3.tif]

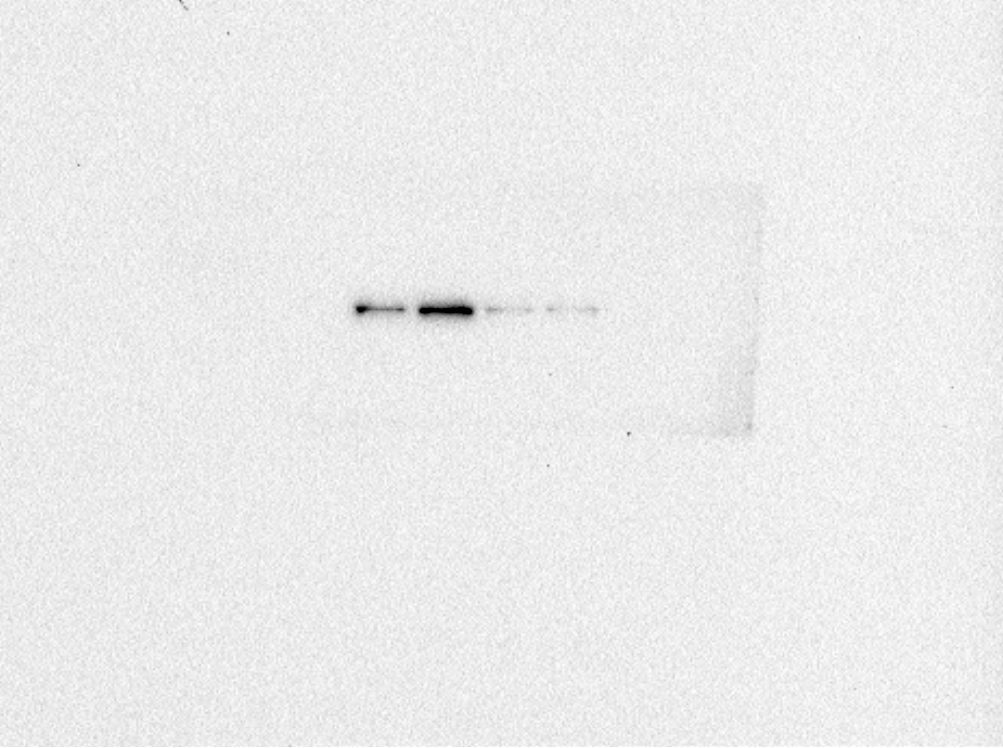

Supplement: Figure 5—source data 2. [file elife-100601-fig5-data2.zip › Figure 5-Source Data 2/Figure5c/IP-STAT1.tif]

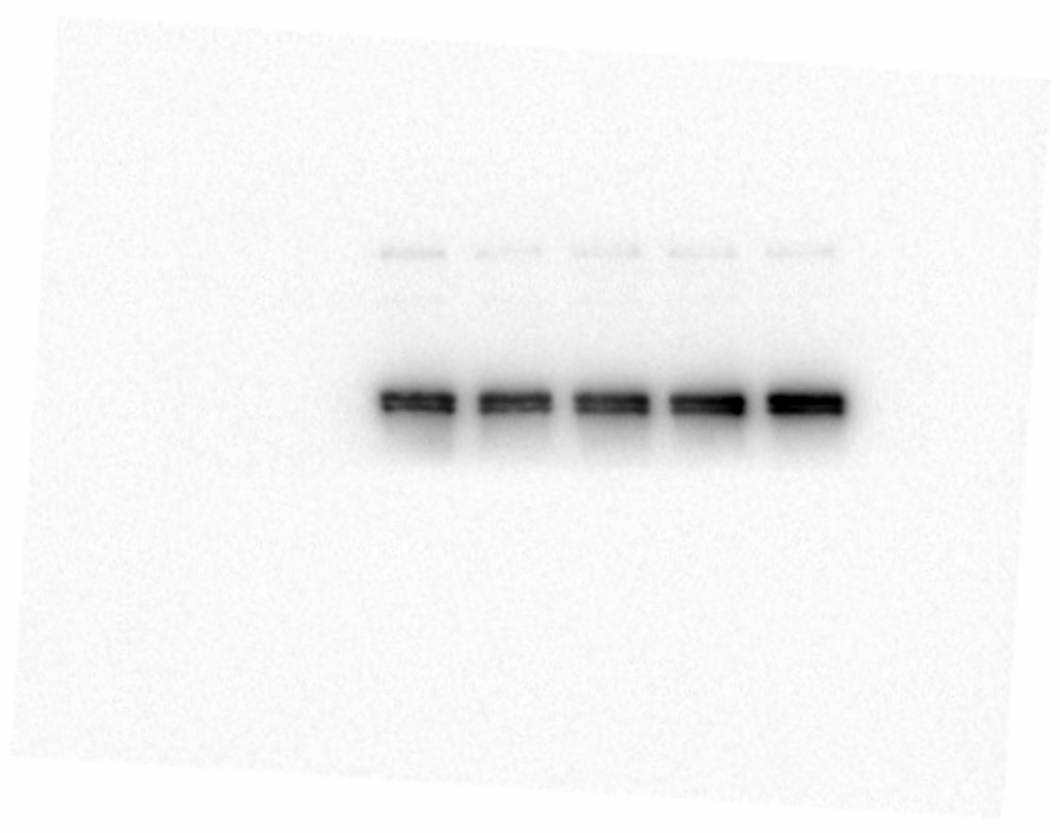

Supplement: Figure 5—source data 2. [file elife-100601-fig5-data2.zip › Figure 5-Source Data 2/Figure5e/IP-STAT1.tif]

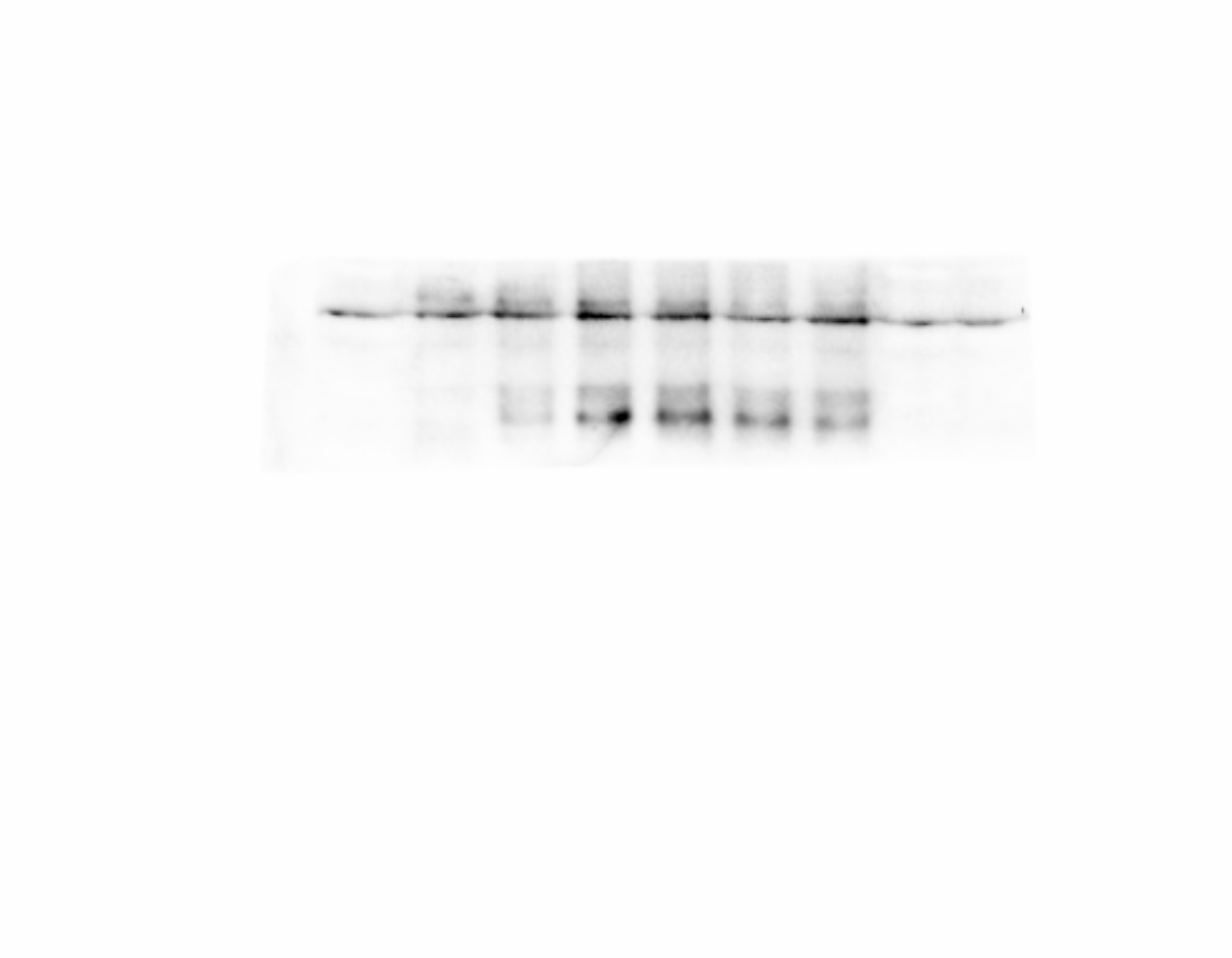

Supplement: Figure 5—source data 2. [file elife-100601-fig5-data2.zip › Figure 5-Source Data 2/Figure5e/IP-WTAP.tif]

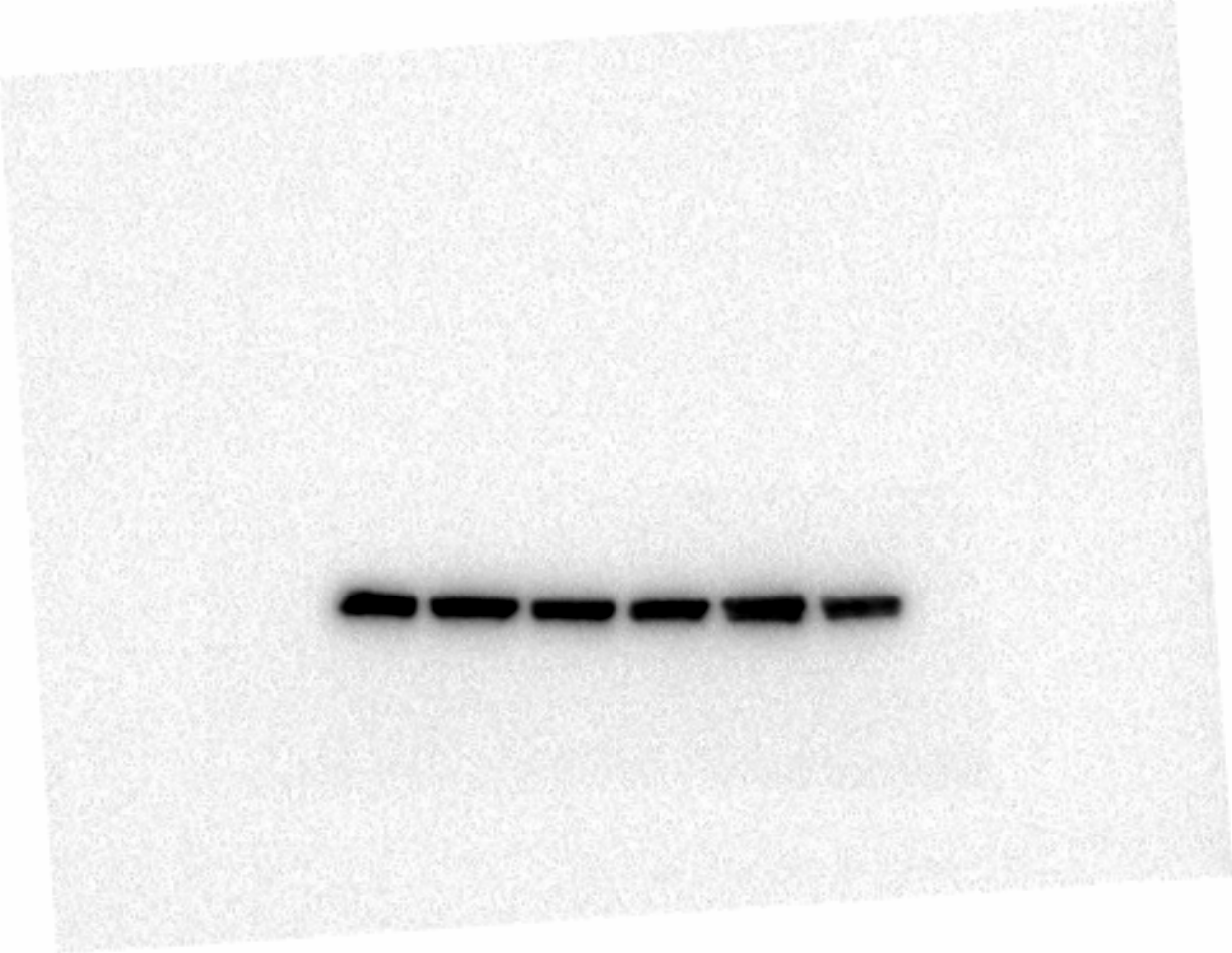

Supplement: Figure 5—source data 2. [file elife-100601-fig5-data2.zip › Figure 5-Source Data 2/Figure5e/actin.tif]

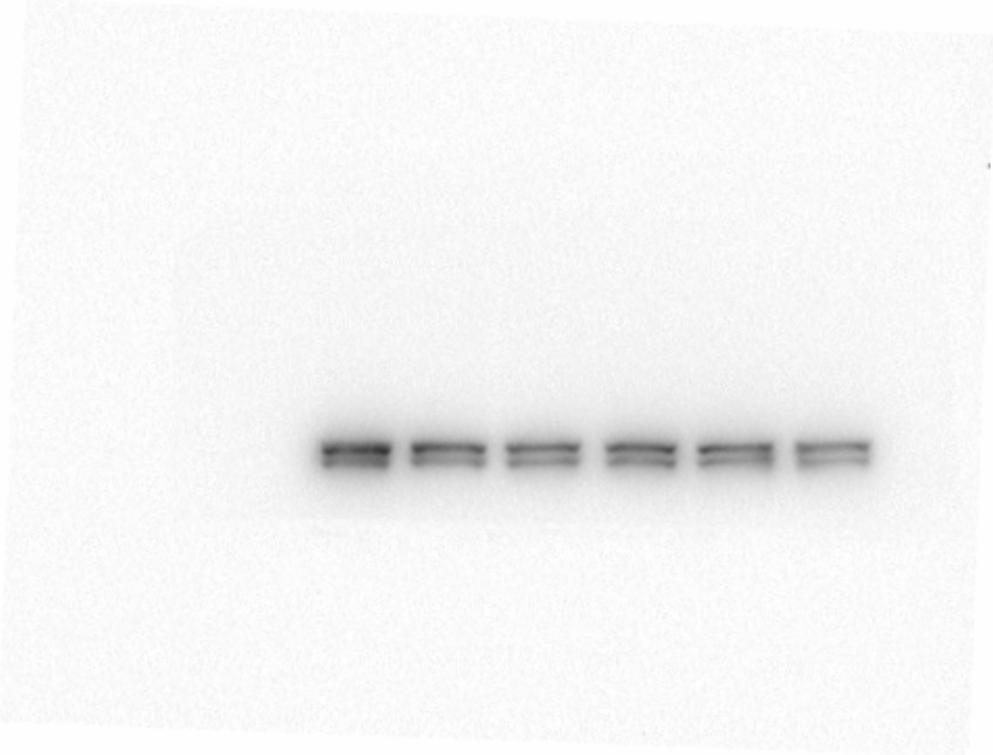

Supplement: Figure 5—source data 2. [file elife-100601-fig5-data2.zip › Figure 5-Source Data 2/Figure5e/STAT1.tif]

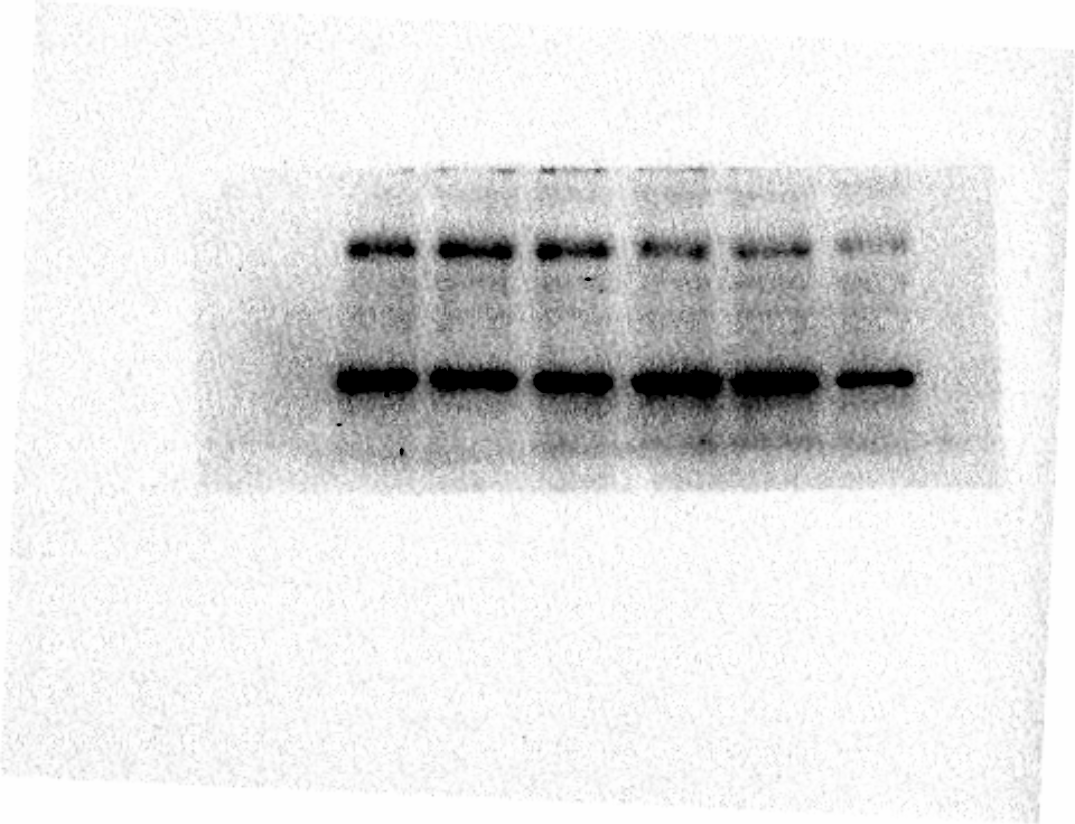

Supplement: Figure 5—source data 2. [file elife-100601-fig5-data2.zip › Figure 5-Source Data 2/Figure5e/WTAP.tif]

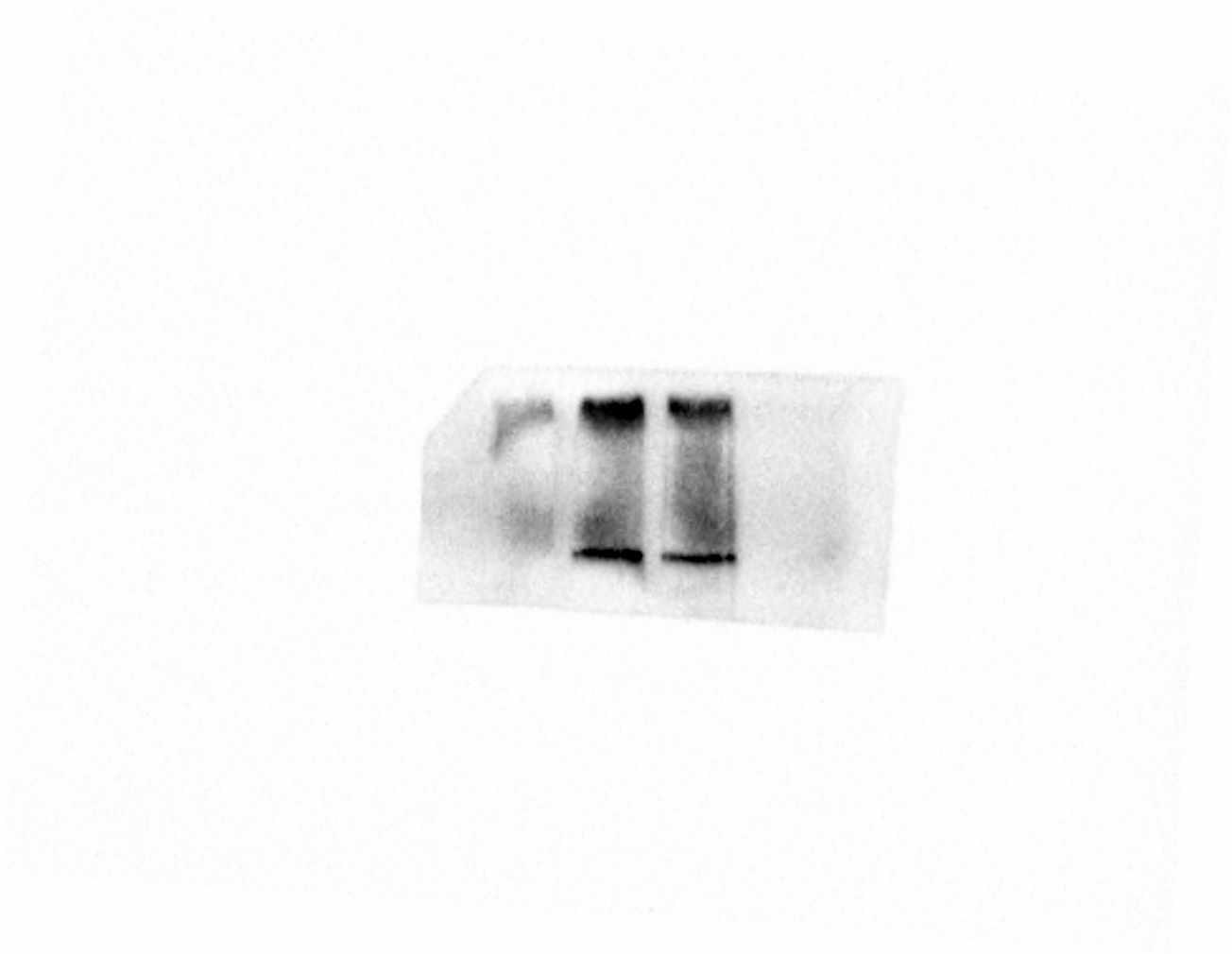

Supplement: Figure 5—source data 2. [file elife-100601-fig5-data2.zip › Figure 5-Source Data 2/Figure5f/input-STAT1.tif]

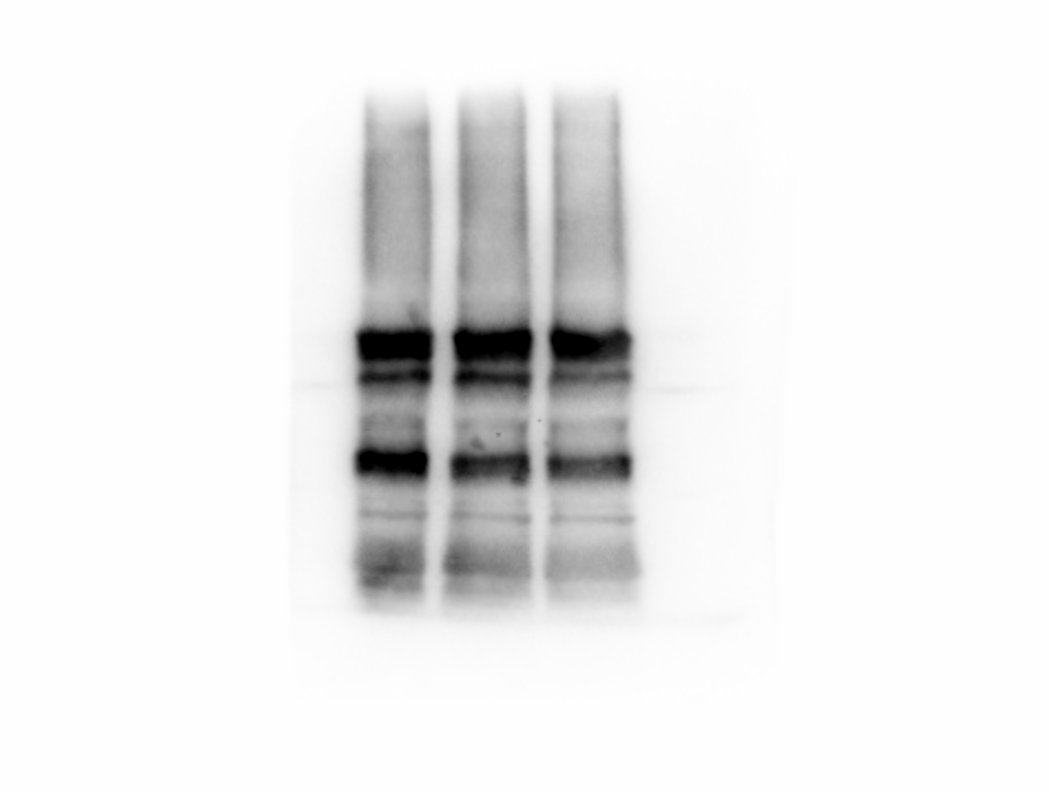

Supplement: Figure 5—source data 2. [file elife-100601-fig5-data2.zip › Figure 5-Source Data 2/Figure5f/input-WTAP.tif]

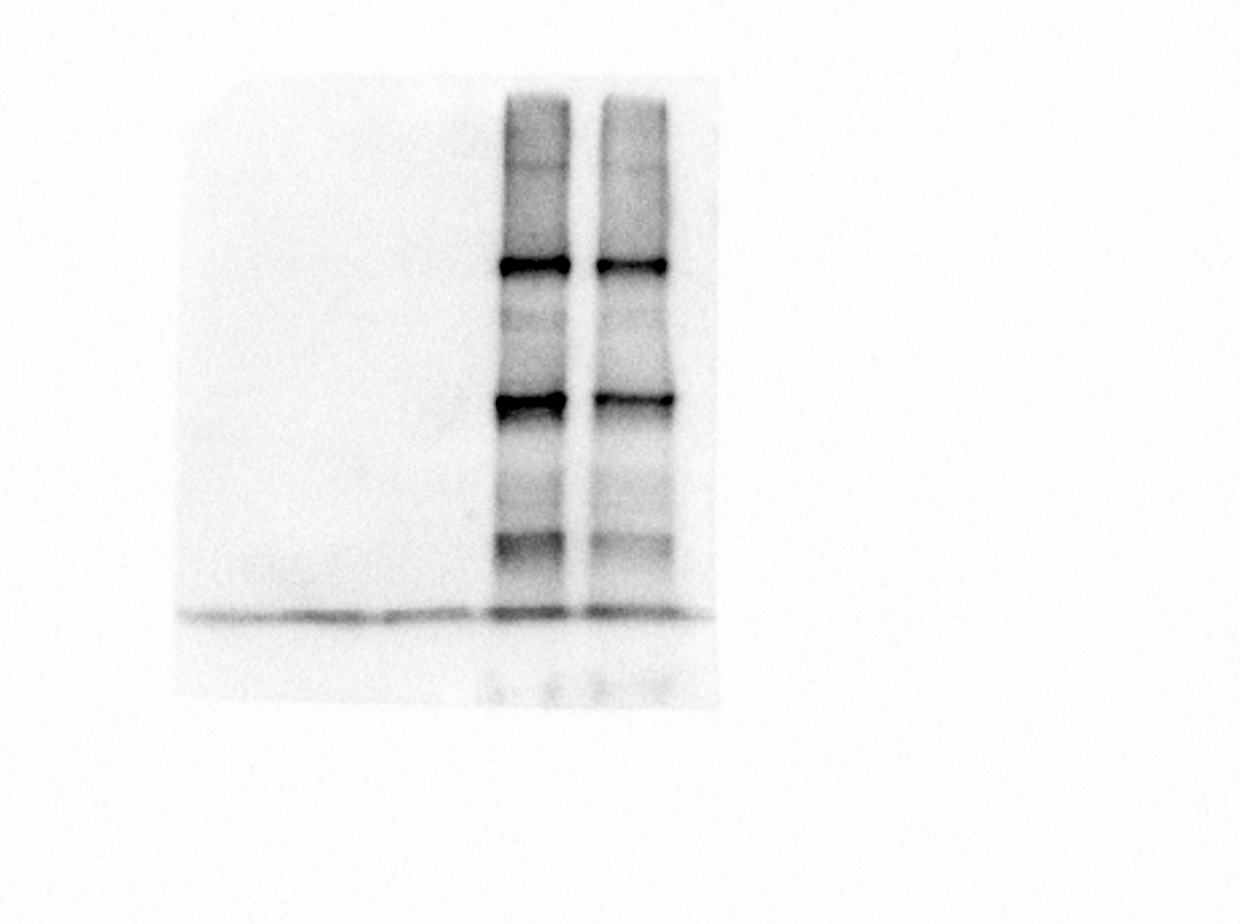

Supplement: Figure 5—source data 2. [file elife-100601-fig5-data2.zip › Figure 5-Source Data 2/Figure5f/IP-STAT1.tif]

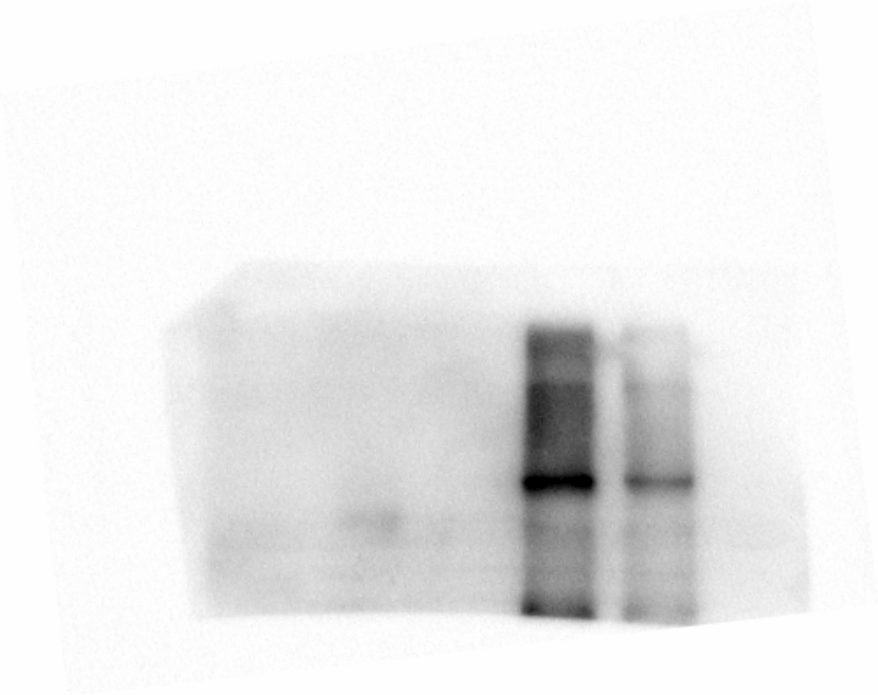

Supplement: Figure 5—source data 2. [file elife-100601-fig5-data2.zip › Figure 5-Source Data 2/Figure5f/IP-WTAP.tif]

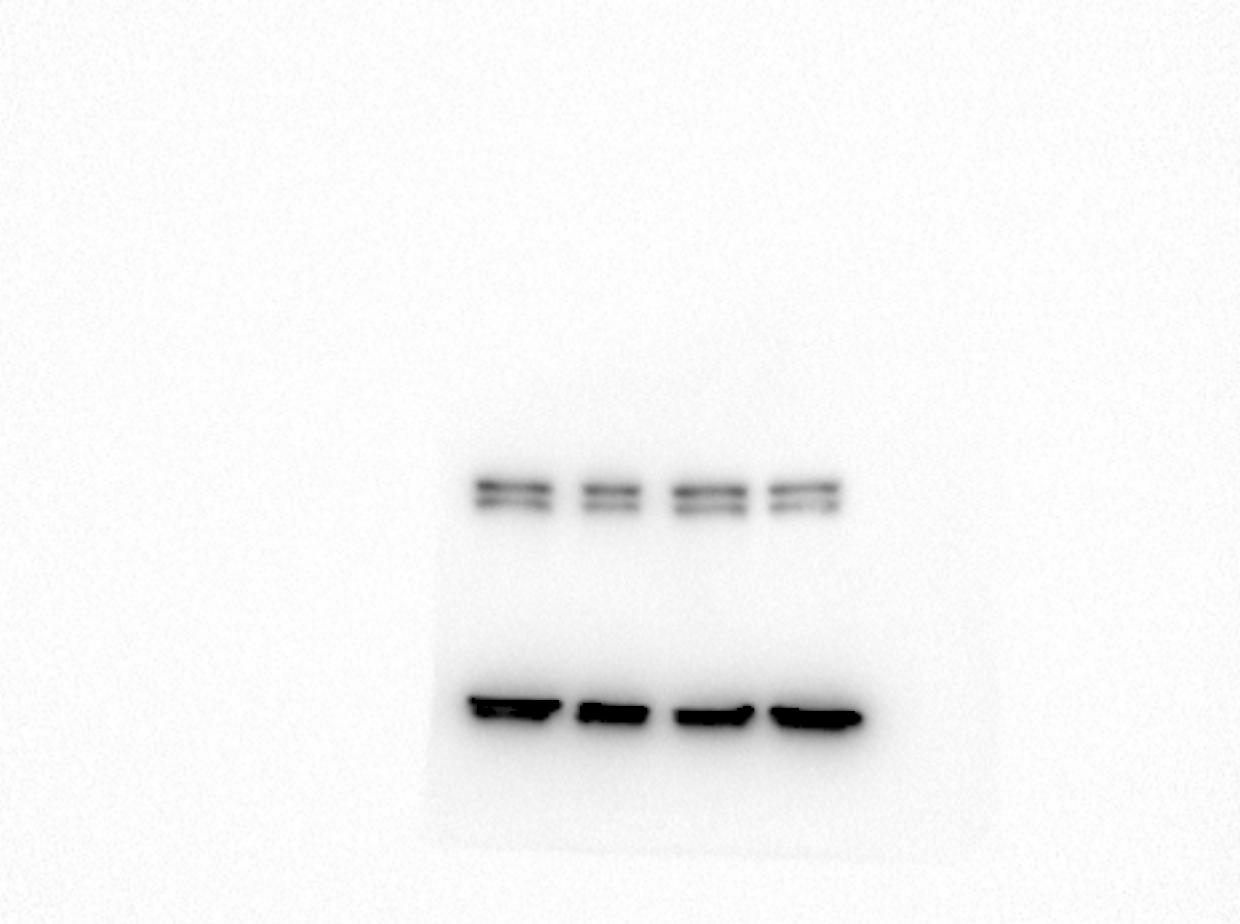

Supplement: Figure 5—figure supplement 1—source data 2. [file elife-100601-fig5-figsupp1-data2.zip › Figure 5-figure supplement 1-Source Data 2/Figure5-figure supplement 1a/actin.tif]

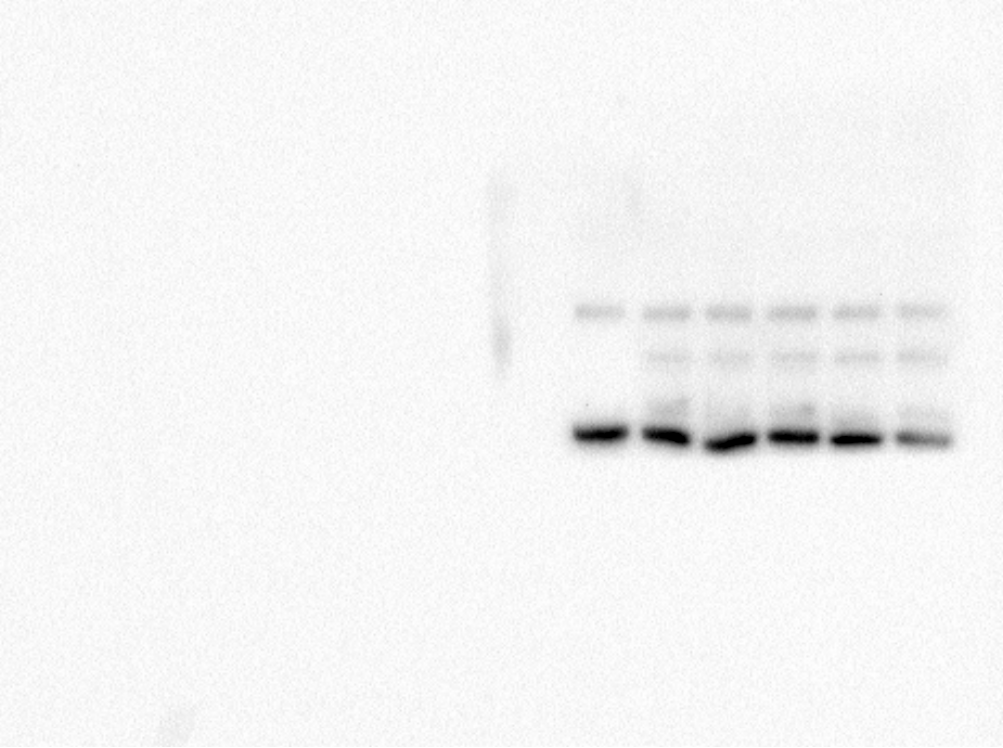

Supplement: Figure 5—figure supplement 1—source data 2. [file elife-100601-fig5-figsupp1-data2.zip › Figure 5-figure supplement 1-Source Data 2/Figure5-figure supplement 1a/METTL3.tif]

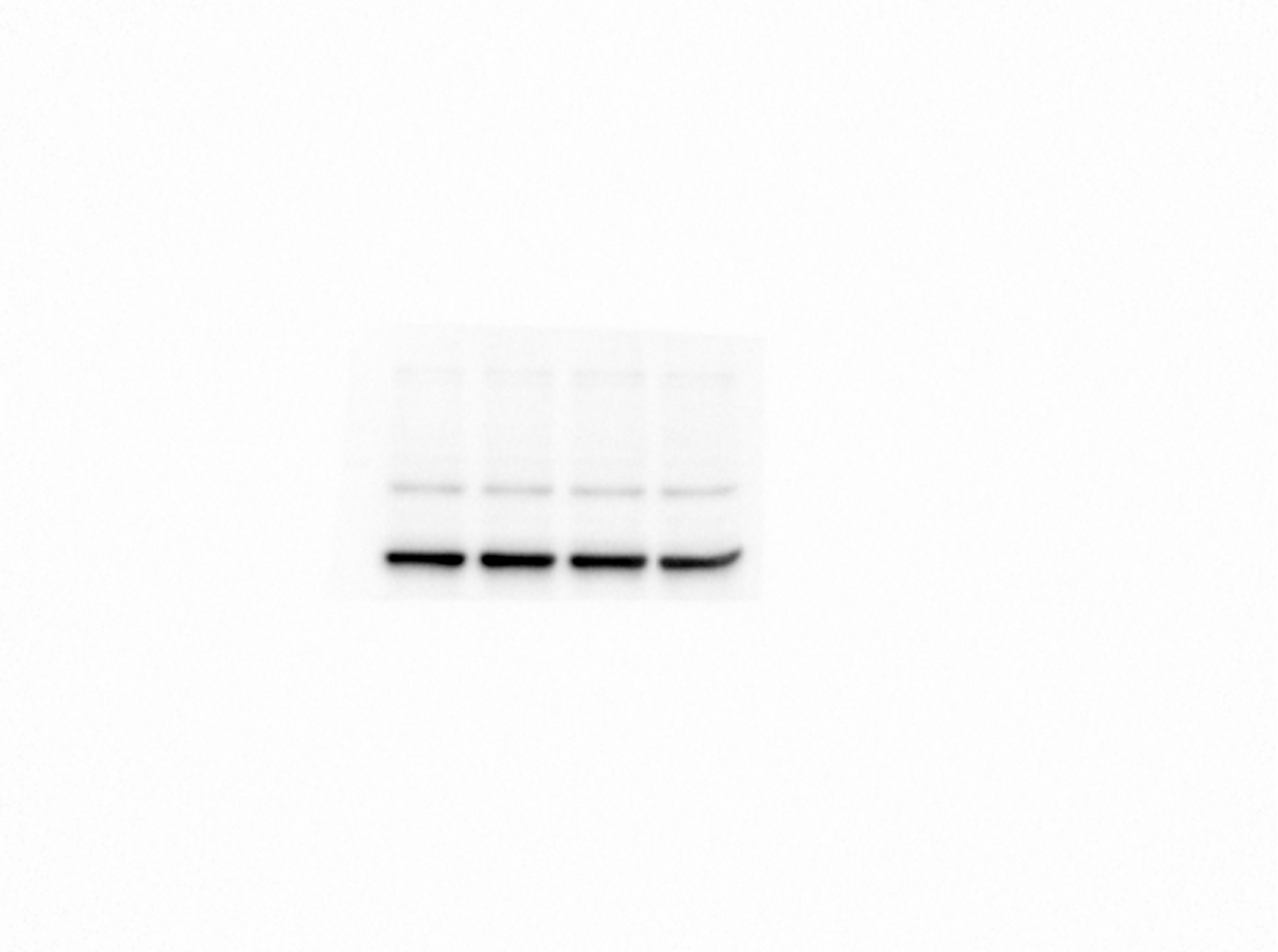

Supplement: Figure 5—figure supplement 1—source data 2. [file elife-100601-fig5-figsupp1-data2.zip › Figure 5-figure supplement 1-Source Data 2/Figure5-figure supplement 1a/METTL14.tif]

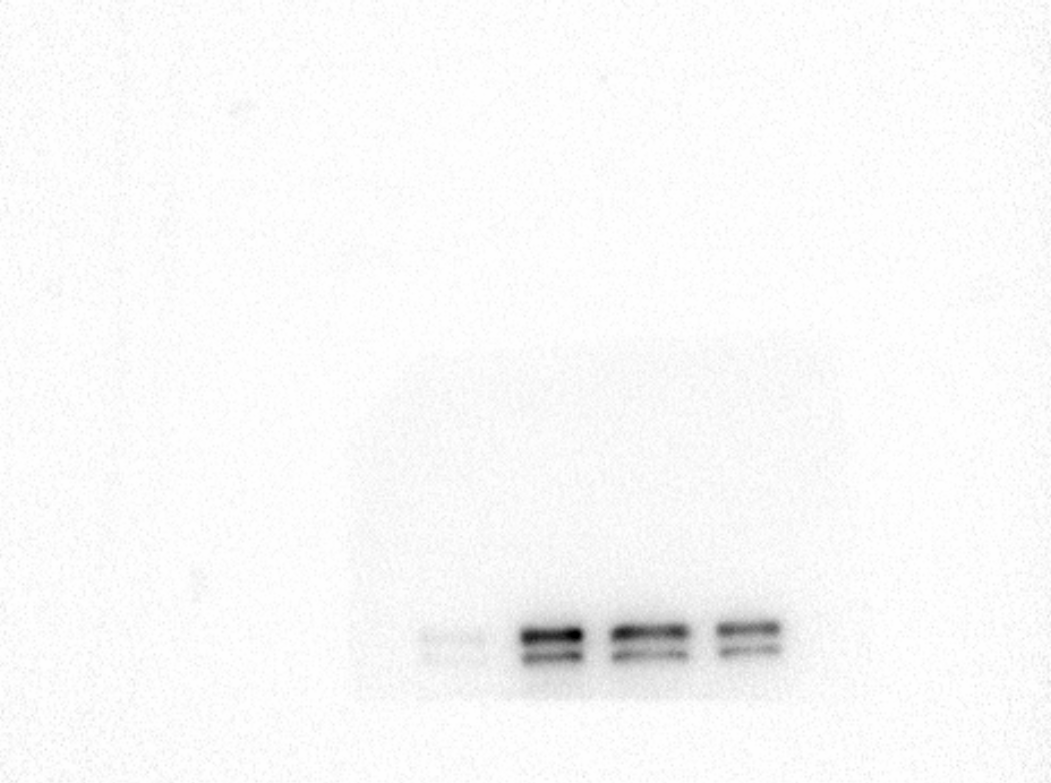

Supplement: Figure 5—figure supplement 1—source data 2. [file elife-100601-fig5-figsupp1-data2.zip › Figure 5-figure supplement 1-Source Data 2/Figure5-figure supplement 1a/pSTAT1.tif]

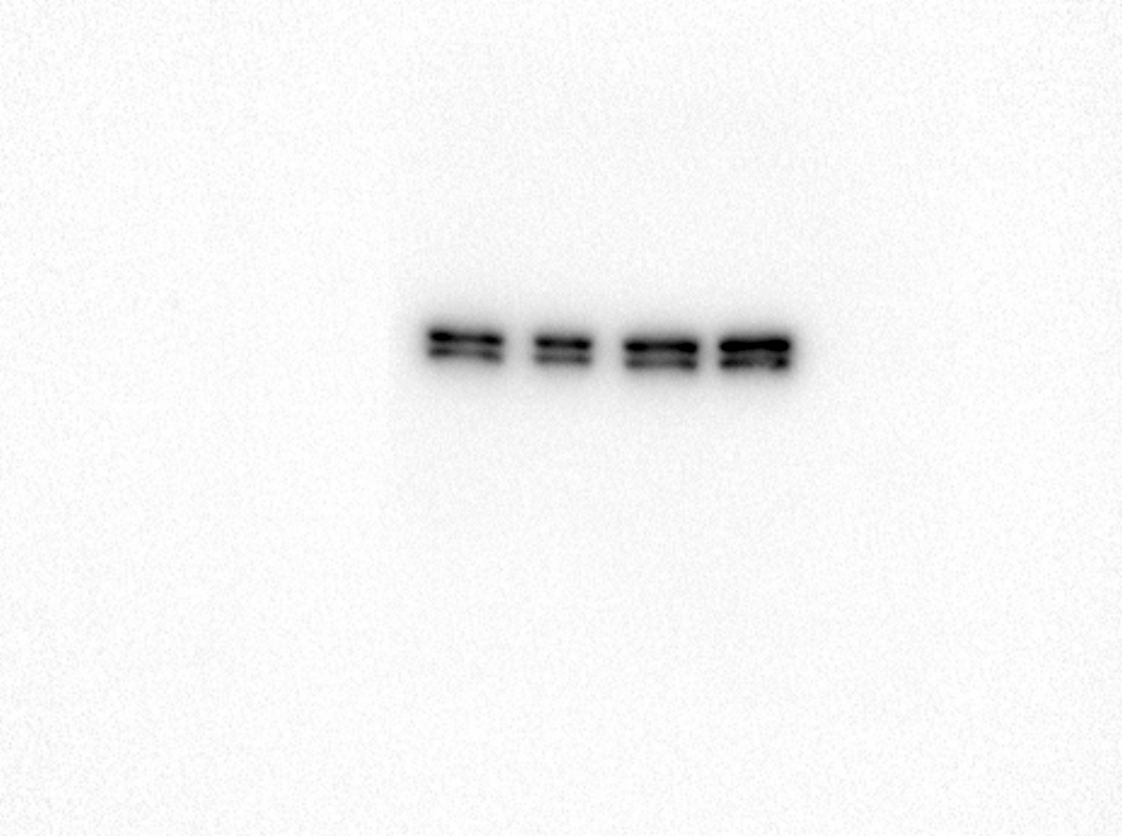

Supplement: Figure 5—figure supplement 1—source data 2. [file elife-100601-fig5-figsupp1-data2.zip › Figure 5-figure supplement 1-Source Data 2/Figure5-figure supplement 1a/STAT1.tif]

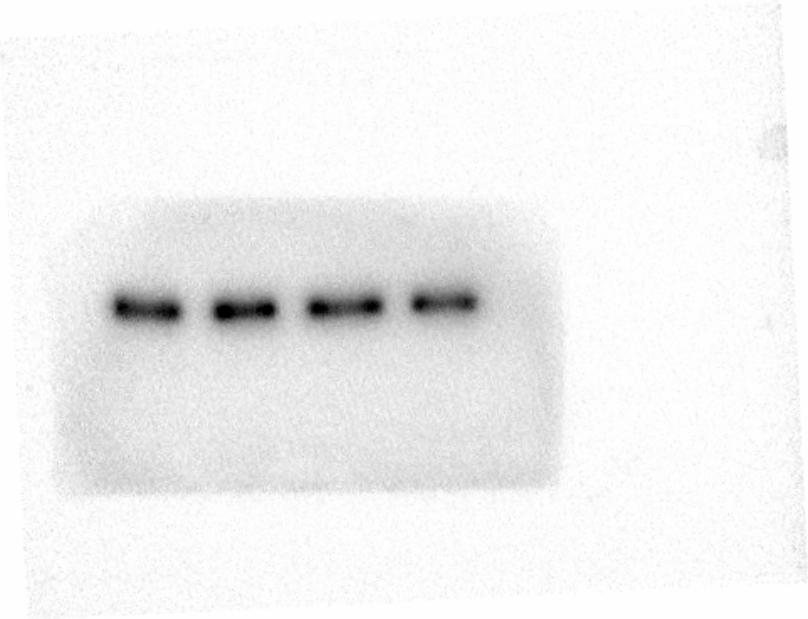

Supplement: Figure 5—figure supplement 1—source data 2. [file elife-100601-fig5-figsupp1-data2.zip › Figure 5-figure supplement 1-Source Data 2/Figure5-figure supplement 1a/WTAP.tif]

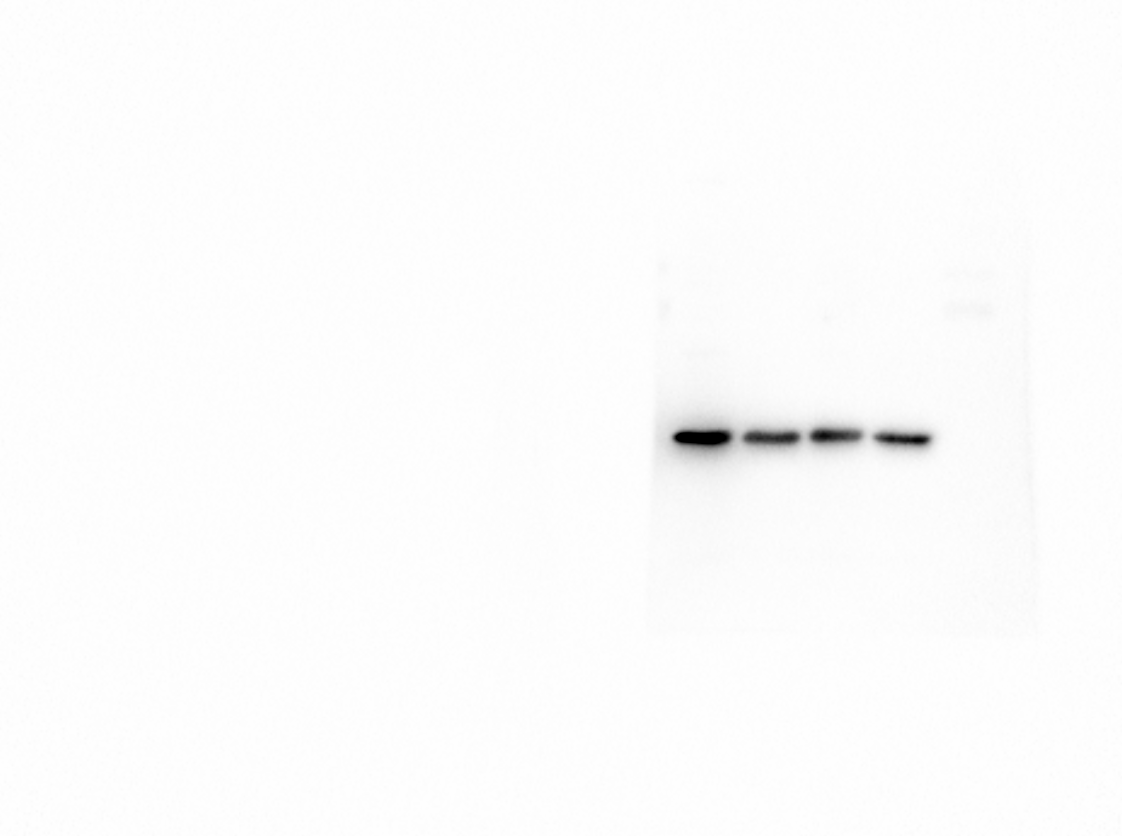

Supplement: Figure 5—figure supplement 1—source data 2. [file elife-100601-fig5-figsupp1-data2.zip › Figure 5-figure supplement 1-Source Data 2/Figure5-figure supplement 1c/actin.tif]

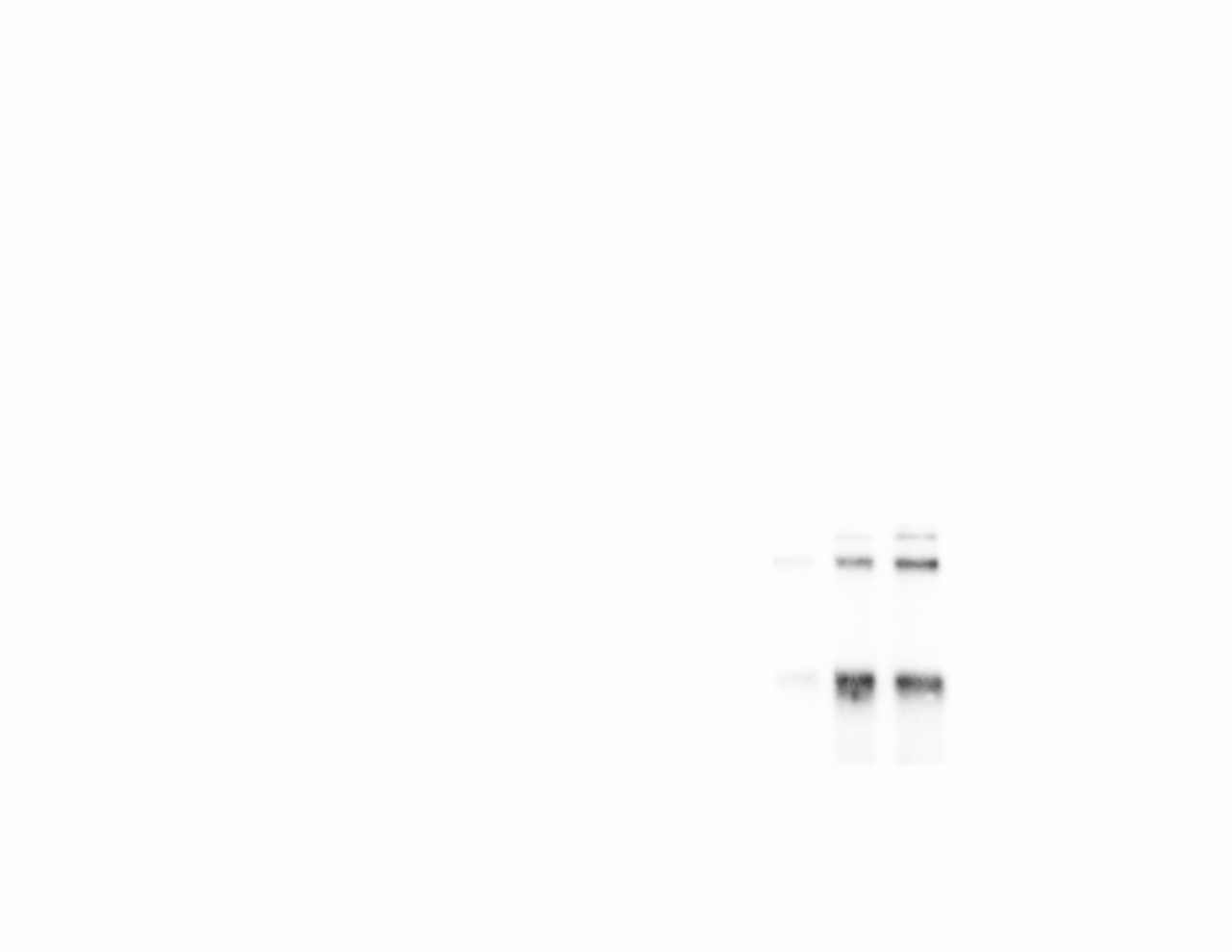

Supplement: Figure 5—figure supplement 1—source data 2. [file elife-100601-fig5-figsupp1-data2.zip › Figure 5-figure supplement 1-Source Data 2/Figure5-figure supplement 1c/IP-pSTAT1.tif]

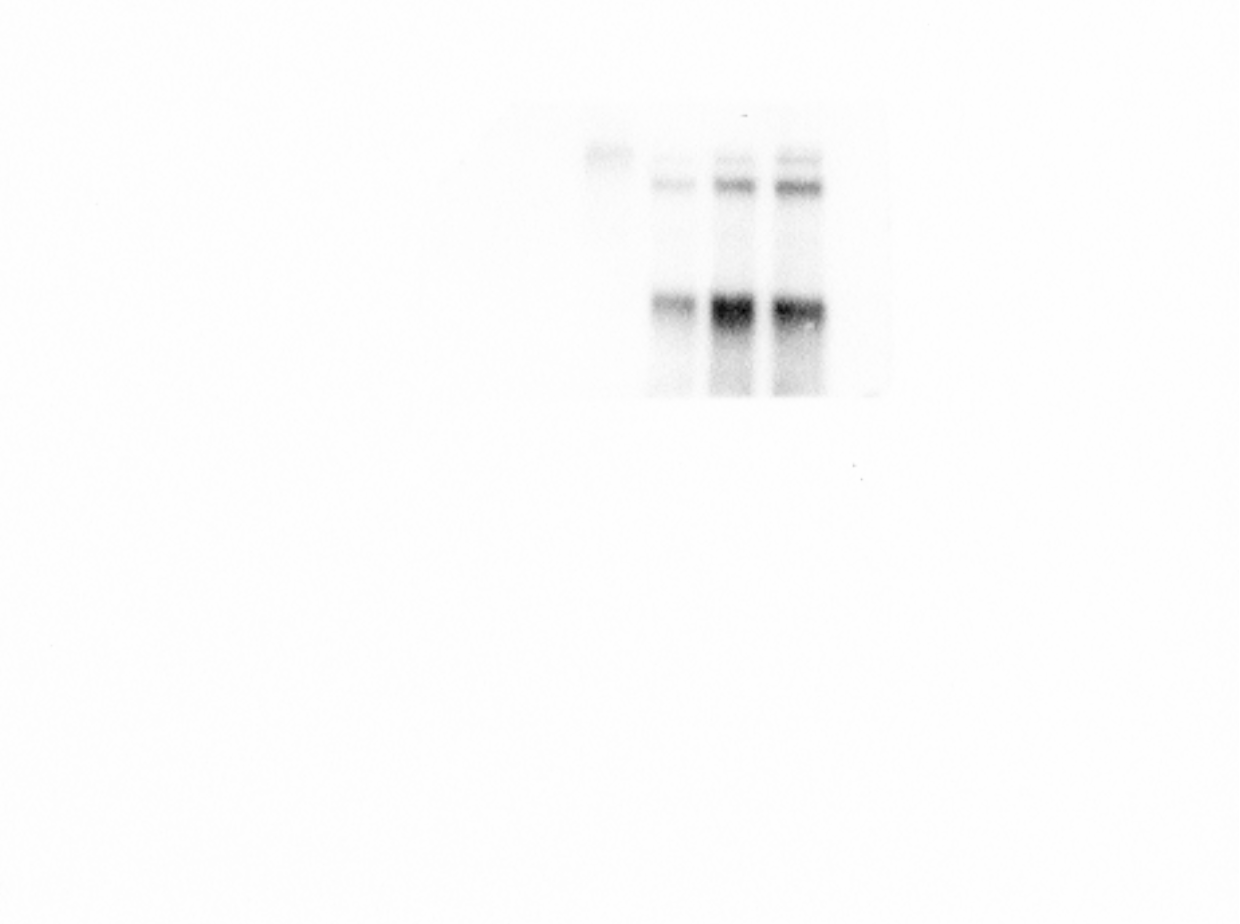

Supplement: Figure 5—figure supplement 1—source data 2. [file elife-100601-fig5-figsupp1-data2.zip › Figure 5-figure supplement 1-Source Data 2/Figure5-figure supplement 1c/IP-STAT1.tif]

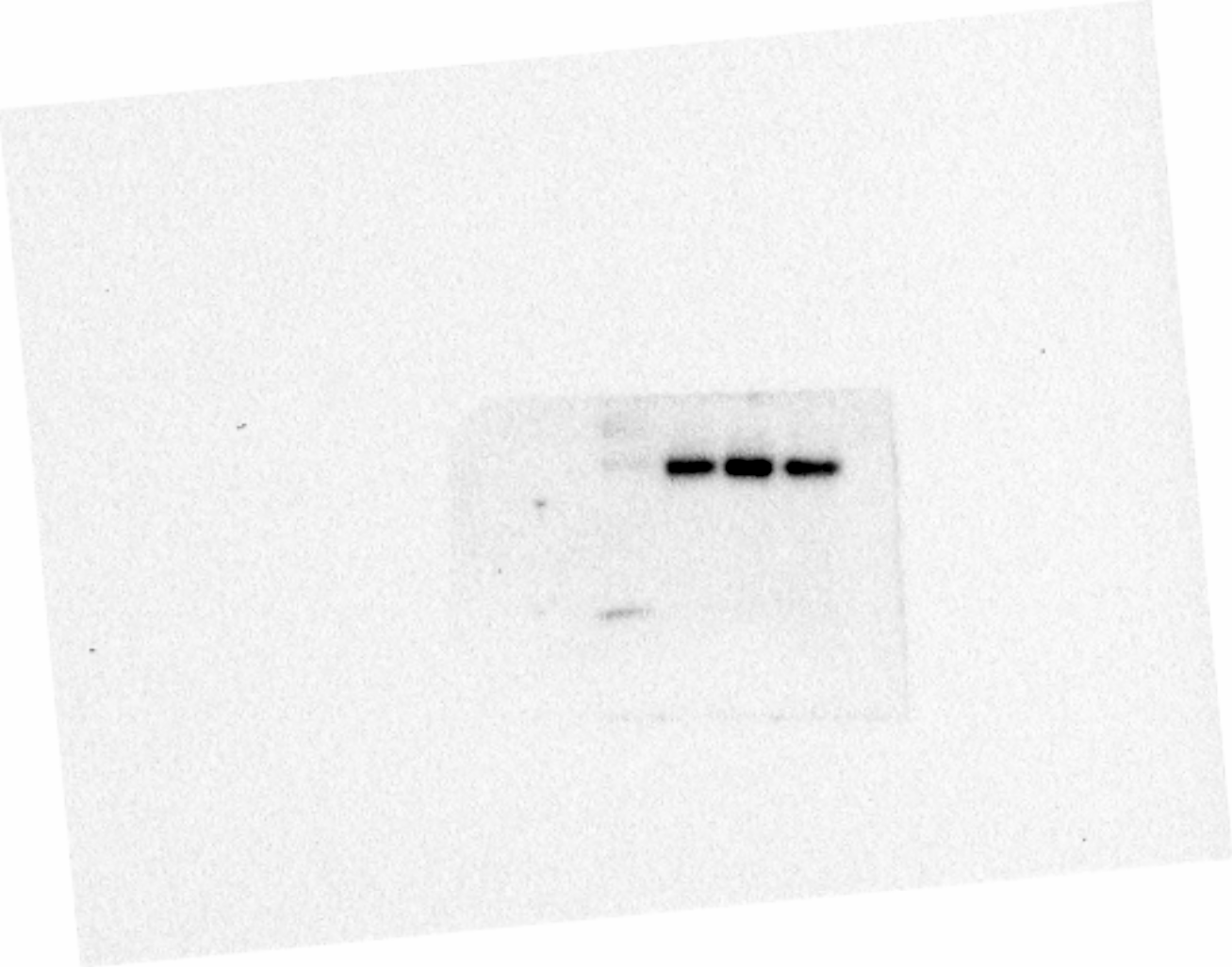

Supplement: Figure 5—figure supplement 1—source data 2. [file elife-100601-fig5-figsupp1-data2.zip › Figure 5-figure supplement 1-Source Data 2/Figure5-figure supplement 1c/IP-WTAP.tif]

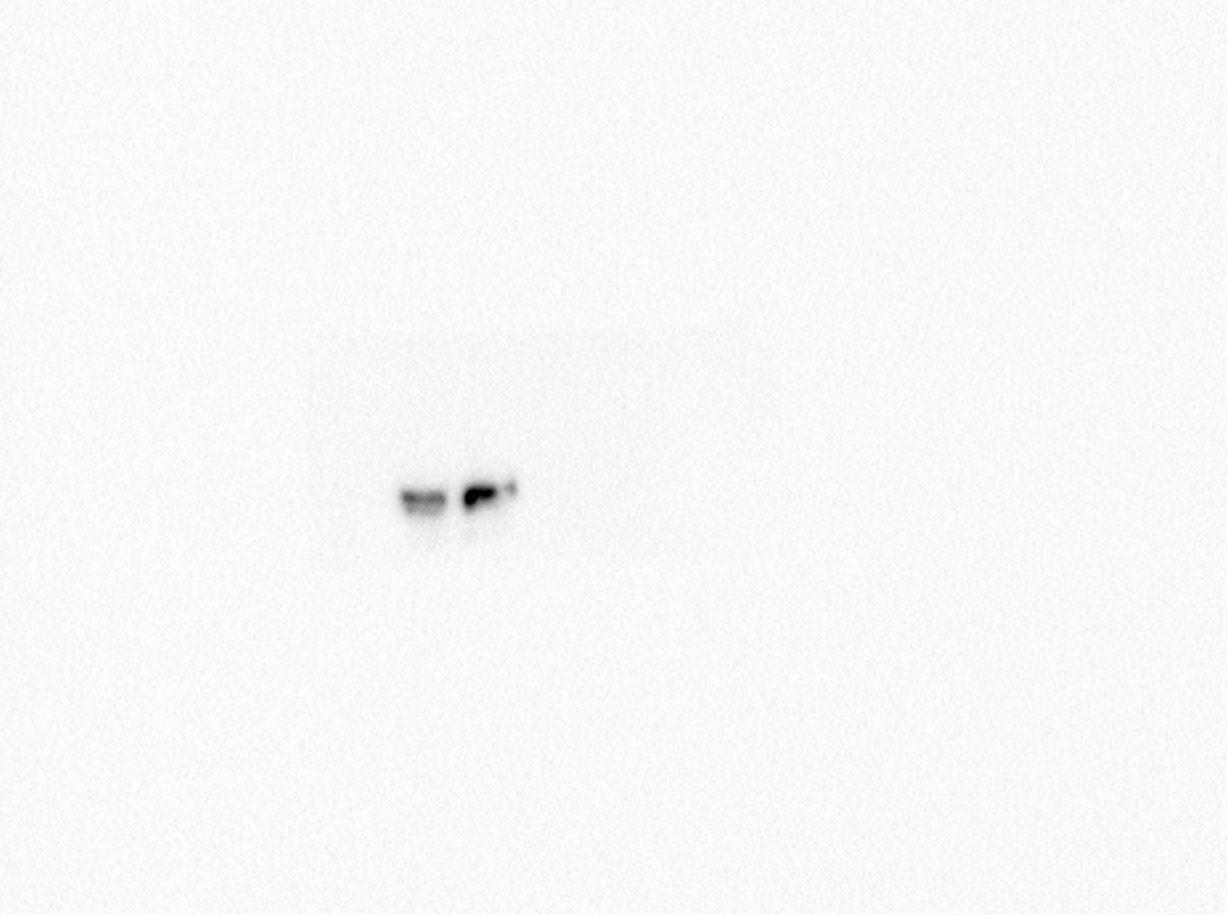

Supplement: Figure 5—figure supplement 1—source data 2. [file elife-100601-fig5-figsupp1-data2.zip › Figure 5-figure supplement 1-Source Data 2/Figure5-figure supplement 1c/pSTAT1.tif]

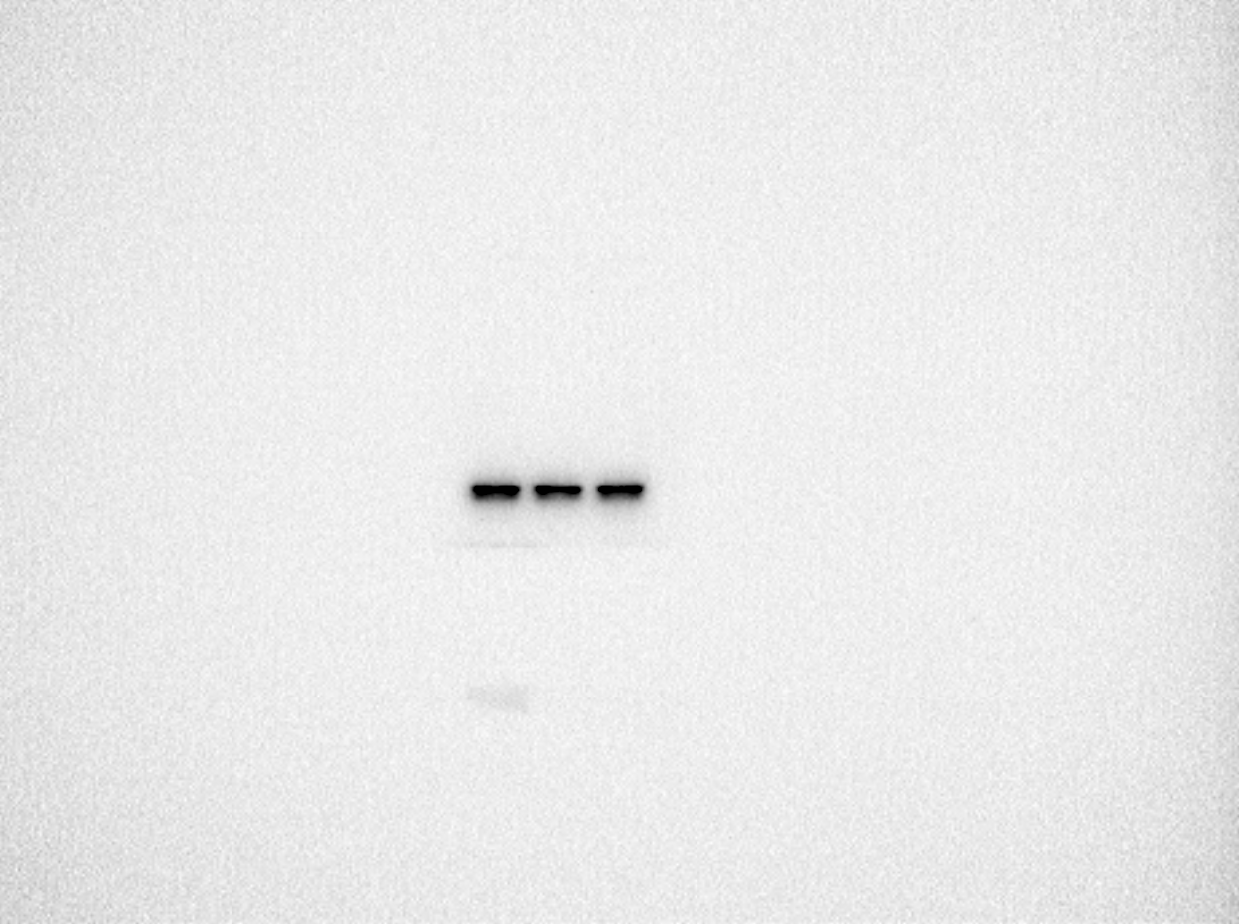

Supplement: Figure 5—figure supplement 1—source data 2. [file elife-100601-fig5-figsupp1-data2.zip › Figure 5-figure supplement 1-Source Data 2/Figure5-figure supplement 1c/STAT1.tif]

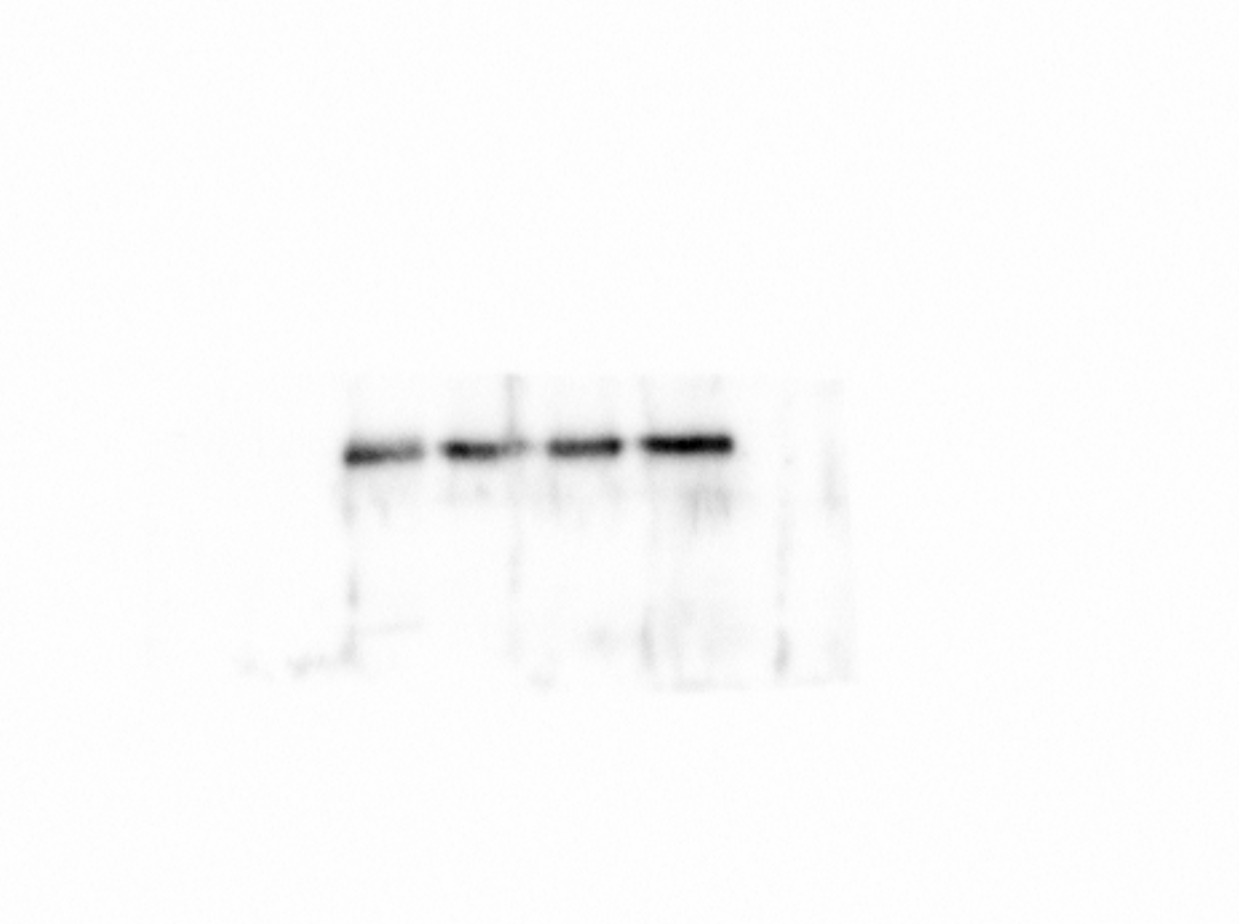

Supplement: Figure 5—figure supplement 1—source data 2. [file elife-100601-fig5-figsupp1-data2.zip › Figure 5-figure supplement 1-Source Data 2/Figure5-figure supplement 1c/WTAP.tif]

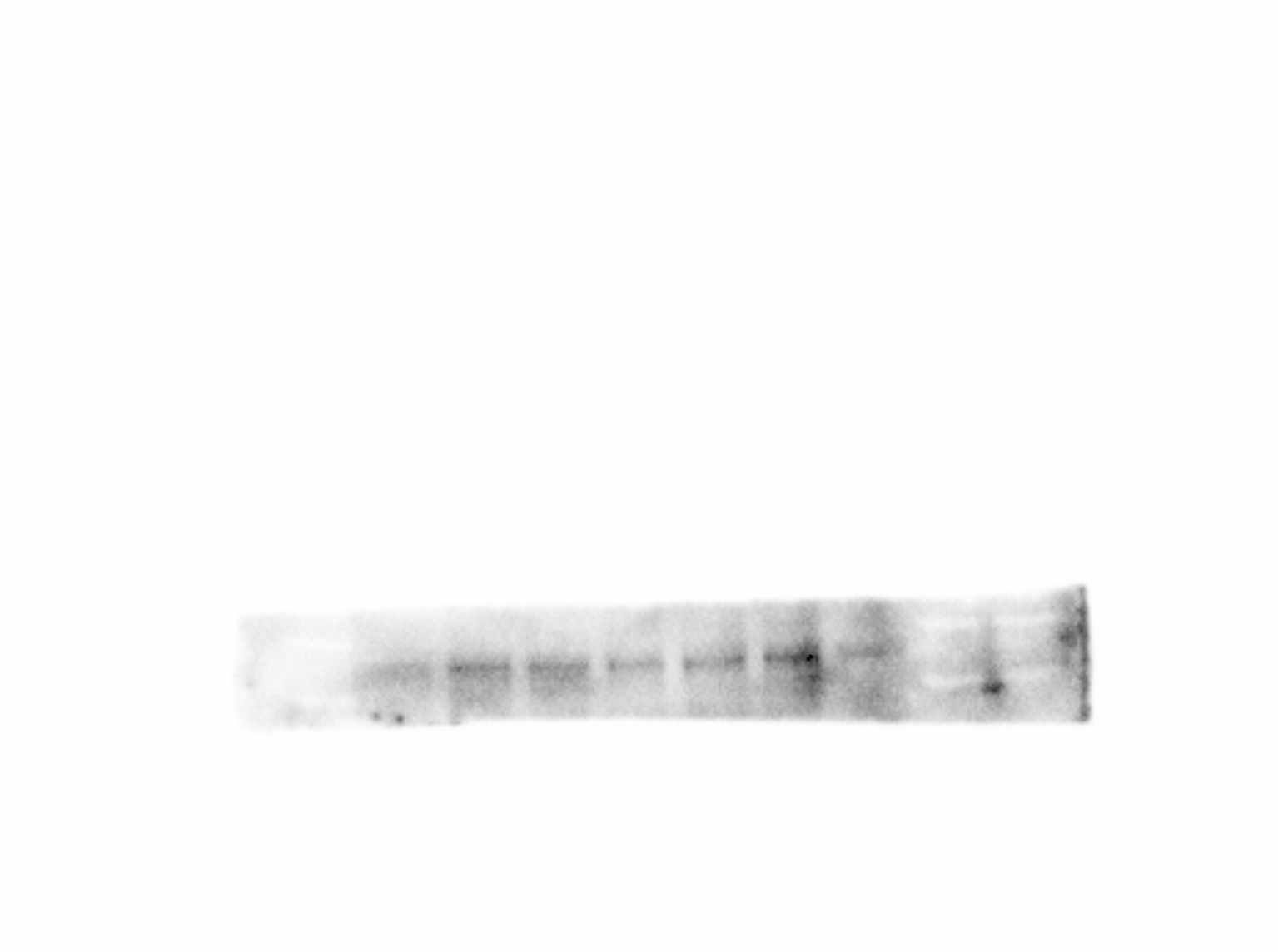

Supplement: Figure 5—figure supplement 1—source data 2. [file elife-100601-fig5-figsupp1-data2.zip › Figure 5-figure supplement 1-Source Data 2/Figure5-figure supplement 1d/IP-METTL3.tif]

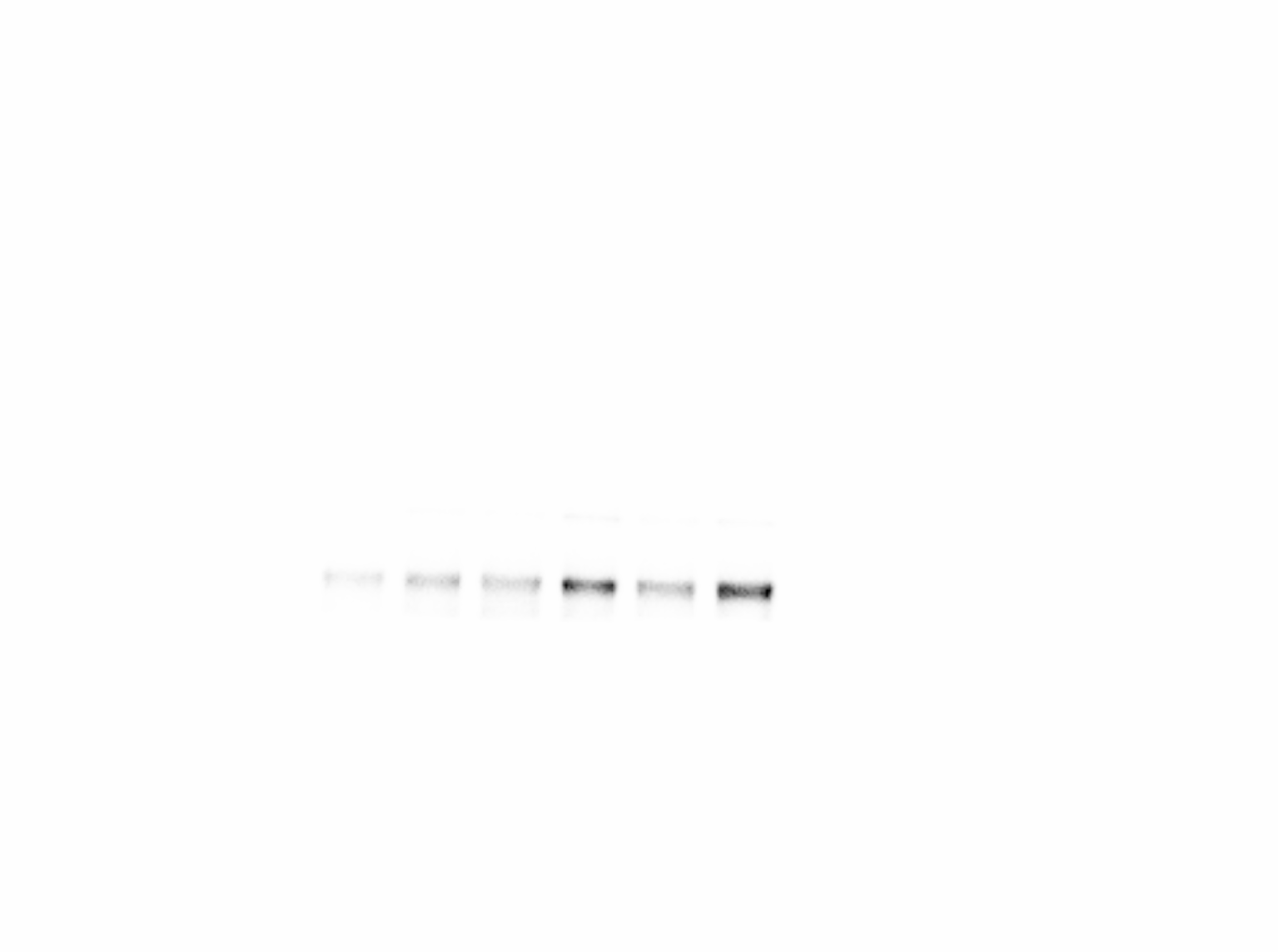

Supplement: Figure 5—figure supplement 1—source data 2. [file elife-100601-fig5-figsupp1-data2.zip › Figure 5-figure supplement 1-Source Data 2/Figure5-figure supplement 1d/IP-STAT1.tif]

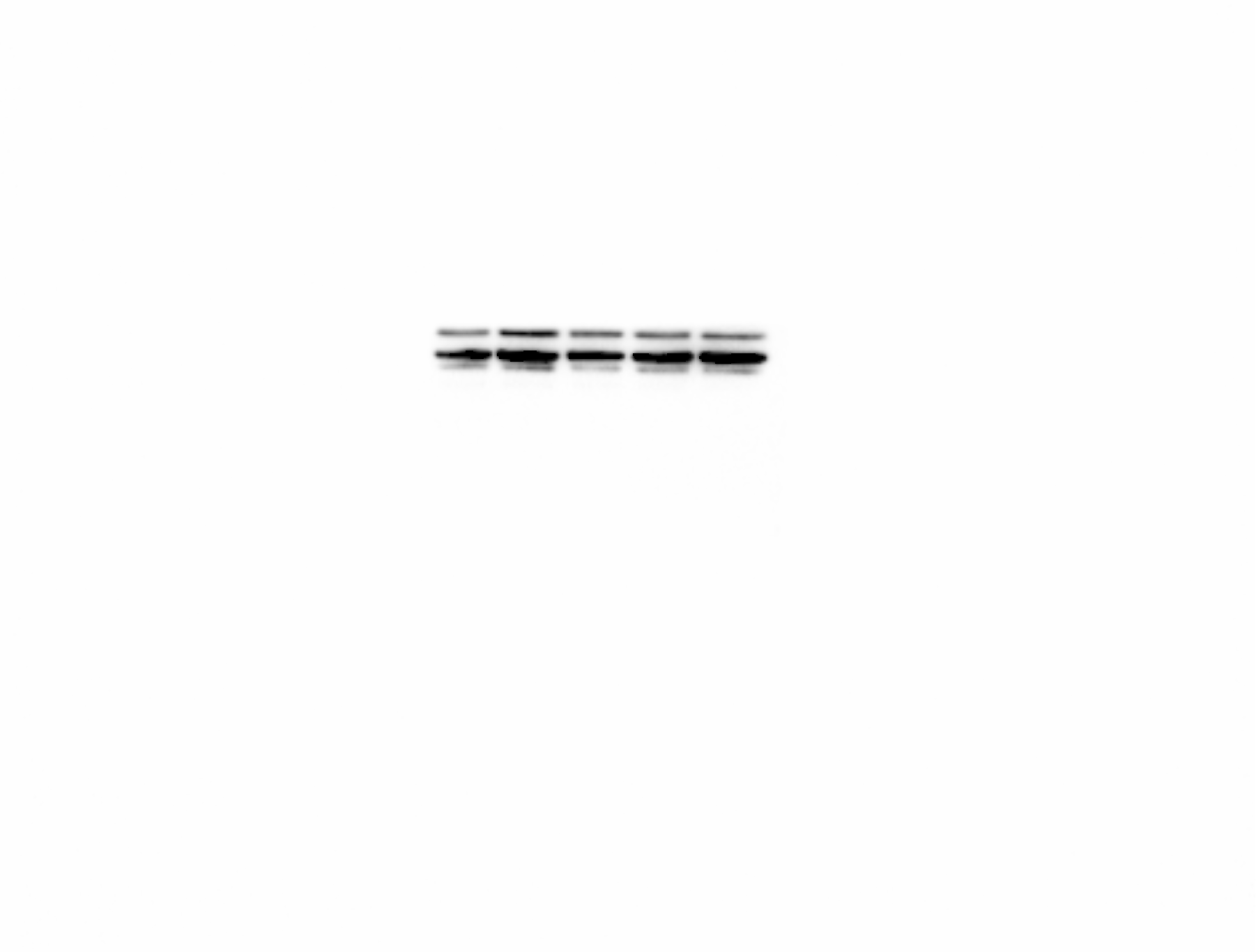

Supplement: Figure 5—figure supplement 1—source data 2. [file elife-100601-fig5-figsupp1-data2.zip › Figure 5-figure supplement 1-Source Data 2/Figure5-figure supplement 1d/actin.tif]

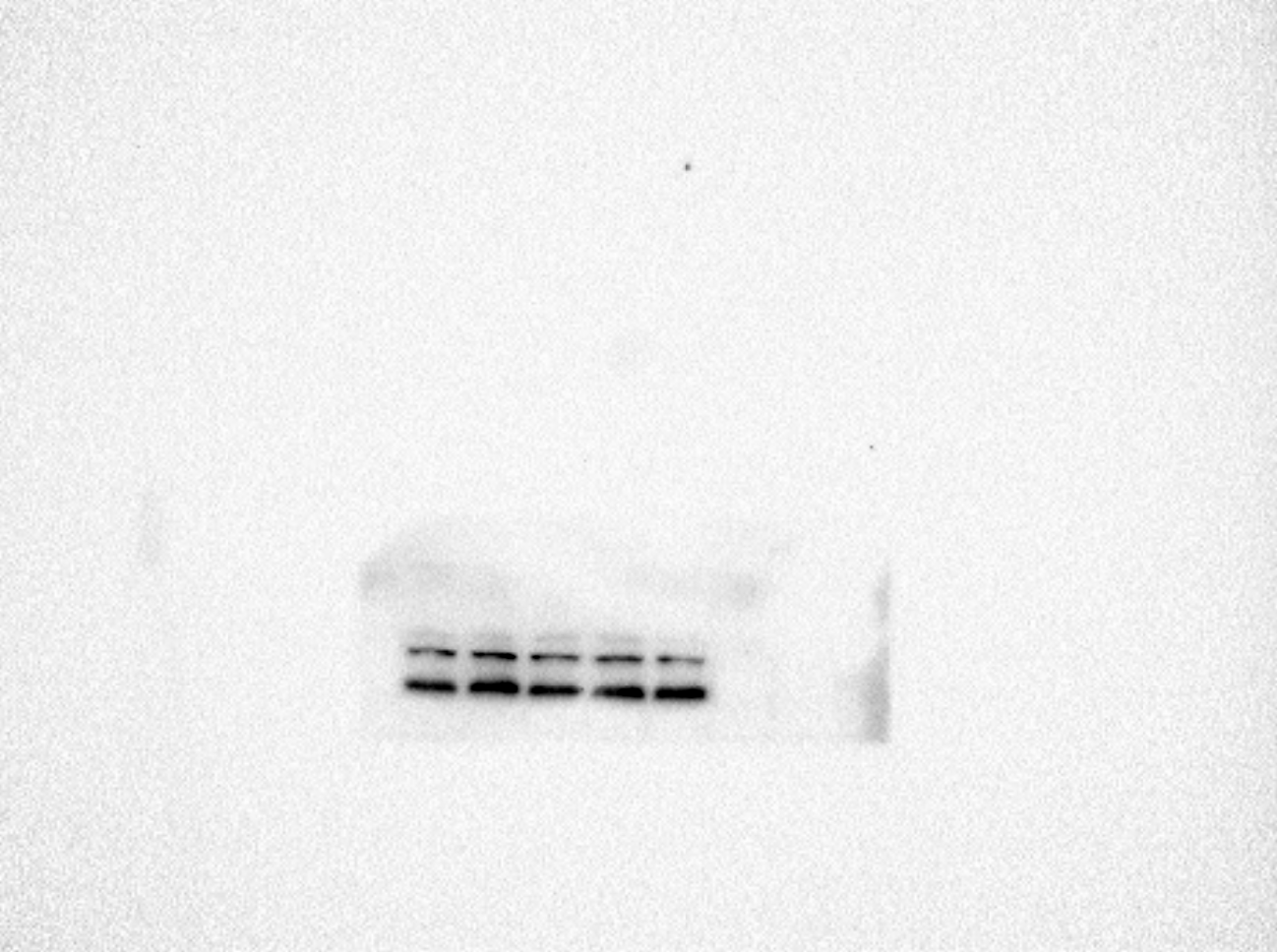

Supplement: Figure 5—figure supplement 1—source data 2. [file elife-100601-fig5-figsupp1-data2.zip › Figure 5-figure supplement 1-Source Data 2/Figure5-figure supplement 1d/METTL3.tif]

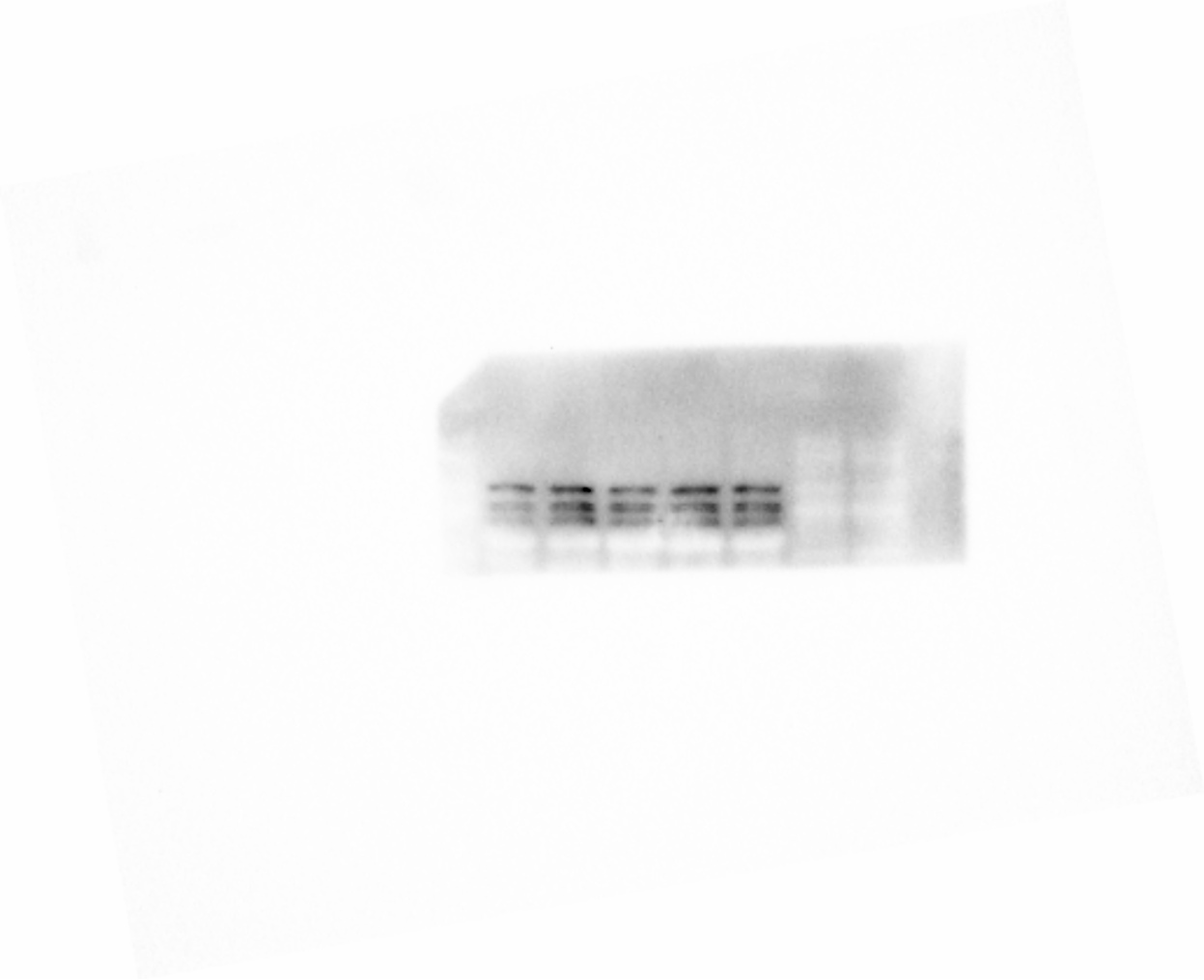

Supplement: Figure 5—figure supplement 1—source data 2. [file elife-100601-fig5-figsupp1-data2.zip › Figure 5-figure supplement 1-Source Data 2/Figure5-figure supplement 1d/STAT1.tif]

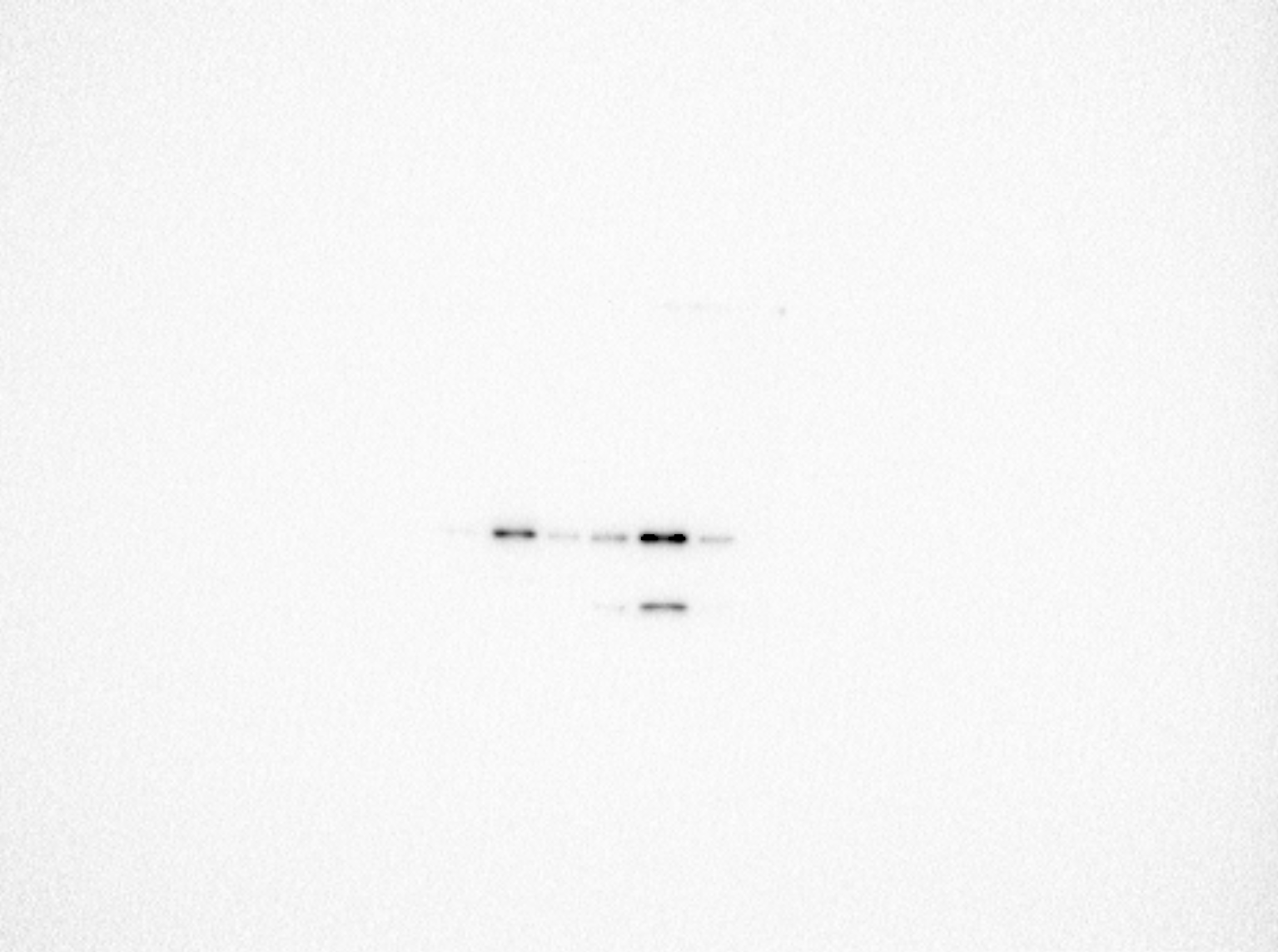

Supplement: Figure 5—figure supplement 1—source data 2. [file elife-100601-fig5-figsupp1-data2.zip › Figure 5-figure supplement 1-Source Data 2/Figure5-figure supplement 1e/IP-METTL3.tif]

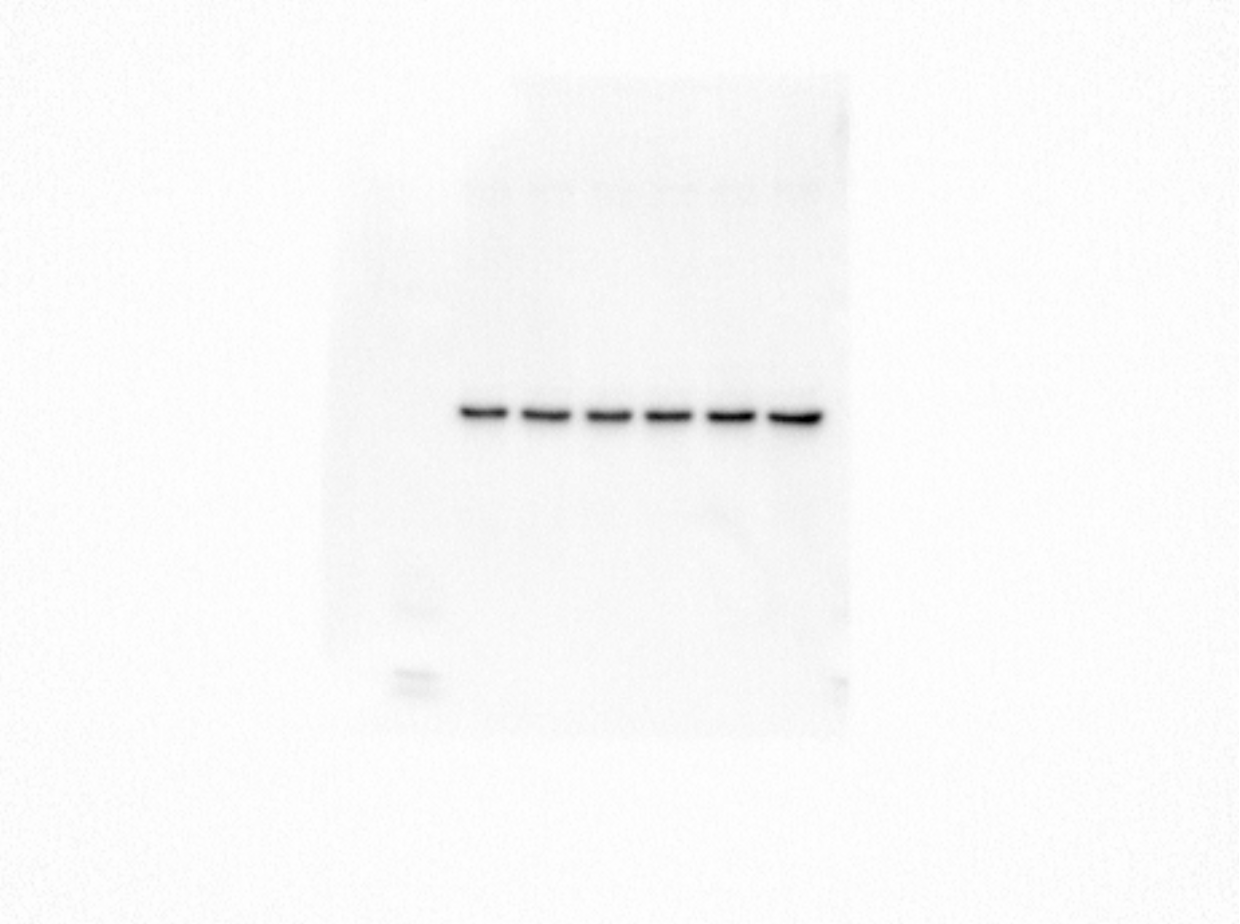

Supplement: Figure 5—figure supplement 1—source data 2. [file elife-100601-fig5-figsupp1-data2.zip › Figure 5-figure supplement 1-Source Data 2/Figure5-figure supplement 1e/IP-STAT1.tif]

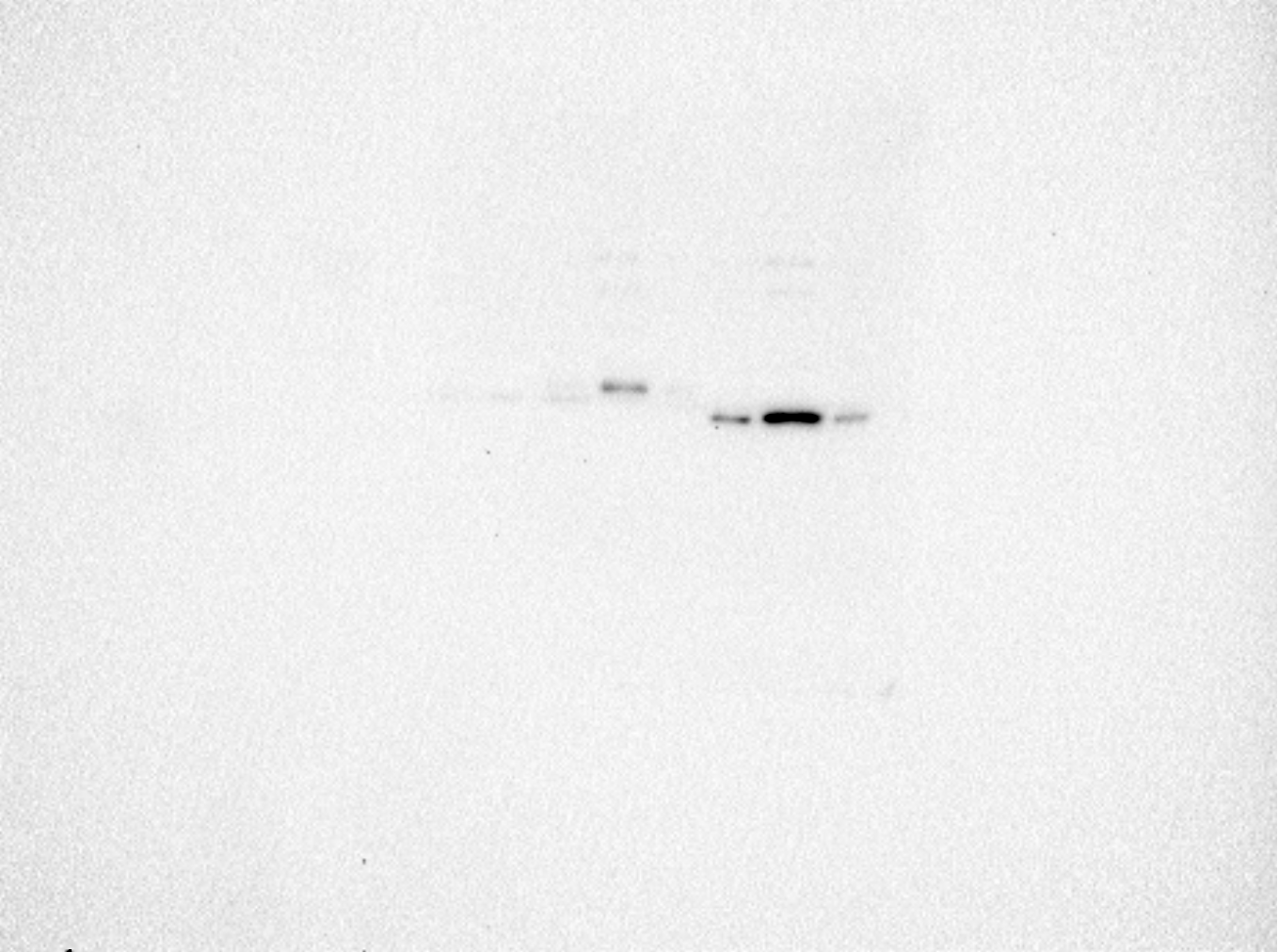

Supplement: Figure 5—figure supplement 1—source data 2. [file elife-100601-fig5-figsupp1-data2.zip › Figure 5-figure supplement 1-Source Data 2/Figure5-figure supplement 1e/IP-WTAP.tif]

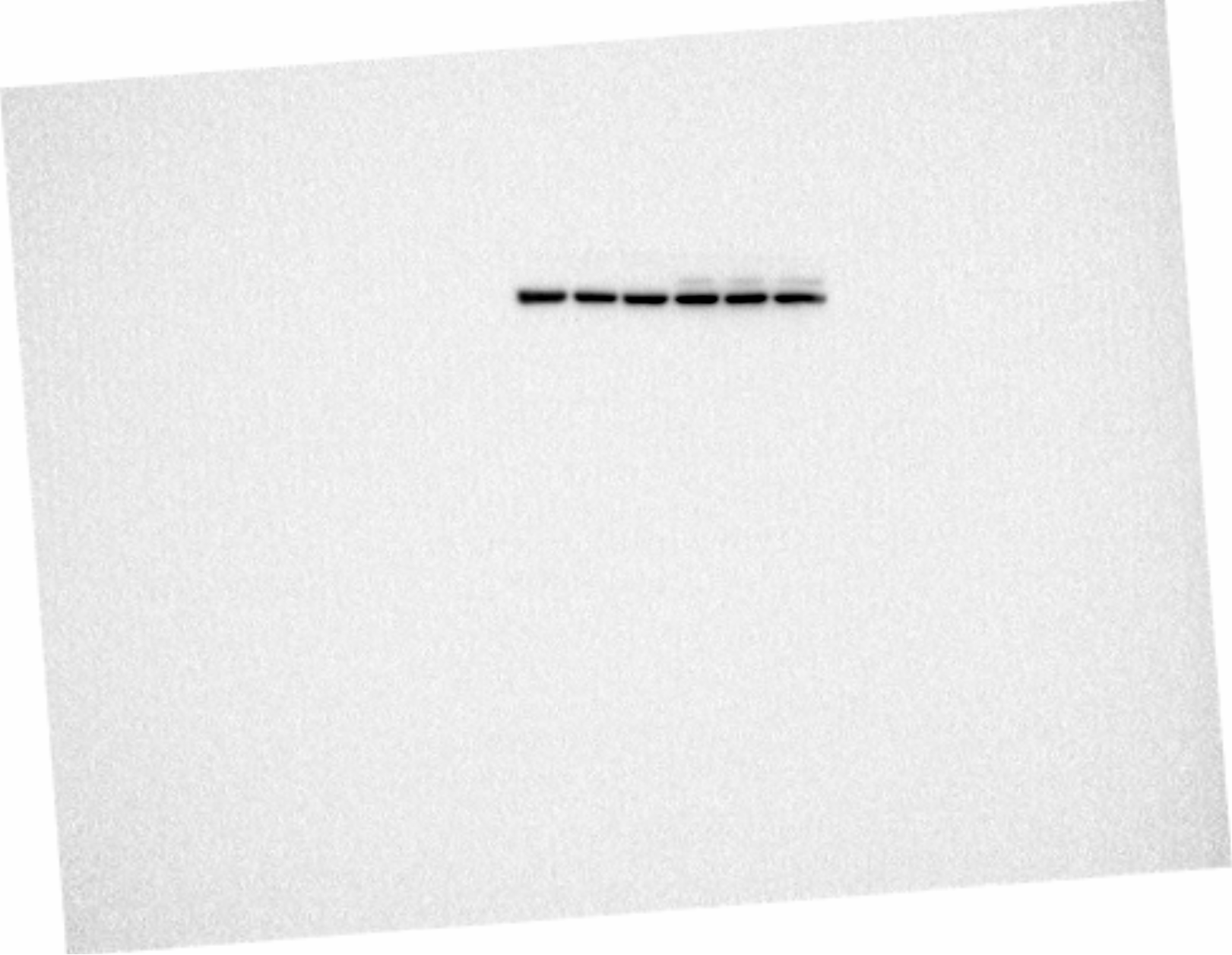

Supplement: Figure 5—figure supplement 1—source data 2. [file elife-100601-fig5-figsupp1-data2.zip › Figure 5-figure supplement 1-Source Data 2/Figure5-figure supplement 1e/actin.tif]

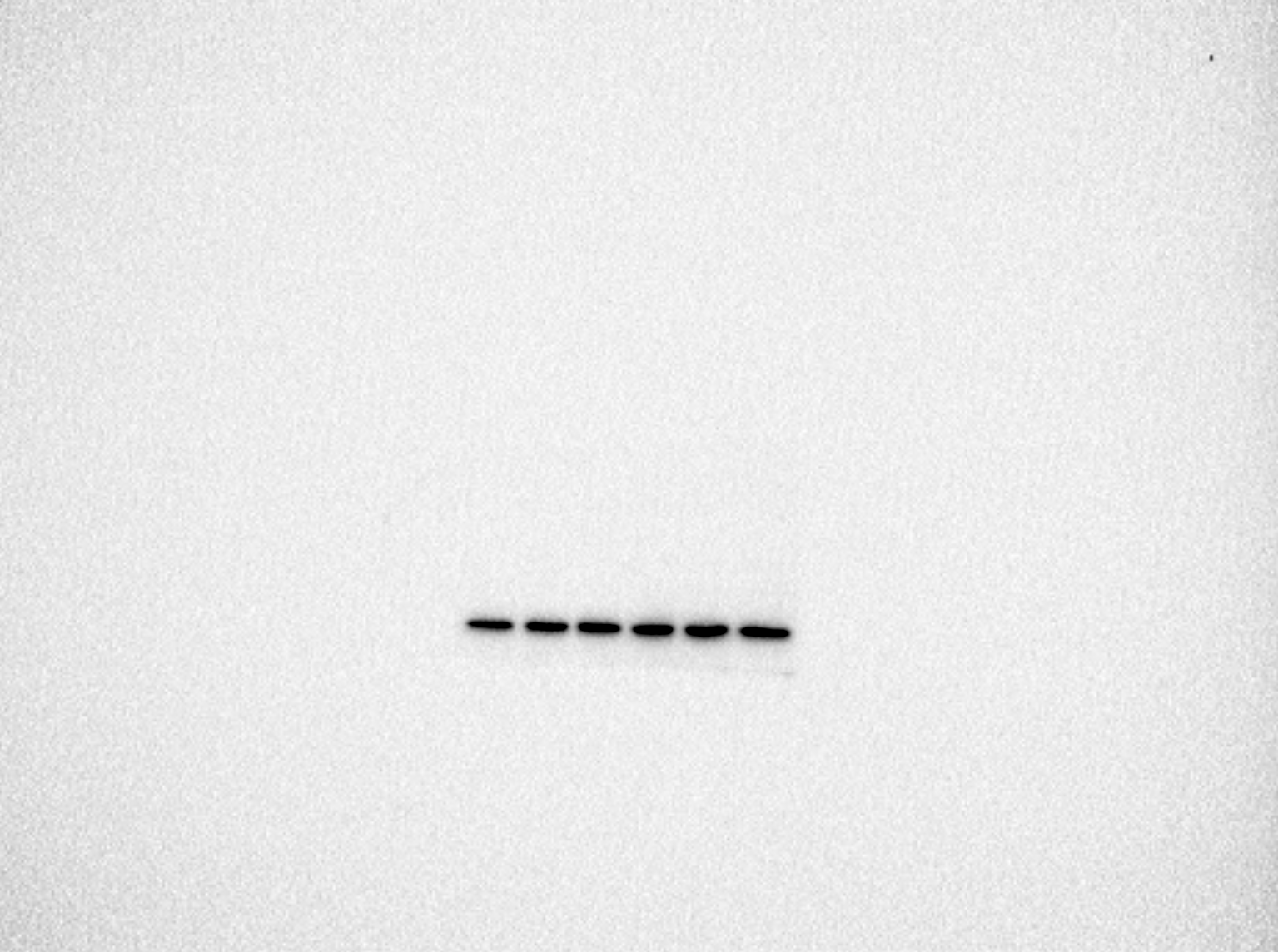

Supplement: Figure 5—figure supplement 1—source data 2. [file elife-100601-fig5-figsupp1-data2.zip › Figure 5-figure supplement 1-Source Data 2/Figure5-figure supplement 1e/METTL3.tif]

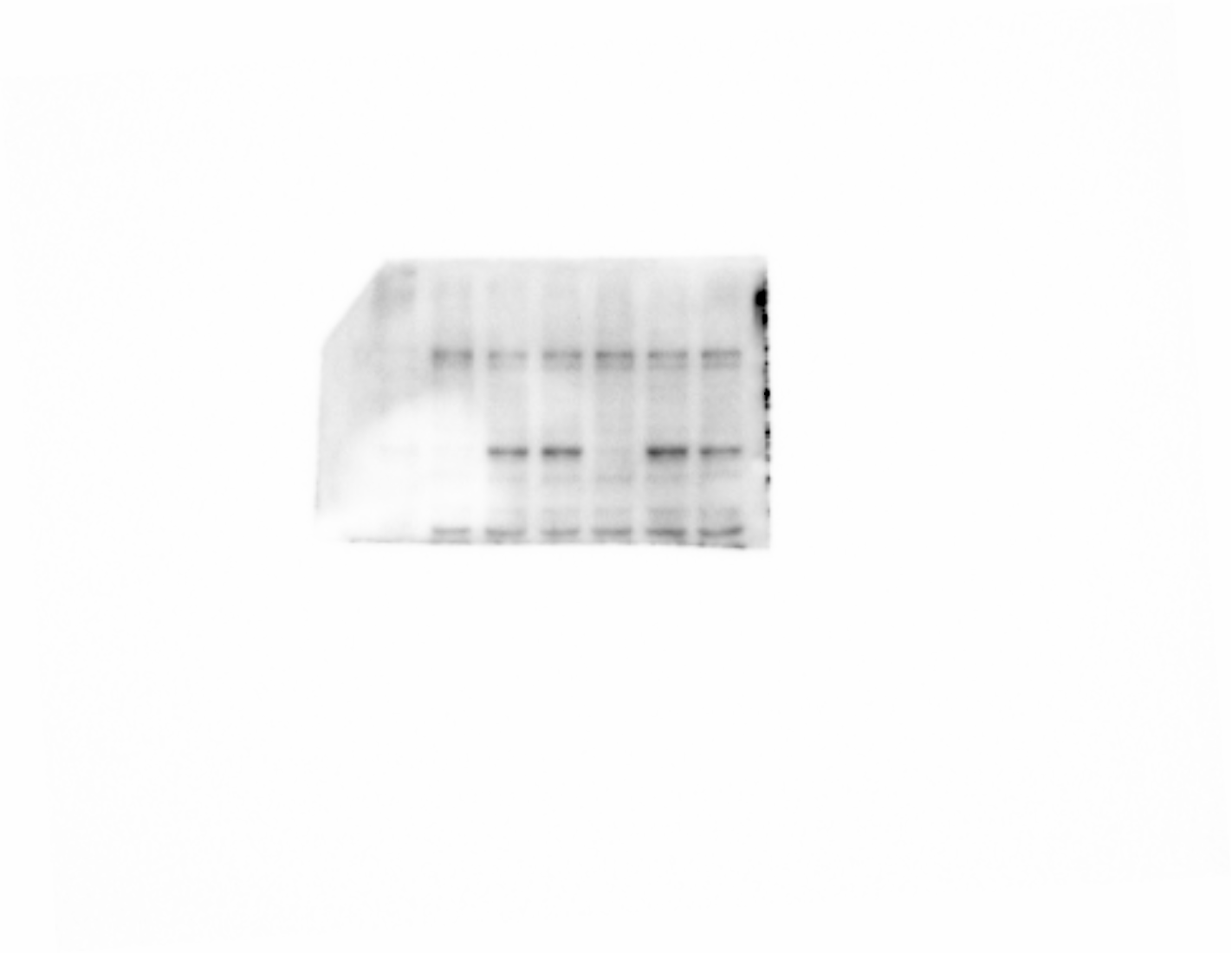

Supplement: Figure 5—figure supplement 1—source data 2. [file elife-100601-fig5-figsupp1-data2.zip › Figure 5-figure supplement 1-Source Data 2/Figure5-figure supplement 1e/pSTAT1.tif]

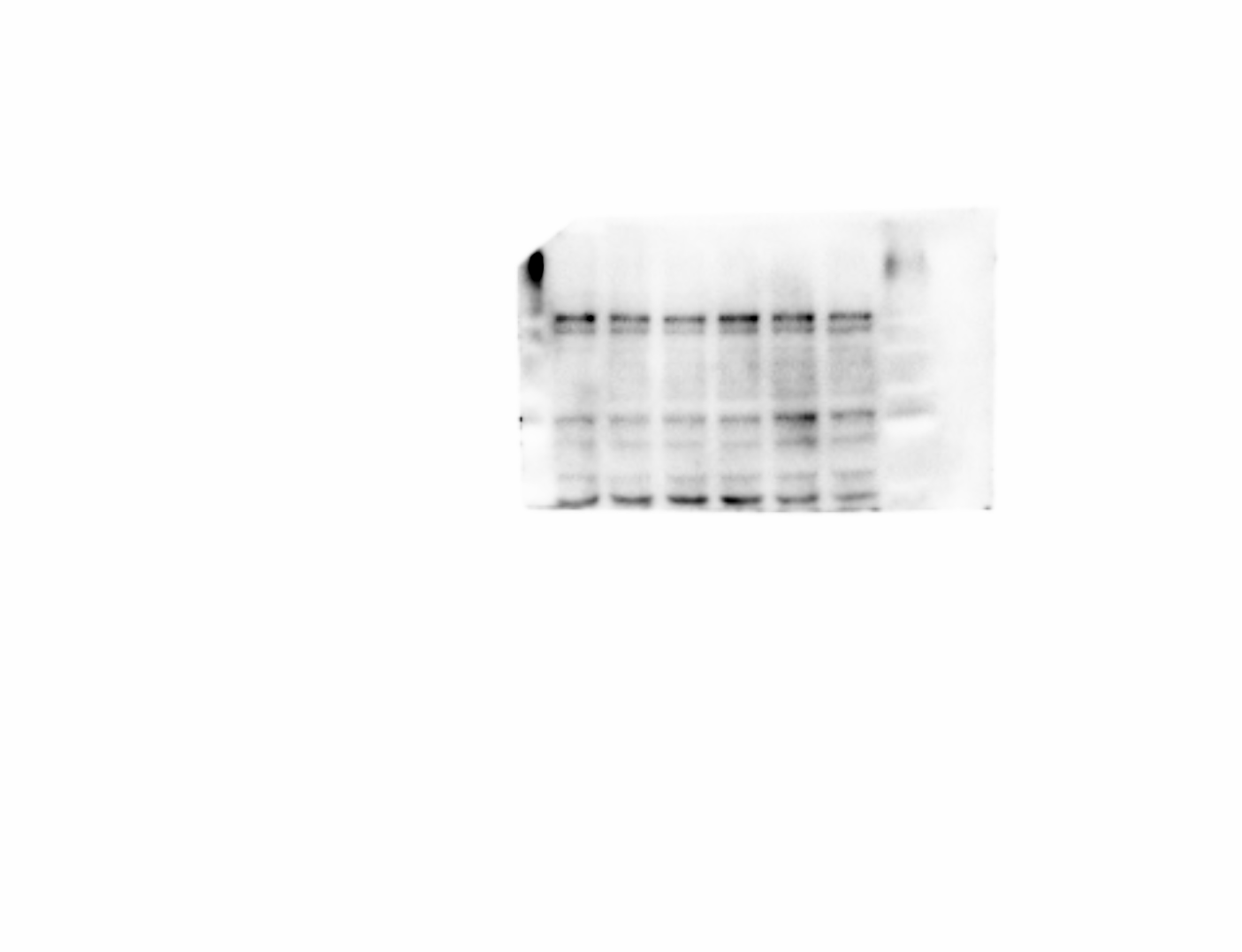

Supplement: Figure 5—figure supplement 1—source data 2. [file elife-100601-fig5-figsupp1-data2.zip › Figure 5-figure supplement 1-Source Data 2/Figure5-figure supplement 1e/STAT1.tif]

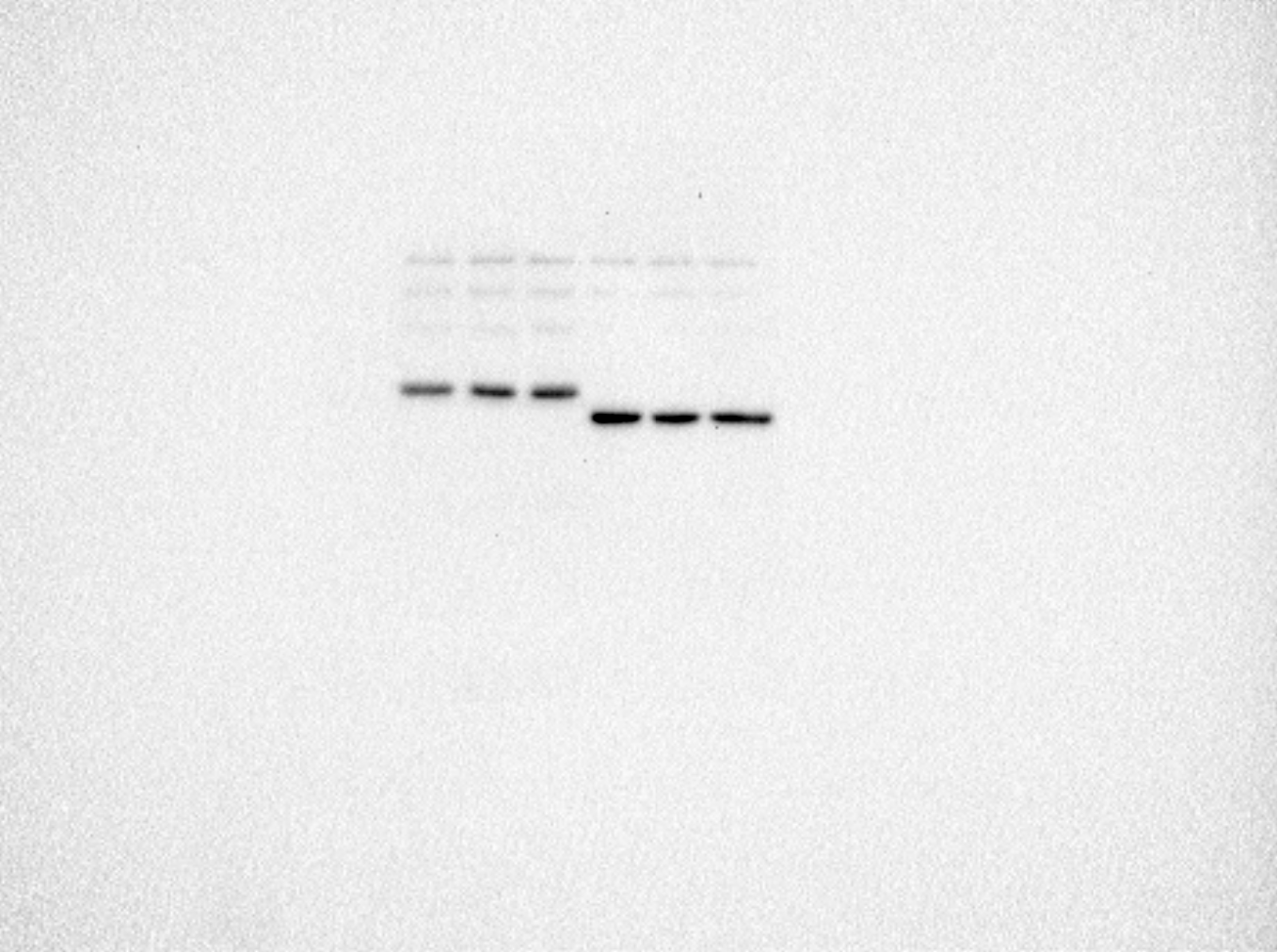

Supplement: Figure 5—figure supplement 1—source data 2. [file elife-100601-fig5-figsupp1-data2.zip › Figure 5-figure supplement 1-Source Data 2/Figure5-figure supplement 1e/WTAP.tif]
