# Supplementary material for: Phase transition of WTAP regulates m6A modification of interferon-stimulated genes
Source: eLife. 2025 May 27;13:RP100601. doi: 10.7554/eLife.100601 (PMC12113268; doi:10.7554/eLife.100601)
Supplement: Figure 2—figure supplement 1—source data 1. [file elife-100601-fig2-figsupp1-data1.zip › Figure 2-figure supplement1-Source Data 1/Figure2-figure supplement 1B.pdf]

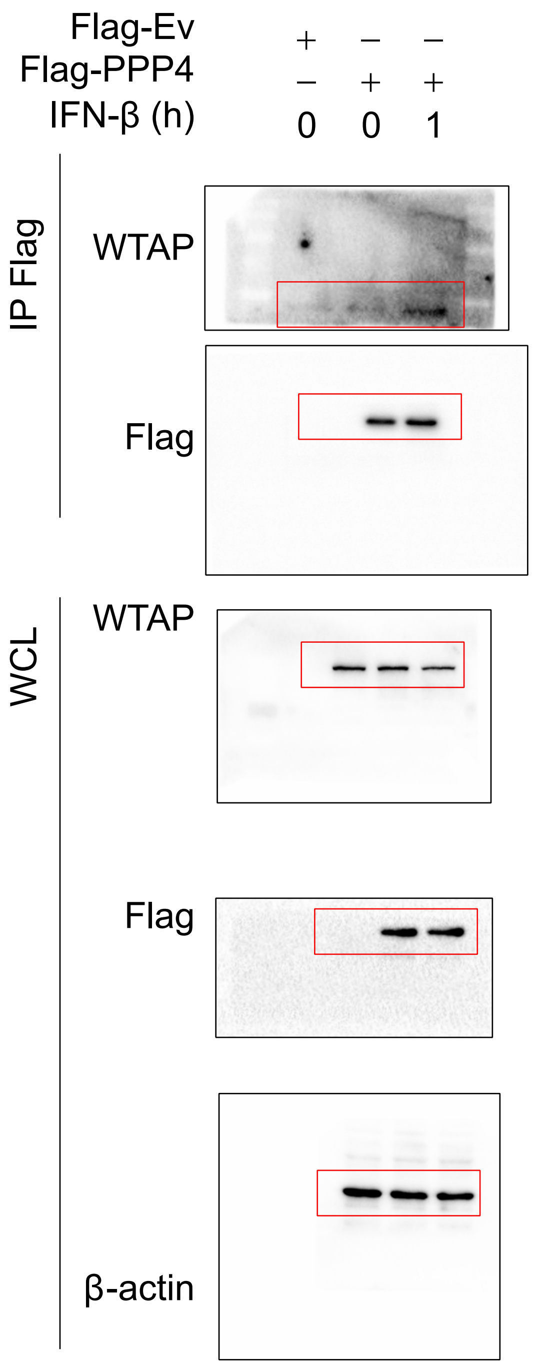

Figure 2-figure supplement 1B, source data 1: Original membranes corresponding to Figure 2-figure supplement 1B.
