# Supplementary figures and images for: Phase transition of WTAP regulates m6A modification of interferon-stimulated genes (part 2 of 2)
Source: eLife. 2025 May 27;13:RP100601. doi: 10.7554/eLife.100601 (PMC12113268; doi:10.7554/eLife.100601)

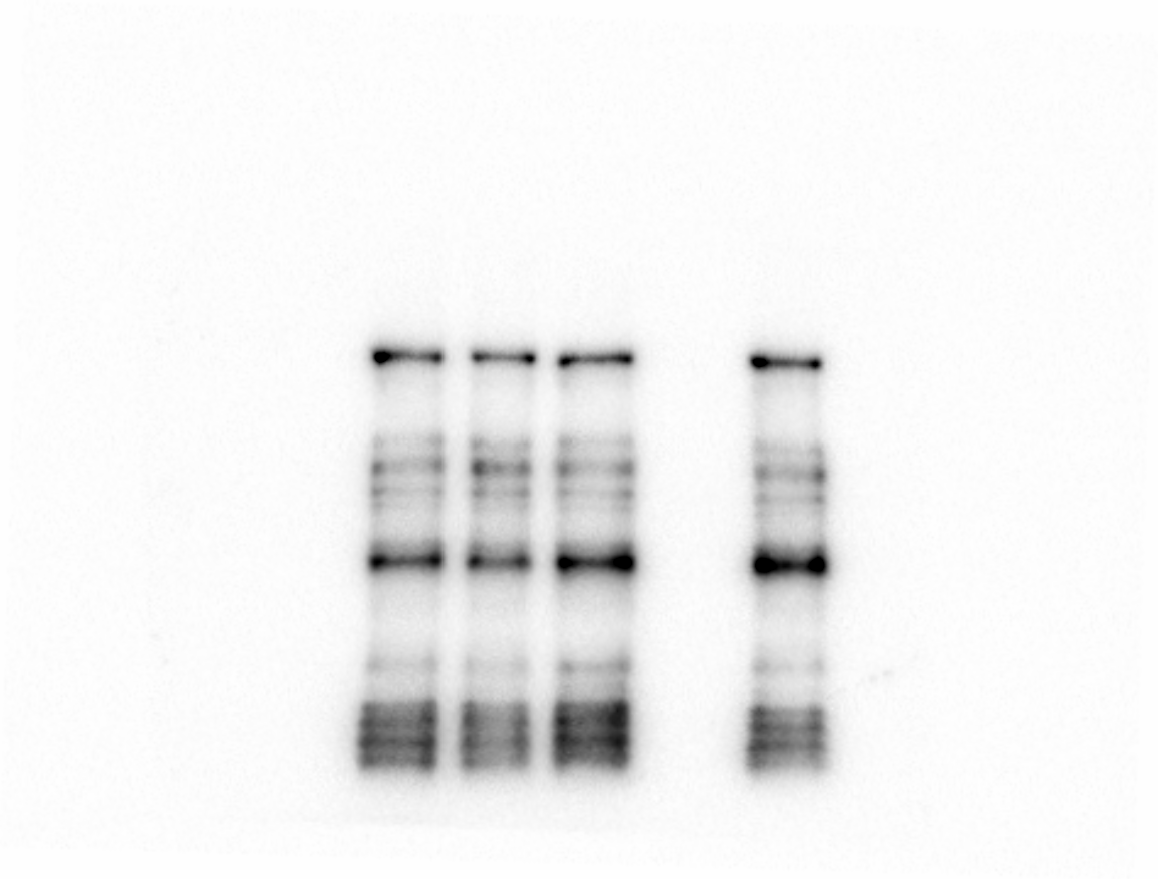

Supplement: Figure 5—figure supplement 1—source data 2. [file elife-100601-fig5-figsupp1-data2.zip › Figure 5-figure supplement 1-Source Data 2/Figure5-figure supplement 1f/CFP-METTL3.tif]

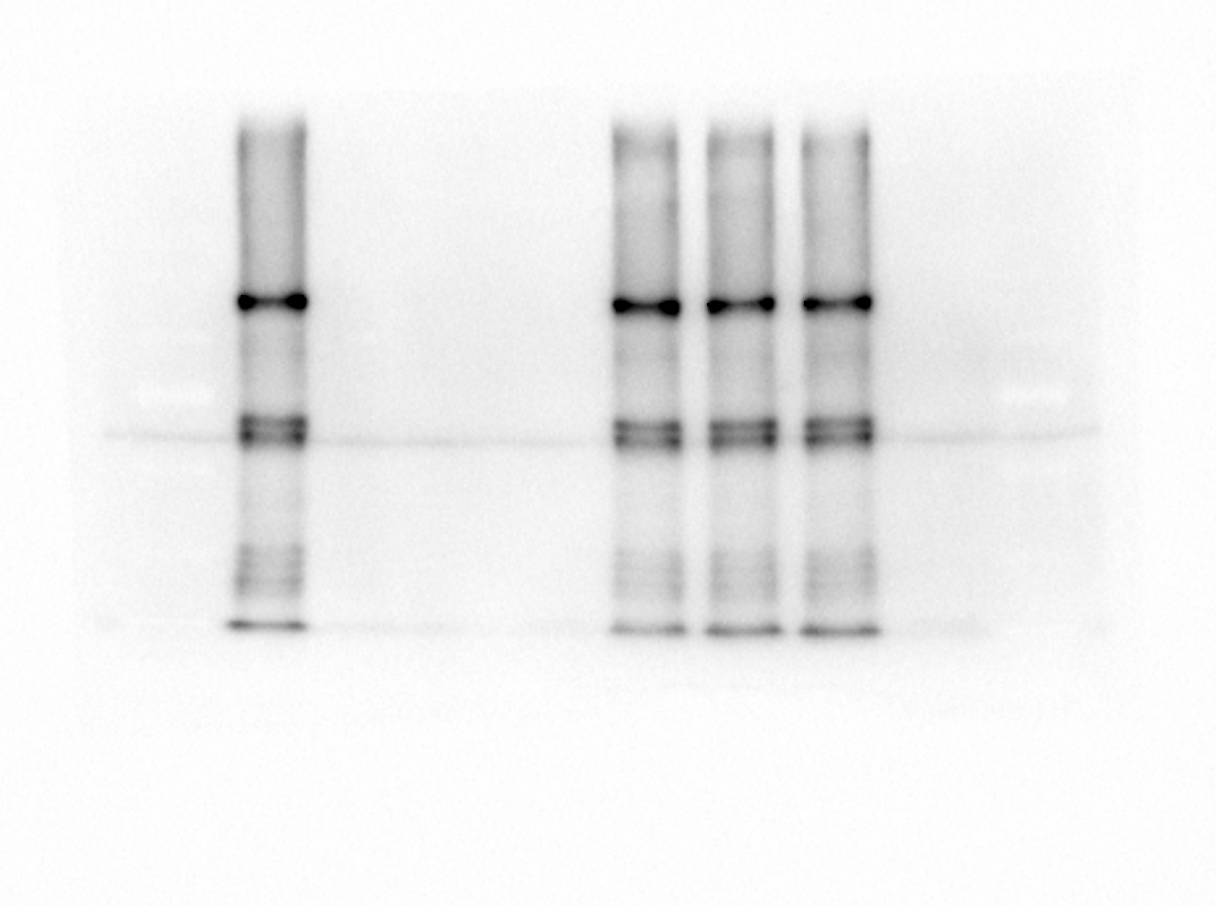

Supplement: Figure 5—figure supplement 1—source data 2. [file elife-100601-fig5-figsupp1-data2.zip › Figure 5-figure supplement 1-Source Data 2/Figure5-figure supplement 1f/GFP-STAT1.tif]

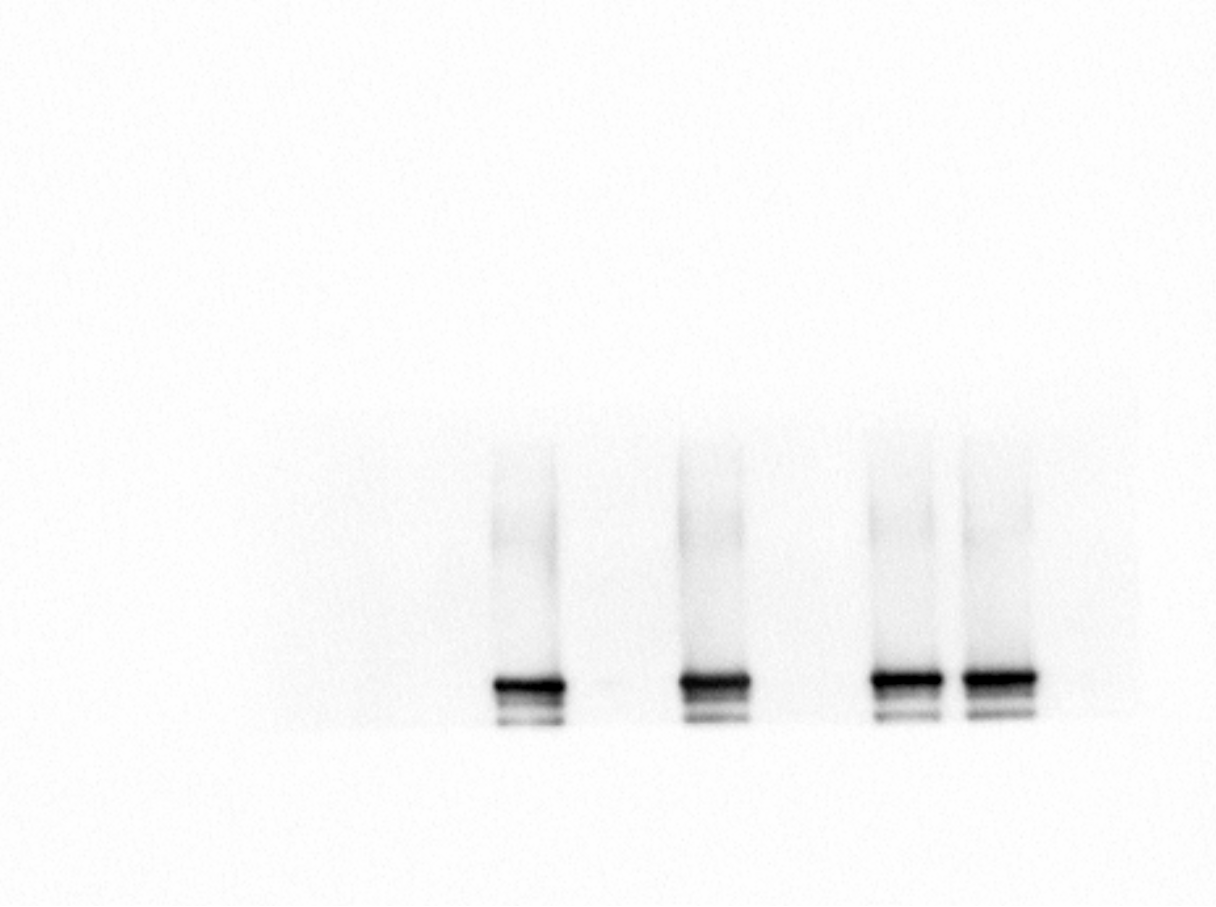

Supplement: Figure 5—figure supplement 1—source data 2. [file elife-100601-fig5-figsupp1-data2.zip › Figure 5-figure supplement 1-Source Data 2/Figure5-figure supplement 1f/mCherry-WTAP.tif]

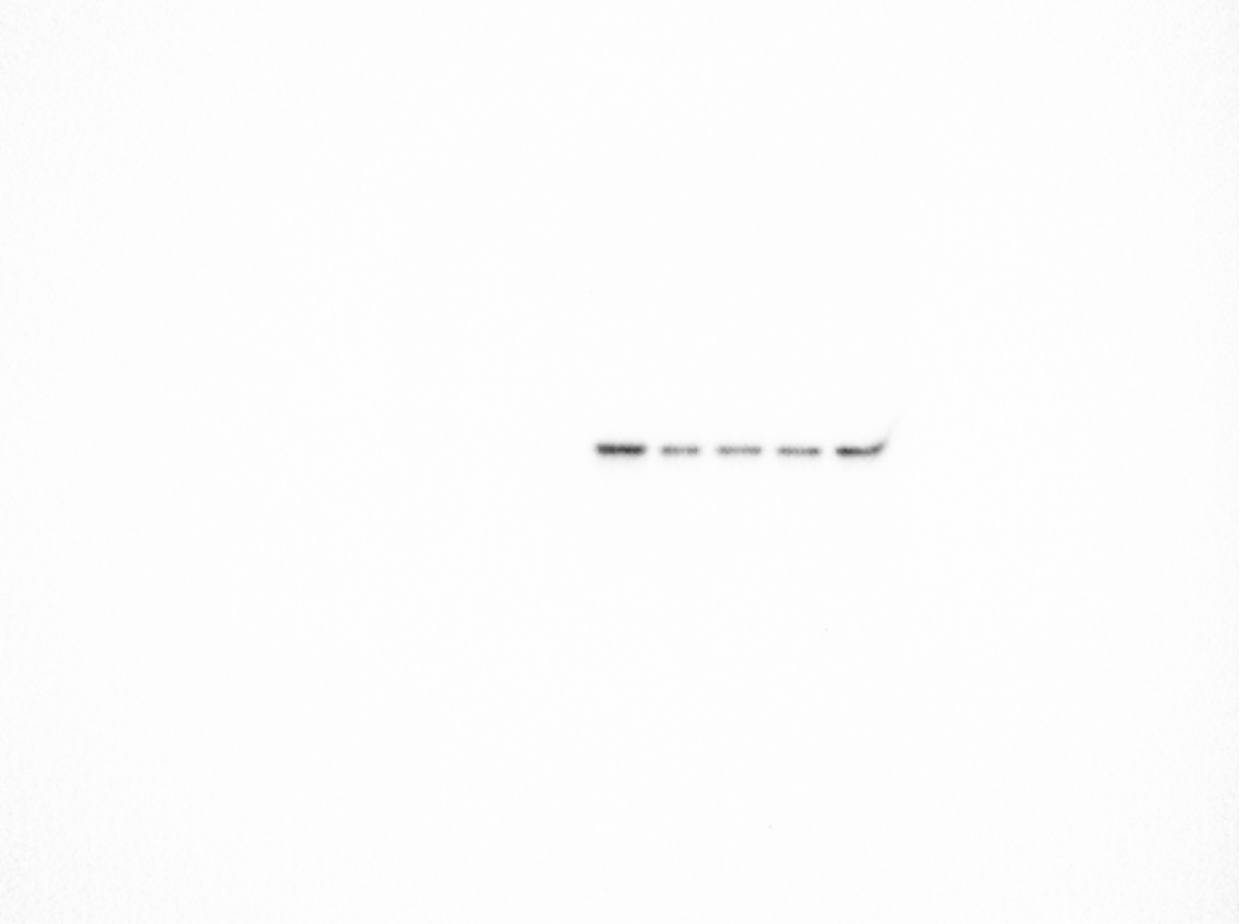

Supplement: Figure 5—figure supplement 1—source data 2. [file elife-100601-fig5-figsupp1-data2.zip › Figure 5-figure supplement 1-Source Data 2/Figure5-figure supplement 1h/IP-pan-p.tif]

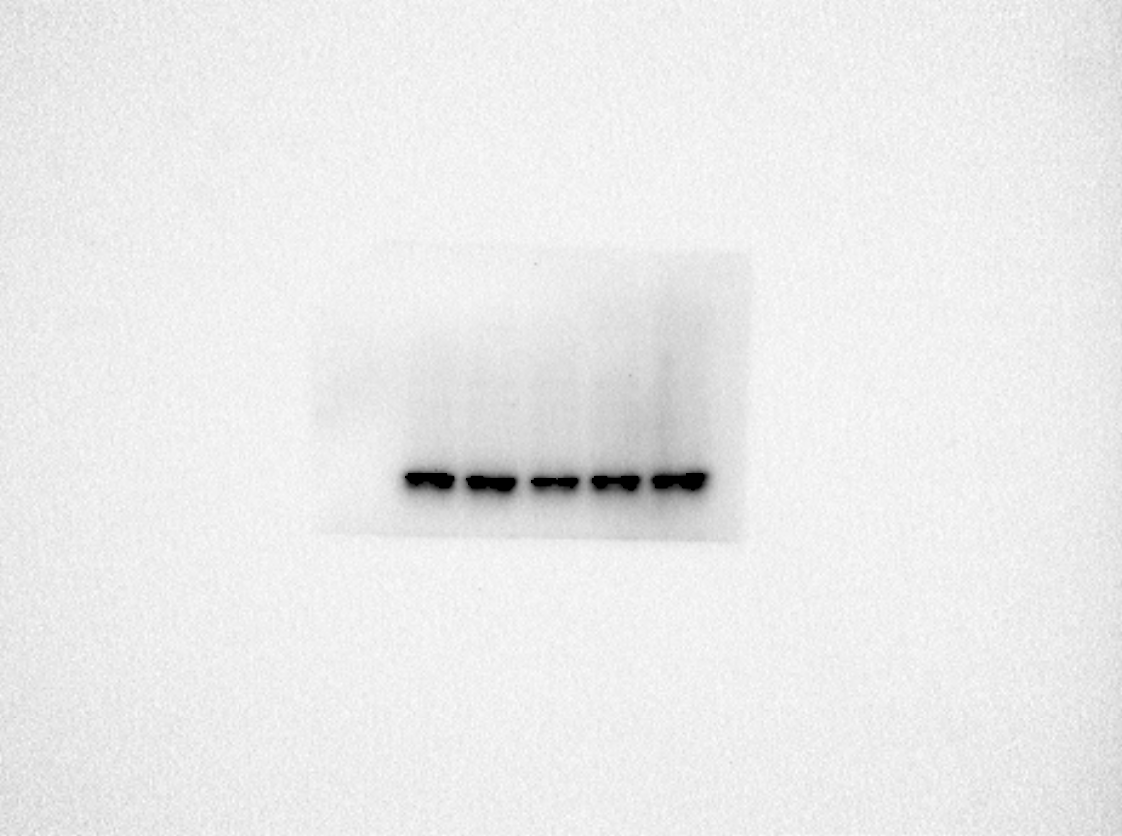

Supplement: Figure 5—figure supplement 1—source data 2. [file elife-100601-fig5-figsupp1-data2.zip › Figure 5-figure supplement 1-Source Data 2/Figure5-figure supplement 1h/IP-WTAP.tif]

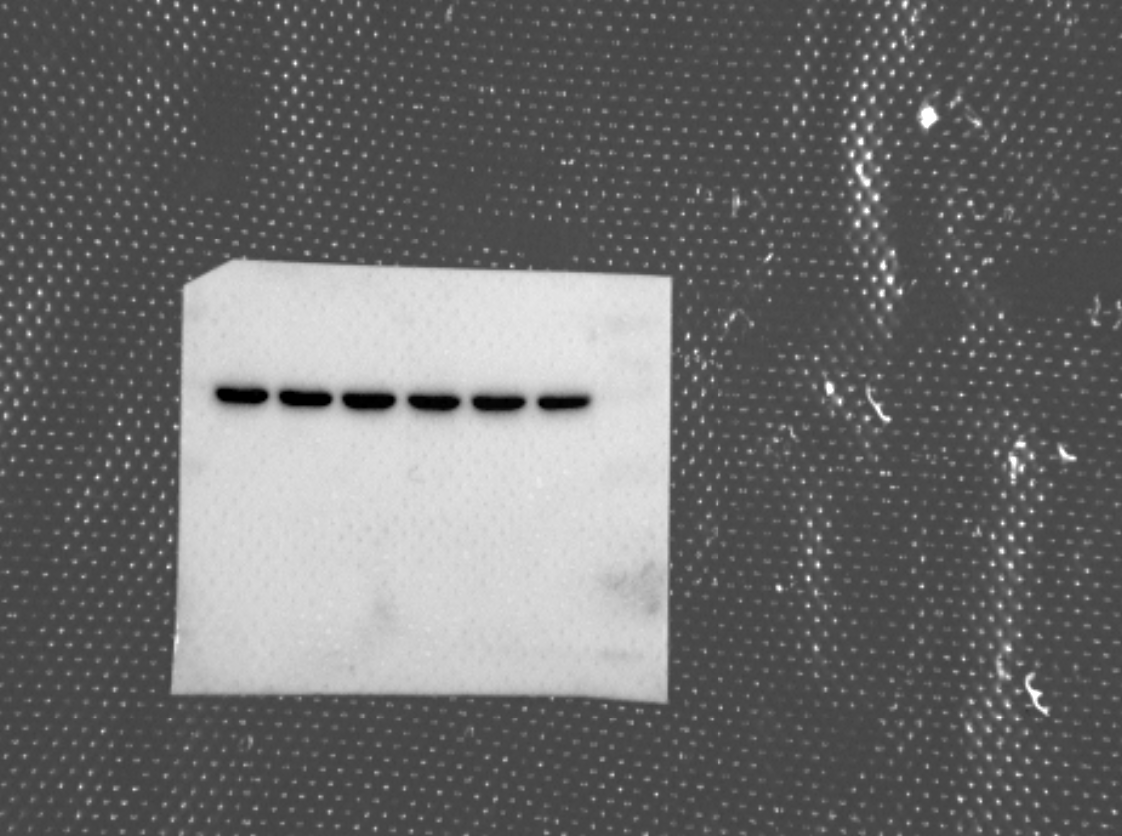

Supplement: Figure 5—figure supplement 1—source data 2. [file elife-100601-fig5-figsupp1-data2.zip › Figure 5-figure supplement 1-Source Data 2/Figure5-figure supplement 1h/actin.tif]

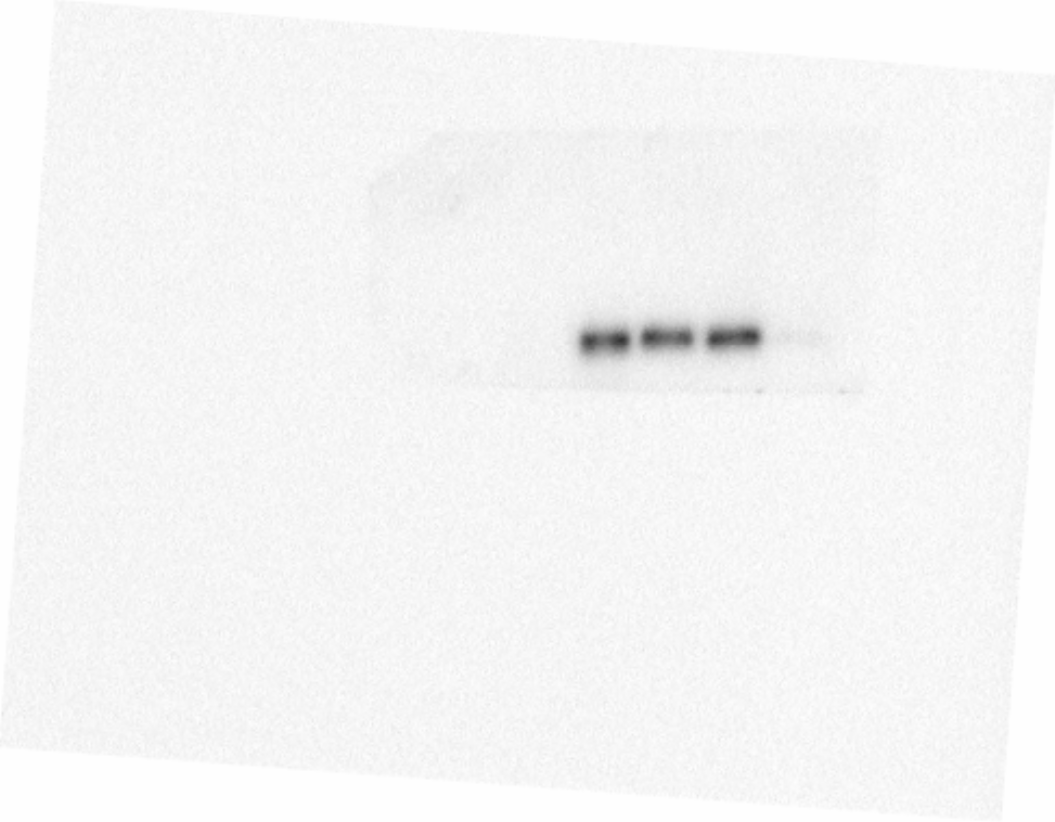

Supplement: Figure 5—figure supplement 1—source data 2. [file elife-100601-fig5-figsupp1-data2.zip › Figure 5-figure supplement 1-Source Data 2/Figure5-figure supplement 1h/pSTAT1.tif]

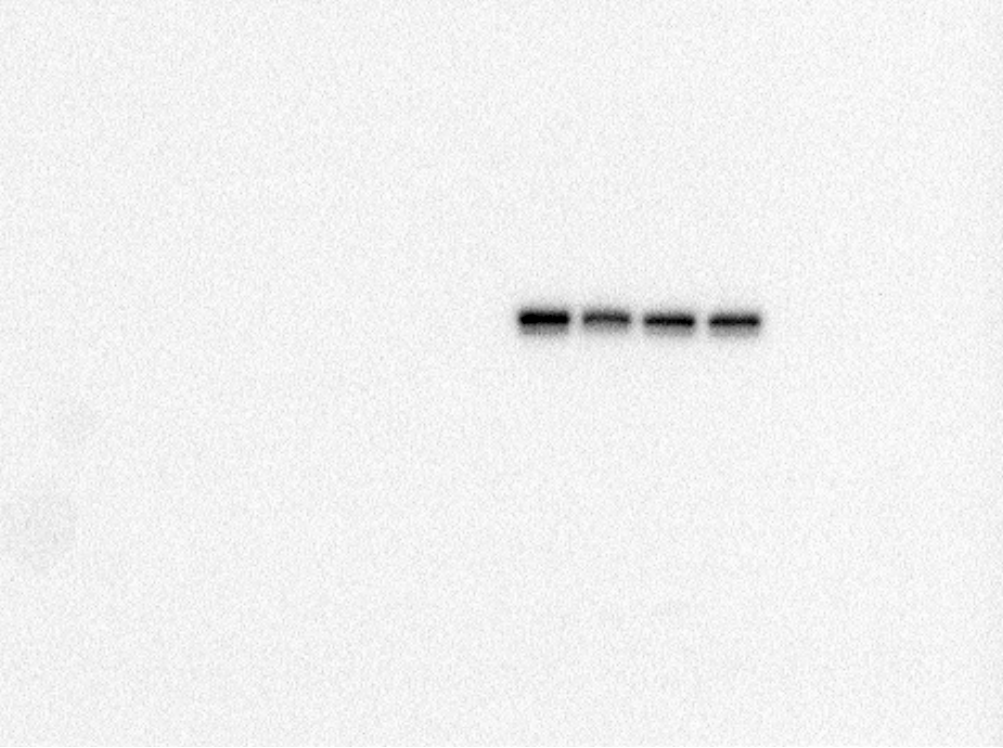

Supplement: Figure 5—figure supplement 1—source data 2. [file elife-100601-fig5-figsupp1-data2.zip › Figure 5-figure supplement 1-Source Data 2/Figure5-figure supplement 1h/STAT1.tif]

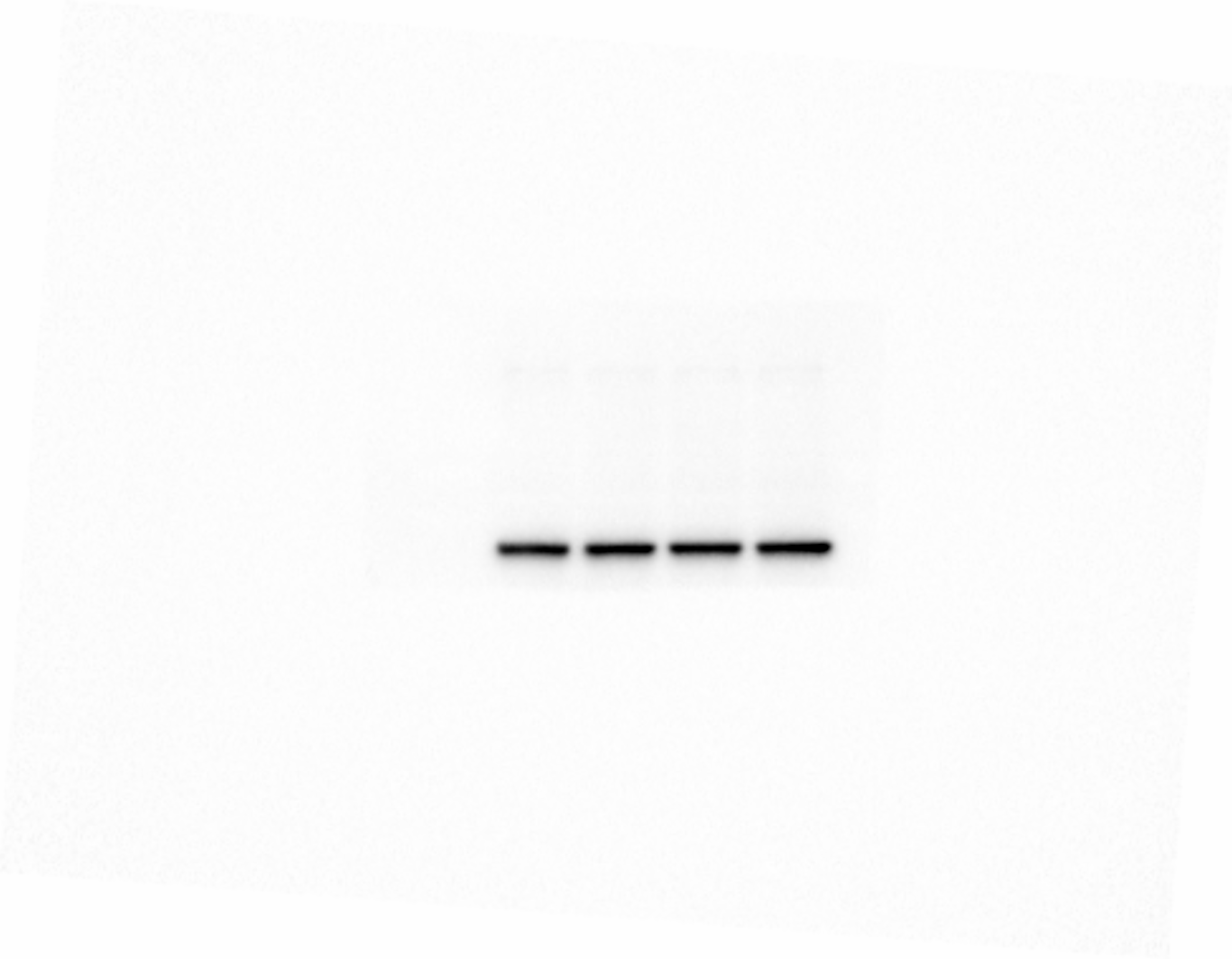

Supplement: Figure 5—figure supplement 1—source data 2. [file elife-100601-fig5-figsupp1-data2.zip › Figure 5-figure supplement 1-Source Data 2/Figure5-figure supplement 1h/WTAP.tif]
