# Supplementary material for: Phase transition of WTAP regulates m6A modification of interferon-stimulated genes
Source: eLife. 2025 May 27;13:RP100601. doi: 10.7554/eLife.100601 (PMC12113268; doi:10.7554/eLife.100601)
Supplement: Supplementary file 1. [file elife-100601-supp1.docx]

**Supplementary File for**

Phase transition of WTAP regulates m^6^A modification of interferon-stimulated genes

Sihui Cai^1,#^, Jie Zhou^2,3,#^, Xiaotong Luo^4,5^, Chenqiu Zhang^1^, Shouheng Jin^1^, Jian Ren^6^, Jun Cui^1^

^1^ MOE Key Laboratory of Gene Function and Regulation, State Key Laboratory of Biocontrol, School of Life Sciences, Sun Yat-sen University, Guangzhou, Guangdong, 510006, China.

^2^ State Key Laboratory of Membrane Biology & Frontier Research Center for Biological Structure, School of Life Sciences, Tsinghua University, Beijing, 100101, China.

^3^ Tsinghua University-Peking University Joint Center for Life Sciences, Beijing, 100101, China.

^4^ Innovation Center of the Sixth Affiliated hospital, School of Life Sciences, Sun Yat-sen University, Guangzhou, 510060, China.

^5^ Guangdong Institute of Gastroenterology, Biomedical Innovation Center, The Sixth Affiliated Hospital, Sun Yat-sen University, Guangzhou, 510060, China.

^6^ State Key Laboratory of Oncology in South China, Cancer Center, Collaborative Innovation Center for Cancer Medicine, School of Life Sciences, Sun Yat-sen University, Guangzhou 510006, China.

^#^ These authors contributed equally: Sihui Cai, Jie Zhou.

**Correspondence:** Jun.Cui (cuij5@mail.sysu.edu.cn)

**Primers for qPCR, RIP-qPCR, MeRIP-qPCR and ChIP-qPCR**

| **Primers for qPCR and RIP-qPCR assay** | |
| --- | --- |
| **Primers** | **Sequence (5’-3’)** |
| **qp-*RPL13A*-F** | GCCATCGTGGCTAAACAGGTA |
| **qp-*RPL13A*-R** | GTTGGTGTTCATCCGCTTGC |
| **qp-*IFIT1*-F** | TCAGGTCAAGGATAGTCTGGAG |
| **qp-*IFIT1*-R** | AGGTTGTGTATTCCCACACTGTA |
| **qp-*IFIT2*-F** | GGAGGGAGAAAACTCCTTGGA |
| **qp-*IFIT2*-R** | GGCCAGTAGGTTGCACATTGT |
| **qp-*OAS1*-F** | AACTGCTTCCGACAATCAAC |
| **qp-*OAS1*-R** | CCTCCTTCTCCCTCCAAAA |
| **qp-*OAS2*-F** | ACCCGAACAGTTCCCCCTGGT |
| **qp-*OAS2*-R** | ACAAGGGTACCATCGGAGTTGCC |
| **qp-*METTL3*-F** | TTGTCTCCAACCTTCCGTAGT |
| **qp*-METTL3*-R** | CCAGATCAGAGAGGTGGTGTAG |
| **qp-*METTL14*-F** | GAACACAGAGCTTAAATCCCCA |
| **qp-*METTL14*-R** | TGTCAGCTAAACCTACATCCCTG |
| **qp-*RBM15*-F** | ATGCCTTCCCACCTTGTGAG |
| **qp-*RBM15*-R** | TCAACCAGTTTTGCACGGAC |
| **qp-*RBM15B*-F** | ACCTGGACCACAGCGTATCT |
| **qp-*RBM15B*-R** | GGGTTGCGACCAATCACTC |
| **qp-*ZC3H13*-F** | GTGCCGTAACTGGCTGAAGA |
| **qp-*ZC3H13*-R** | CCTTTACCACGAGGTGAAGGG |
| **qp-*VIRMA*-F** | ATACTGATGGTCTGGTGCTAAGA |
| **qp-*VIRMA*-R** | TGGAGGGCTTCCATTAAACTGAT |
| **qp-*HAKAI*-F** | TGTTACCCGTGCTTCACTTGA |
| **qp-*HAKAI*-R** | CTTTGGCGGAATATGGCTCAT |
| **qp-*YTHDF1*-F** | ACCTGTCCAGCTATTACCCG |
| **qp-*YTHDF1*-R** | TGGTGAGGTATGGAATCGGAG |
| **qp-*YTHDF2*-F** | AGCCCCACTTCCTACCAGATG |
| **qp-*YTHDF2*-R** | TGAGAACTGTTATTTCCCCATGC |
| **qp-*YTHDF3-*F** | TCAGAGTAACAGCTATCCACCA |
| **qp-*YTHDF3-*R** | GGTTGTCAGATATGGCATAGGCT |
| **qp-*YTHDC1*-F** | AACTGGTTTCTAAGCCACTGAGC |
| **qp-*YTHDC1-*R** | GGAGGCACTACTTGATAGACGA |
| **qp-*FTO*-F** | AACACCAGGCTCTTTACGGTC |
| **qp-*FTO*-R** | TGTCCGTTGTAGGATGAACCC |
| **qp-*ALKBH5*-F** | CGGCGAAGGCTACACTTACG |
| **qp-*ALKBH5*-R** | CCACCAGCTTTTGGATCACCA |
|  |  |
| **Primers for MeRIP-qPCR assay** | |
| **Primers** | **Sequence (5’-3’)** |
| **MeRIP-*IFIT1*-F** | GGCAGAAGCCCAGACTTACC |
| **MeRIP-*IFIT1*-R** | GGGTCCACTTCAAGCACCTT |
| **MeRIP-*IFIT2*-F** | GAGATGTGGTGCCCACTAGG |
| **MeRIP-*IFIT2*-R** | TGGCCAGTGTCTTAGTCCAA |
| **MeRIP-*OAS1*-F** | CAACACAGCCCAGGGATTTC |
| **MeRIP-*OAS1*-R** | CCAGTAGATGCAGAGTTGCTG |
| **MeRIP-*OAS2*-F** | TCACCCTAGCCCCGTACTTT |
| **MeRIP-*OAS2*-R** | AATGAATCGTGACCCCAGCA |
|  |  |
| **Primers for ChIP-qPCR assay** | |
| **Primers** | **Sequence (5’-3’)** |
| **ChIP-*IFIT1*-F** | ATGGTTGCAGGTCTGCAGTT |
| **ChIP-*IFIT1*-R** | TGTAAGCTGTGGGTGTGTCC |
| **ChIP-*IFIT2*-F** | CCGATGAAACATCCCTCTCTGC |
| **ChIP-*IFIT2*-R** | CAAGTGGCCTCTGGTTCCTTTTTG |
| **ChIP-*OAS1*-F** | GCAGTGGGCATGTATTGCTG |
| **ChIP-*OAS1*-R** | CAGCCCAGCCTTTCTCTGAA |
| **ChIP-*OAS2*-F** | ACCCGAACAGTTCCCCCTGGT |
| **ChIP-*OAS2*-R** | ACAAGGGTACCATCGGAGTTGCC |
